# Neoadjuvant relatlimab and nivolumab in resectable melanoma

**DOI:** 10.1038/s41586-022-05368-8

**Published:** 2022-10-26

**Authors:** Rodabe N. Amaria, Michael Postow, Elizabeth M. Burton, Michael T. Tetzlaff, Merrick I. Ross, Carlos Torres-Cabala, Isabella C. Glitza, Fei Duan, Denái R. Milton, Klaus Busam, Lauren Simpson, Jennifer L. McQuade, Michael K. Wong, Jeffrey E. Gershenwald, Jeffrey E. Lee, Ryan P. Goepfert, Emily Z. Keung, Sarah B. Fisher, Allison Betof-Warner, Alexander N. Shoushtari, Margaret Callahan, Daniel Coit, Edmund K. Bartlett, Danielle Bello, Parisa Momtaz, Courtney Nicholas, Aidi Gu, Xuejun Zhang, Brinda Rao Korivi, Madhavi Patnana, Sapna P. Patel, Adi Diab, Anthony Lucci, Victor G. Prieto, Michael A. Davies, James P. Allison, Padmanee Sharma, Jennifer A. Wargo, Charlotte Ariyan, Hussein A. Tawbi

**Affiliations:** 1 Department of Melanoma Medical Oncology, The University of Texas MD Anderson Cancer Center, Houston, TX USA; 2 Department of Medicine, Memorial Sloan Kettering Cancer Center and Weill Cornell Medical College, New York, NY USA; 3 Department of Pathology, The University of California San Francisco, San Francisco, CA USA; 4 Department of Surgical Oncology, The University of Texas MD Anderson Cancer Center, Houston, TX US; 5 Department of Pathology, The University of Texas MD Anderson Cancer Center, Houston, TX USA; 6 Department of Immunology, The University of Texas MD Anderson Cancer Center, Houston, TX USA; 7 Department of Biostatistics, The University of Texas MD Anderson Cancer Center, Houston, TX USA; 8 Department of Pathology, Memorial Sloan Kettering Cancer Center, New York, NY USA; 9 Department of Head and Neck Surgery, The University of Texas MD Anderson Cancer Center, Houston, TX USA; 10 Department of Surgical Oncology, Memorial Sloan Kettering Cancer Center, New York, NY USA; 11 Department of Radiology, The University of Texas MD Anderson Cancer Center, Houston, TX USA

**Keywords:** Melanoma, Cancer immunotherapy

## Abstract

Relatlimab and nivolumab combination immunotherapy improves progression-free survival over nivolumab monotherapy in patients with unresectable advanced melanoma^1^. We investigated this regimen in patients with resectable clinical stage III or oligometastatic stage IV melanoma (NCT02519322). Patients received two neoadjuvant doses (nivolumab 480 mg and relatlimab 160 mg intravenously every 4 weeks) followed by surgery, and then ten doses of adjuvant combination therapy. The primary end point was pathologic complete response (pCR) rate^2^. The combination resulted in 57% pCR rate and 70% overall pathologic response rate among 30 patients treated. The radiographic response rate using Response Evaluation Criteria in Solid Tumors 1.1 was 57%. No grade 3–4 immune-related adverse events were observed in the neoadjuvant setting. The 1- and 2-year recurrence-free survival rate was 100% and 92% for patients with any pathologic response, compared to 88% and 55% for patients who did not have a pathologic response (*P* = 0.005). Increased immune cell infiltration at baseline, and decrease in M2 macrophages during treatment, were associated with pathologic response. Our results indicate that neoadjuvant relatlimab and nivolumab induces a high pCR rate. Safety during neoadjuvant therapy is favourable compared to other combination immunotherapy regimens. These data, in combination with the results of the RELATIVITY-047 trial^1^, provide further confirmation of the efficacy and safety of this new immunotherapy regimen.

## Abstract (web summary)

Patients with resectable clinical stage III or oligometastatic stage IV melanoma were given neoadjuvant relatlimab and nivolumab combination immunotherapy, which induced a high pathologic complete response rate, indicating the efficacy and safety of this regimen.

## Main

Patients with locoregionally advanced, resectable melanoma have a high risk of relapse and death from melanoma^3^. Specifically, patients with clinically detected nodal disease have a risk of melanoma-specific mortality that could be as high as 75%^3^. Although current adjuvant therapy decreases the risk of recurrence by about 50% (BRAF-targeted therapy hazard ratio (HR) 0.49, single agent PD-1 HR approximately 0.54)^4,5^, there has yet to be confirmation of the impact on overall survival^4,6^. In an attempt to intensify therapy beyond single agent anti-PD-1, the Checkmate-915 trial was designed to investigate if the addition of ipilimumab to nivolumab in the adjuvant setting improved recurrence-free survival (RFS) compared to nivolumab alone. The combination of ipilimumab and nivolumab did not improve RFS (HR 0.92) and it significantly increased toxicity (grade 3–4 adverse events (AEs) 43%, compared to 23% for single agent anti-PD-1)^7^, indicating that intensification of adjuvant therapy with ipilimumab and nivolumab in the adjuvant setting is not the optimal approach for improving recurrence outcomes.

Neoadjuvant therapy offers several advantages over upfront surgery and adjuvant therapy, including potential for improvement in clinical outcomes and understanding molecular and immunological mechanisms of treatment response and resistance^8–13^. Additionally, neoadjuvant immunotherapy has demonstrated ability in preclinical models and in human samples to increase expansion of antigen-specific T cells due to the presence of tumour at the time of treatment compared to the expansion seen when the same immunotherapy is administered in the adjuvant setting^14,15^. The neoadjuvant setting also offers the opportunity to intensify therapy with combinations for a short pre-operative course, allowing for a direct estimate of therapeutic efficacy and the ability to inform adjuvant therapy decisions.

One potential limitation of neoadjuvant immunotherapy is delay in curative-intent surgery if grade 3/4 immune-related adverse events (IRAEs) occur during treatment. For example, neoadjuvant administration of 2–3 doses of ipilimumab 3 mg kg^−1^ + nivolumab 1 mg kg^−1^ was associated with 73–90% grade 3/4 toxicities, which led to surgical delays in approximately 27% of patients^15,16^. The OpACIN-NEO trial compared two doses of neoadjuvant therapy with different dosing strategies of ipilimumab and nivolumab. This study demonstrated that ipilimumab 1 mg kg^−1^ with nivolumab 3 mg kg^−1^ showed an at least equivalent pCR rate (57%) to the ipilimumab 3 mg kg^−1 ^+ nivolumab 1 mg kg^−1^ regimen (47%), but with a lower (20% versus 40%) incidence of grade 3/4 toxicities^17^. These data highlight the goal of identifying new regimens that enhance pathologic responses and reduce risk of recurrence with improved toxicity profiles.

The lymphocyte-activation gene 3 (LAG-3) regulates an inhibitory immune checkpoint limiting T cell activity and is a marker for T cell exhaustion^18,19^. Relatlimab is a human IgG4 LAG-3-blocking monoclonal antibody that restores the effector function of exhausted T cells and has been investigated in both checkpoint inhibitor-naïve (NCT03470922)^1^ and refractory metastatic melanoma (NCT01968109)^20^. In the randomized phase 2/3 RELATIVITY-047 study, the combination of relatlimab with nivolumab in patients with treatment-naïve unresectable stage III or stage IV metastatic melanoma demonstrated significant improvement in progression-free survival compared to single agent nivolumab (HR 0.78 (95% confidence interval (CI), 0.64–0.94)). Moreover, the combination was well tolerated with 21.1% of patients experiencing grade 3/4 treatment-related AEs^1^. Given its efficacy and favourable toxicity profile, this combination therapy received US Food and Drug Administration approval for use in patients with metastatic melanoma on 18 March 2022.

**Fig. 1:**
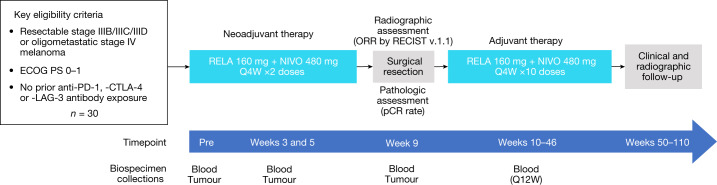
Study design. Eligible patients receive two doses of relatlimab 160 mg with nivolumab 480 mg intravenously every 4 weeks (Q4W) in the neoadjuvant setting and then have repeat imaging for calculation of RECIST response. Surgery takes place at week 9 for evaluation of pathologic response. Patients receive up to ten doses of relatlimab 160 mg and nivolumab 480 mg every 4 weeks in the adjuvant setting and are followed for 2 years for evidence of recurrence. Blood and tumour are collected during screening, at weeks 3, 5 and at time of surgery at week 9. Blood is collected every 12 weeks (Q12W) in the adjuvant setting. ECOG PS, Eastern Cooperative Oncology Group Performance Status; RELA, relatlimab; NIVO, nivolumab; ORR, objective response rate; RECIST, Response Evaluation Criteria in Solid Tumors.

Our group previously published our experience of a randomized, investigator-initiated clinical trial of either single agent nivolumab (240 mg intravenously every 2 weeks up to four doses) or nivolumab 1 mg kg^−1^ with ipilimumab 3 mg  g^−1^ (intravenously every 3 weeks up to three doses) in the neoadjuvant setting^16^. In this trial, we concluded that although neoadjuvant single agent nivolumab was safe (8% grade 3/4 toxicities), its efficacy was modest (25% pCR rate). Although the combination of nivolumab with ipilimumab was effective with a 45% pCR rate, the toxicity was prohibitively high with 73% grade 3/4 toxicities^16^. Given these data and the early closure of the study due to suboptimal performance of both treatment arms, our team sought to evaluate new immunotherapy combinations with the intention of preserving pathologic response while minimizing toxicities. We opened a new arm to this existing prospective clinical trial to determine pCR rate, safety and efficacy of the relatlimab and nivolumab combination in patients with resectable clinical stage III or oligometastatic stage IV melanoma (Clinicaltrial.gov number NCT02519322) (Fig. 1). Here we report the clinical results and immune profiling of this neoadjuvant therapy combination.

## Patient characteristics

**Fig. 2:**
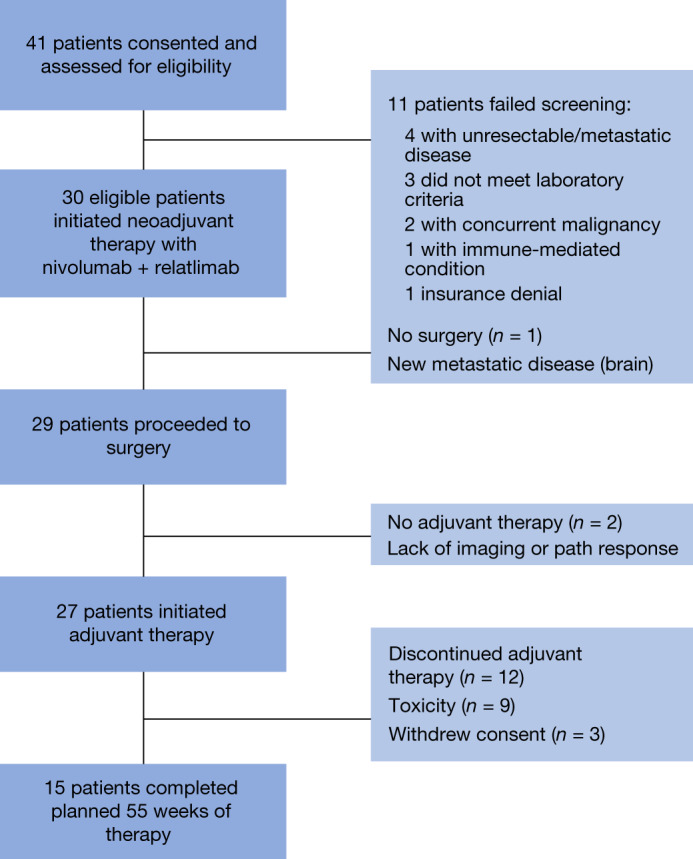
Consort diagram and patient disposition. A total of 41 patients were screened for protocol and there were 11 screen failures and 30 patients were eligible to initiate therapy. After completion of neoadjuvant therapy, one patient developed distant metastases and did not proceed to surgery. Twenty-nine patients proceeded to surgery and 17 patients (57%) achieved a pCR. Twenty-seven patients initiated adjuvant therapy and 15 went on to complete entire duration of treatment. path, pathologic.

From 19 September 2018 to 23 September 2020, 41 patients were consented and 30 passed screening evaluations and were treated at MD Anderson Cancer Center and Memorial Sloan Kettering Cancer Center. The most common reasons for screen failure included lack of resectable disease as determined by multidisciplinary review (*n* = 4 patients) and laboratory values outside the specified criteria (*n* = 3 patients) (Fig. 2).

The median age of treated patients was 60 (range 35–79) and 63% of patients were male (Extended Data Table 1). Melanoma clinical stage was 60% stage IIIB, 26% IIIC, 7% IIID and 7% M1A by the American Joint Committee on Cancer 8th edition criteria^3^. Thirty-three per cent of patients had de novo clinical stage III or oligometastatic stage IV melanoma, and 67% had prior melanoma surgery. Only 17% of patients had *BRAF-*mutated melanoma, probably due to enrolment on a competing neoadjuvant trial specific for patients with *BRAF-*mutated disease. Only one patient had prior systemic therapy (BRAF and MEK inhibition). The median target lesion sum of diameters was 26 mm (Extended Data Table 1).

## Patient disposition

Of the 30 treated patients, 29 were able to receive the planned two doses of neoadjuvant relatlimab and nivolumab. One patient received only one dose due to asymptomatic troponin elevations with concern for myocarditis, which was eventually determined to not be attributable to neoadjuvant immunotherapy after the patient underwent myocardial biopsy and was able to proceed safely to surgery. One patient did not proceed to surgery due to development of distant metastatic disease during neoadjuvant therapy. Of the 29 patients that underwent surgery, 27 patients proceeded to surgery as scheduled at week 9; one patient was delayed due to the aforementioned myocarditis toxicity concern and one patient was delayed due to SARS-CoV2 pandemic-related hospital surgery restrictions. Twenty-seven patients proceeded with adjuvant therapy and two patients elected to not proceed with adjuvant therapy due to suboptimal pathologic and imaging response. Fifty-six per cent of patients completed the entire duration of protocol therapy, 33% of patients discontinued adjuvant therapy due to toxicity and 11% of patients withdrew consent during adjuvant therapy (Fig. 2). Currently, all patients are off protocol therapy.

## Clinical activity

**Fig. 3:**
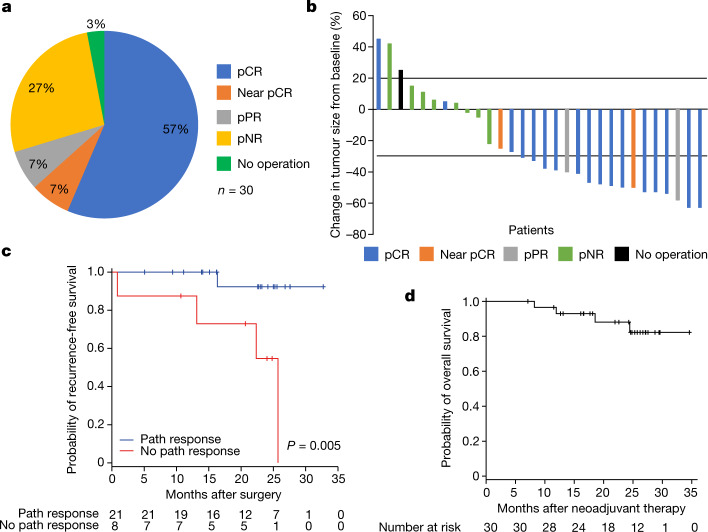
Response data and long-term outcomes. **a**, Breakdown of pathologic responses for the 29 patients who underwent surgery as interpreted by the guidelines of the INMC. Result details (values in chart rounded): no operation, 1 of 30 patients (3.33%);pCR, 17 of 30 patients (56.67%); near pCR, 2 of 30 patients (6.67%);pPR, 2 of 30 patients (6.67%);pNR, 8 of 30 patients (26.67%). **b**, Waterfall plot of neoadjuvant response as per RECIST 1.1 criteria with colour coding indicating pathologic response. pCR indicates lack of viable tumour. Near pCR indicates greater than 0% but less than or equal to 10% viable tumour, pPR is greater than 10% to less than or equal to 50% viable tumour and pNR is greater than 50% viable tumour. **c**, Probability of being relapse-free based on any pathologic response versus no pathologic response. **d**, Overall survival curves for the entire cohort.

Of the 30 patients enroled, 29 patients underwent surgery (97%), 17 (57%; 95% CI, 37–75%) achieved pCR, two (7%) near pCR (defined as greater than 0% but less than or equal to 10% viable tumour), two (7%) partial pathologic response (pPR; defined as greater than 10% to less than or equal to 50% viable tumour) and eight (27%) no pathologic response (pNR; defined as greater than 50% viable tumour) (Fig. 3a). A major pathologic response (pCR + near pCR) was achieved in 63% of patients and any pathologic response (pCR + near pCR + pPR) in 70% of patients^2^.

The radiographic overall response rate was 57% (all partial responses (PRs); 33% had stable disease (SD) and 10% had progressive disease (PD)(Fig. 3b)) in the intention-to-treat population. Pathologic response was frequently disconcordant with radiographic response at 8 weeks. For example, of the 19 patients who achieved major pathologic response (pCR and near pCR), one patient had radiographic PD, three had SD and 15 had PR. Of the eight patients with pNR, only one had radiographic PD and seven had SD. In the 16 patients with tumour sum of diameters at the median or higher (at least 26 mm), there was a mix of Response Evaluation Criteria in Solid Tumors (RECIST; 6% PD, 38% SD, 56% PR) and pathologic responses (38% pNR, 6% pPR, 6% near pCR, 50% pCR), indicating that baseline tumour burden did not correlate directly with pathologic or radiographic response.

**Extended Data Fig. 1:**
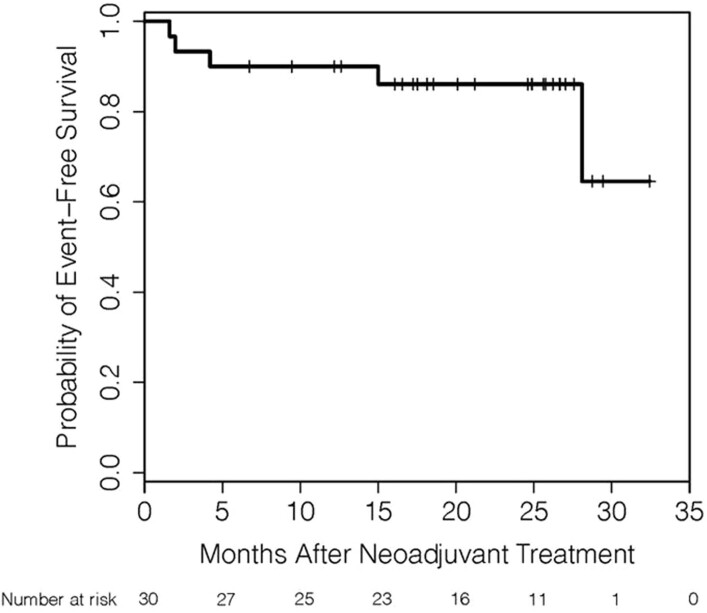
Probability of being event-free for all patients who received study treatment.

**Extended Data Fig. 2:**
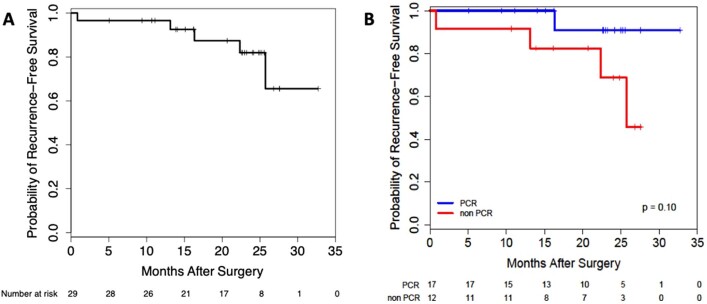
Probability of being recurrence-free. **A**) Probability of being recurrence-free for all patients who underwent surgery. **B**) Probability of being recurrence-free based on pathologic complete response versus non-pathologic complete response (*P* = 0.10). Source data

With a median follow-up of 24.4 months (range 7.1–34.6 months) for the 30 treated patients, 1- and 2-year event-free survival rates (time from treatment initiation to recurrence in all patients) were 90% and 81%, respectively (Extended Data Fig. 1). The 1- and 2-year RFS rates (time from surgery to recurrence in patients that underwent surgery) were 97% and 82%, respectively (Extended Data Fig. 2a). The 1- and 2-year RFS rates were 100% and 91% for patients with pCR, compared to 92% and 69% for those without pCR (*P* = 0.10) (Extended Data Fig. 2b). The 1- and 2-year RFS rates were 100% and 92% for patients with any pathologic response, compared to 88% and 55% for those without a pathologic response (*P* = 0.005) (Fig. 3c). The 1- and 2-year overall survival rates for all patients were 93% and 88% (Fig. 3d).

Of the three patients with RECIST PD to neoadjuvant therapy, one patient developed distant metastases (brain) and did not undergo surgery. The two other RECIST PD patients appeared to progress locally in the involved nodal basin only, and complete surgical resection was achieved for both. One of these patients did not proceed with adjuvant therapy due to pNR and patient/physician decision; the other achieved a pCR, proceeded with adjuvant therapy and completed protocol therapy without disease recurrence (Fig. 2). Two patients (both pNR) experienced local recurrence in soft tissue adjacent to site of prior surgical resection at 3 and 14 months after completion of all ten doses of adjuvant therapy. One patient with pCR reportedly experienced unconfirmed disease progression in the brain and passed away 14 months after surgery.

## Safety

There were no grade 3/4 IRAEs during the 8 weeks of neoadjuvant therapy (Extended Data Table 2). Twenty-six per cent of patients developed grade 3/4 IRAEs in the adjuvant setting (from week 9 and beyond) (Extended Data Table 2). Overall, 33% of patients elected to discontinue adjuvant therapy due to any toxicity (most commonly transaminitis). Although there were asymptomatic troponin elevations, no patients experienced symptomatic troponin elevations, myocarditis or other cardiac toxicity attributable to study medications as assessed by cardiology consultation. The most frequent IRAE was secondary adrenal insufficiency (23%), with none of the patients experiencing adrenal recovery to date.

## Correlative studies

**Fig. 4:**
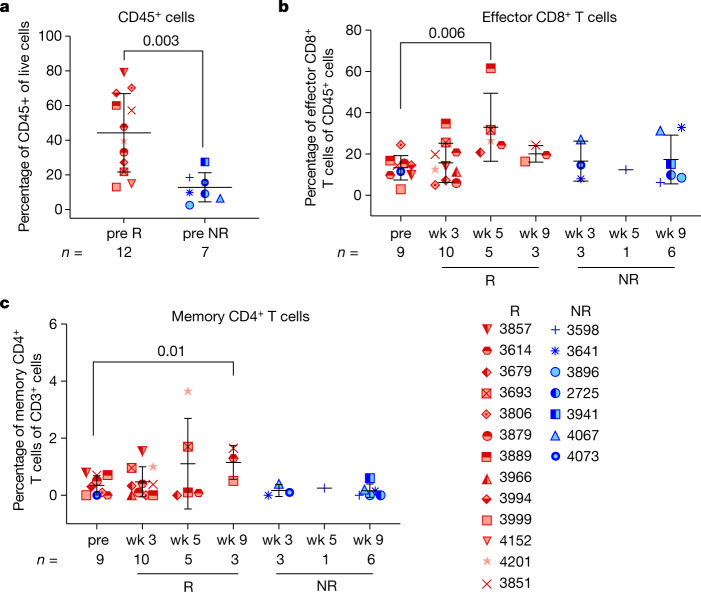
Correlative analyses in tumour specimens. Tumour tissue samples harvested from patients at baseline, and post relatlimab and nivolumab treatment were analysed in a single experiment by CyTOF (**a**–**c**). **a**, Frequency of CD45^+^ cells was assessed through manual gating. **b**, Frequency of an effector CD8^+^ T cell subset (CD3^+^CD8^+^CD45RO^low^) in unsupervised clustering is shown. **c**, Frequency of a memory CD4^+^ subset (CD45RO^+^ICOS^+^ TCF7^+^BTLA^+^CD28^+^TIGIT^+^) was determined by unsupervised clustering. Data shown in **a**–**c** are mean ± s.d., and *n* values are indicated in the figure. *P* values shown in each graph were calculated by two-tailed unpaired *t*-test, with no multiple comparisons. Red indicates pathologic responders; blue, non-responders. CyTOF, mass cytometry; NR, non-responder; R, responder; wk, week. Source data.

**Extended Data Fig. 3:**
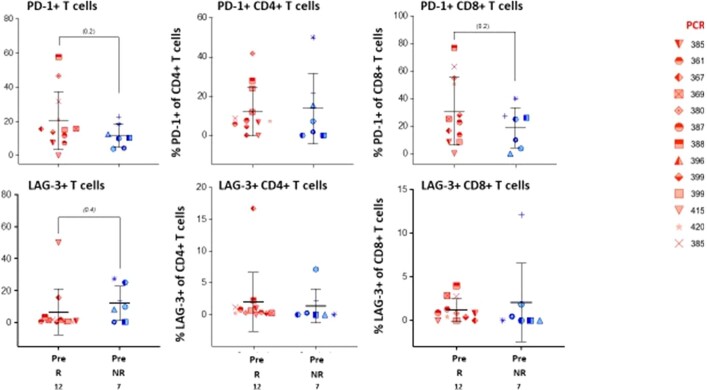
PD-1 and LAG-3 levels in baseline tumour. Tumour infiltrating immune cells were assayed via CyTOF and analysed by manual gating for frequency of **A**) PD-1 and **B**) LAG-3 levels in T cells prior to treatment. Red, pathologic responders; blue, pathologic non-responders. Data are mean +/− SD; *P* values where shown were determined by two-tailed unpaired *t*-test, with no multiple comparisons. *n* values for each group/timepoint are indicated in each graph. Source data

**Extended Data Fig. 4:**
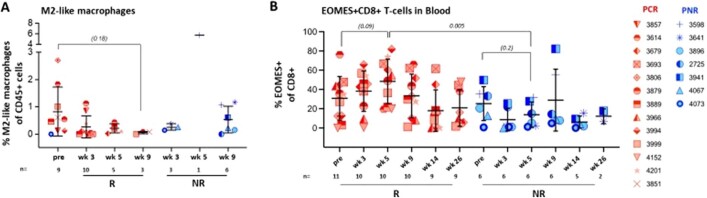
M2-like macrophages in tumour and EOMES+ CD8+ T cells in blood. **A**) Frequency of an M2-like macrophage subset (CD68+ HLA-DR+ CD14+ VISTA+ CD163+ CD45RO+ PD-L1+) was determined by unsupervised clustering of CyTOF data from a single experiment. **B**) Frequency of EOMES+ CD8+ T cells. PBMCs isolated from blood samples were analysed by flow cytometry from a single experiment. Data are mean +/− SD; *P* value was determined by two-tailed unpaired *t*-test, with no multiple comparisons. *n* values for each group/timepoint are indicated in each graph. Red indicates pathologic responders; blue, non-responders.

Biomarker analysis focused on characterizing immune cell subsets in the tumour microenvironment and peripheral blood was performed by mass cytometry (CyTOF) and flow cytometry. LAG-3 and PD-1 levels in baseline tumour samples did not correlate with pathologic response (Extended Data Fig. 3). In tumours, the frequency of CD45^+^ cells was higher in pretreatment samples of responders, defined as patients with less than 50% tumour viability at surgery, compared to pretreatment samples of non-responders (NRs; greater than or equal to 50% tumour viability) (Fig. 4a) by CyTOF. Unsupervised clustering identified an effector CD8^+^ T cell subset (CD8^+^CD45RO^low^) and a memory CD4^+^ T cell subset (CD4^+^CD45RO^+^TCF7^+^CD28^+^BTLA^+^TIGIT^+^) that were increased in posttreatment tumour specimens versus pretreatment in patients with favourable response (Fig. 4b,c). The increases in these cell populations were not appreciated in the NR patient group, although it should be noted that the number of evaluable specimens was low in this group (Fig. 4b,c). By contrast, the frequency of an M2-like macrophage subset decreased in tumours after treatment in patients with favourable response (Extended Data Fig. 4a). In blood, there was a trend for increased EOMES^+^CD8^+^ T cells in patients with favourable versus non-favourable response after treatment, with largest differences seen at week 5 posttreatment (Extended Data Fig. 4b).

## Discussion

In patients with resectable clinical stage III or oligometastatic stage IV melanoma, neoadjuvant relatlimab with nivolumab resulted in high pCR rate (57%; 95% CI, 37–75%) and improvement in the 2-year RFS rate in patients who achieved any pathologic response compared to those without a pathologic response (*P* = 0.005). The lower limit CI (37%) exceeded the minimum target of 30% in the study design. This regimen was tolerated well in the neoadjuvant setting, with 26% grade 3 toxicities noted with continued dosing in the adjuvant setting. In patients with pathologic response, increased immune cell infiltration was identified at baseline and decreased M2 macrophages were demonstrated over the course of neoadjuvant therapy.

The first two randomized arms of this trial evaluated both single agent nivolumab and the combination of ipilimumab 3 mg kg^−1^ and nivolumab 1 mg kg^−1^. Twenty-seven per cent of patients treated with ipilimumab 3 mg kg^−1^ and nivolumab 1 mg kg^−1^ required surgical delays of 1–10 weeks due to need for steroids and prolonged steroid taper^16^. With no grade 3/4 IRAEs observed in the neoadjuvant setting and no confirmed toxicity-related surgical delays, the combination of nivolumab and relatlimab now provides complementary information and demonstrates a highly effective regimen with manageable toxicities in the neoadjuvant setting.

Although there were no grade 3/4 IRAEs in the neoadjuvant setting, 26% grade 3/4 toxicities were experienced in the adjuvant setting. The most common IRAE observed was secondary adrenal insufficiency. As 33% of patients discontinued therapy before the planned full year of treatment, due to toxicity, it raises questions of whether continued dosing in the adjuvant setting is necessary following pathologic response to neoadjuvant therapy. Additionally, none of the patients who stopped therapy early due to toxicity have experienced a recurrence event. There is not clear consensus on the need for the adjuvant phase of therapy within neoadjuvant trials, with completed or ongoing trials including complete omission of any adjuvant therapy, use of adjuvanttherapy only in poor responders or adjuvant therapy to complete 1 year of treatment^8,15–17,21–23^, Additionally, the use of adjuvant therapy can certainly affect the RFS and can cloud the interpretation of neoadjuvant therapy data. Understanding the contribution of adjuvant immunotherapy following immunotherapy in the neoadjuvant setting to clinical benefit remains an active area of research interest.

The historic dogma in neoadjuvant chemotherapy emphasized pCR as the critical end point correlating with the most durable clinical outcomes^11–13^. This was similarly appreciated in the International Neoadjuvant Melanoma Consortium (INMC) pooled analysis of neoadjuvant BRAF/MEK inhibitor use in patients with clinical stage III melanoma, showing that achieving a pCR, but not a pPR, correlated with improved RFS^9,22,23^. Although the pCR end point may still be appropriate for neoadjuvant chemotherapy or molecularly targeted therapy, our data provide further evidence that in the context of neoadjuvant immunotherapy in melanoma, any pathologic response (less than 50% viable tumour) is associated with favourable long-term clinical outcomes (Fig. 3c)^9,16,17,21^. Similar patterns of improved clinical responses with any pathologic response are being appreciated in neoadjuvant immunotherapy trials across solid tumours^24–26^.

Although baseline LAG-3 and PD-1 levels in tumour samples did not correlate with response, we observed increased frequencies of memory CD4^+^ and effector CD8^+^ T cells in the posttreatment tumour specimens of patients with favourable treatment response. These findings are concordant with previous studies in which responses to anti-PD-1 were associated with higher CD8^+^ T cells^15–17,21,27,28^. Furthermore, we observed a reduction in M2-like macrophages with treatment only in the patients that achieved a pathologic response, possibly serving as a target to further improve responsiveness to this regimen, and/or to further evaluate in other studies of nivolumab plus relatlimab^29^. Analysis of longitudinal peripheral blood specimens by flow cytometry revealed higher frequency of EOMES^+^CD8^+^ T cells in posttreatment samples of responding patients, suggesting CD8^+^ T cells expressing EOMES could contribute to tumour regression. This supports a potentially critical role of EOMES for antitumour activity of CD8^+^ T cells, as previously described^30^. These data indicate that a higher frequency of total immune cell infiltration, as well as increased specific effector CD4^+^ and CD8^+^ T cell subsets, with a concomitant decrease in suppressive myeloid cells in the tumour microenvironment, correlate with clinical response to this regimen in the neoadjuvant setting. It should be noted that the number of usable samples in the NR patients was low, which limits comparative correlative analyses in this study.

We acknowledge that the study is limited by its small sample size and that these results are preliminary, based on findings at two academic research institutions. However, the cohort evaluated in this study (*n* = 30) is largely similar to the individual arms in the OpACIN-NEO study and to other single-arm neoadjuvant immunotherapy trials^17,21,23–26^. With a median follow-up of 24 months, we also acknowledge that additional follow-up is needed to fully assess clinical impact and the durability of responses. However, this initial data is encouraging, and the pooled analyses of melanoma neoadjuvant trials support the importance of pathologic response rates as an early predictor of durable benefit^9^. Similarly, additional translational studies beyond the scope of this manuscript are planned, including RNA sequencing for broad assessment of additional immune signatures and populations that have been implicated in immunotherapy resistance^28,31^.

In summary, neoadjuvant relatlimab and nivolumab is a highly active regimen that achieves a 70% pathologic response rate with a favourable safety profile in patients with high-risk, resectable clinical stage III or oligometastatic stage IV melanoma. These data are complementary to the RELATIVITY-047 study in patients with unresectable metastatic melanoma, and together further support the promise of this new combination immunotherapy regimen in this disease.

## Methods

### Patients

Eligible patients were 18 years or older with clinical stage III or oligometastatic (less than three organ sites with metastases) stage IV melanoma with lesions that were measurable by RECIST 1.1 (ref. ^32^). Resectable clinical stage III melanoma was defined as clinically detectable, RECIST-measurable lymph node disease with or without regional in-transit or satellite metastases and without distant metastases. Resectability of stage III and IV disease was verified via multidisciplinary conference. Patients with recurrent melanoma or de novo American Joint Committee on Cancer 8th edition^3^ clinical stage III or IV disease were considered eligible, and all melanoma subtypes, including uveal, mucosal or acral, were eligible for enrolment. All patients had Eastern Cooperative Oncology Group performance status of 0 or 1 with normal organ function and no contra-indication to surgery. Patients requiring active immunosuppressive therapy, or who had active autoimmune or infectious disease, or with uncontrolled cardiovascular disease or ongoing concurrent malignancy were excluded.

### Study design

This investigator-initiated, prospective study was conducted at two academic medical centres in the United States. Patients received two intravenous fixed doses of relatlimab 160 mg with nivolumab 480 mg at 4-week intervals. Surgery was planned 9 weeks after treatment initiation. Patients were given up to ten doses of the combination starting 4–6 weeks after surgery to complete a total of 12 doses. Patients were followed for 2 years postsurgery for any evidence of disease recurrence (study design details are provided in Fig. 1).

The primary end point was determination of pCR (defined as no viable tumour upon pathologic evaluation at surgery) rate^2^. For this exploratory biomarker study, a pathologic response rate of 30% was suggested for patients treated with this combination. Assuming this true pCR rate, the probability of at least 5 out of 30 patients experiencing a pCR is 0.97. Secondary end points included RECIST 1.1 overall response rate, safety, RFS, event-free survival, overall survival and correlation of immune profiling with response.

All patients were monitored for AEs according to the National Cancer Institute Common Terminology Criteria for Adverse Events, v.4.03 (ref. ^33^). Due to concern for myocarditis based on prior relatlimab studies^1,20^, patients were required to have cardiac troponin testing, in addition to assessment of blood counts, electrolytes, liver and kidney function before each scheduled infusion. All patients underwent baseline tumour staging (either computed tomography or positron-emission tomography-computed tomography of body and magnetic resonance imaging of brain) within 28 days of treatment initiation and again during week 8 for determination of RECIST response. Scans were performed every 3 months in the postoperative setting for up to 2 years after surgery. Core needle biopsy was performed within 28 days of treatment initiation and at weeks 3 and 5 for correlative research. Blood was collected at time of treatment initiation, weeks 3, 5, 9 and then every 12 weeks in the postoperative setting for up to 2 years (Fig. 1). Surgical resection was completed at week 9 per institutional standards and per the guidelines of the INMC^8,10^. Pathologic review of surgical resection specimens was performed by a small group of dermatopathologists who assessed the specimens according to the practices outlined by the INMC^2^. pCR was defined as no viable tumour, near pCR as greater than 0% but less than or equal to 10% viable tumour, pPR as greater than 10% to less than or equal to 50% viable tumour and pNR as greater than 50% viable tumour.

### Study oversight

The study was conducted in accordance with the clinical trial protocol and Good Clinical Practices Guidelines as defined by the International Conference on Harmonization and the Declaration of Helsinki. The study was approved by the institutional review boards of MD Anderson Cancer Center and Memorial Sloan Kettering Cancer Center. All patients provided informed consent for participation in the clinical trial. The study was designed by investigators at MD Anderson Cancer Center and the manuscript was written by the authors in its entirety. Trial monitoring was by the Investigational New Drugs office at MD Anderson Cancer Center. Study drugs were supplied by Bristol-Myers Squibb.

### Statistical analyses

RFS time was computed from surgery date to date of progression/recurrence or death (if died without progression/recurrence). Event-free survival time was computed from start of treatment to date of progression/recurrence or death (if died without progression/recurrence). Patients alive at the last follow-up date who did not experience progression/recurrence were censored. Patients who died without experiencing progression/recurrence were censored. Overall survival time was computed from start of neoadjuvant therapy to last known vital status. Patients alive at the last follow-up date were censored. The Kaplan–Meier method was used to estimate the outcome measures, and group differences were evaluated using the log-rank test. All statistical analyses were performed using SAS v.9.4 for Windows.

### Correlative studies

Blood and tumour were collected at the timepoints shown in Fig. 1. Cells were isolated and prepared from peripheral blood and tumour tissues for flow cytometry and CyTOF analyses as per the specifications below.

#### Isolation and preparation of cells from peripheral blood and tissues

Whole blood was collected in tubes containing sodium heparin (BD Vacutainer), resuspended in phosphate-buffered saline (PBS), layered atop Ficoll (StemCell Technologies) and centrifuged at 800*g* for 25 min. The interface peripheral blood mononuclear cells (PBMCs) were harvested and washed twice with PBS and centrifuged at 500*g* for 10 min. Fresh tumour tissue was dissociated with GentleMACS system (Miltenyi Biotec). PBMC and tumour specimens destined for CyTOF analysis were stained for viability with 5 μmol l^−1^ cisplatin (Fluidigm, now Standard Biotools) in PBS containing 1% bovine serum albumin (BSA) and then washed three times. All specimens were resuspended in AB serum with 10% (vol/vol) dimethyl sulfoxide for storage in liquid nitrogen until downstream assays were performed.

### Flow cytometry staining and analysis

**Extended Data Fig. 5:**
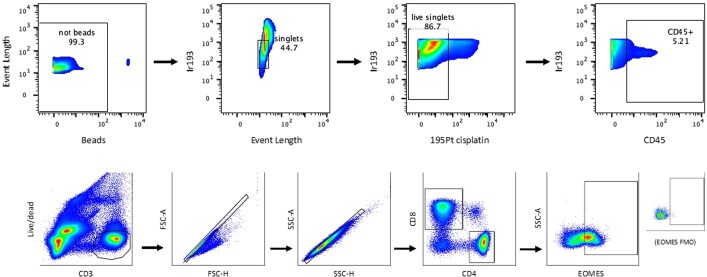
Gating schema for manual analysis of CyTOF data from tumour and blood specimens. **A**) Tumours were mechanically dissociated and cells were stained with immune cell-specific antibodies. Specimens were assayed on the Helios mass cytometer via CyTOF Software. Cytometer data were then prepared for manual and unsupervised analyses via FlowJo software. Major cell populations were identified manually and reported. An example of one patient specimen is shown above for reference. **B**) Gating schema for flow cytometric analysis of blood specimens. Peripheral blood mononuclear cells from patient specimens were stained along with FMO (fluorescence minus one) controls and assayed via a BD LSRFortessa cytometer and BD FACSDiva acquisition software. Data were analysed via FlowJo software as described above. Briefly, live CD3+ singlets were identified and gated into T cell lineages, and those lineages analysed for frequency of each of eight phenotypic markers (BCL6, BLIMP1, CD27, CD28, cMYC, EOMES, ICOS, Ki67) as defined by FMO (fluorescence minus one) specimens. An example of one phenotypic marker (EOMES) in one patient specimen is shown above for reference.

Flow cytometry analysis was performed on PBMCs (see Extended Data Table 3 for antibodies used in flow cytometry). Single-cell suspensions were stained with 16 fluorescent primary antibodies and live/dead dye. Specimens were analysed using the BD LSRFortessa ×20 cytometer and BD FACSDiva acquisition software v.8.0.1 (BD Biosciences), and downstream analyses were performed manually using FlowJo software v.10.5.3 (BD). See Extended Data Fig. 5 for flow cytometry sequential gating/sorting strategies.

### Mass cytometry staining and analysis

CyTOF analyses were performed on tumour specimens as well as PBMCs (see Extended Data Table 4 for antibodies used in CyTOF analysis). Single-cell suspensions were assayed with 41 antibodies, plus Ir DNA-intercalator and cisplatin. Antibodies were either purchased preconjugated from Fluidigm or purchased purified and conjugated in-house using MaxPar X8 Polymer kits (Fluidigm, now Standard Biotools). Briefly, samples were thawed and stained with cell surface antibodies in PBS containing 5% goat serum and 1% BSA for 30 min at 4 °C. Samples were then washed in PBS containing 1% BSA, fixed and permeabilized according to the instructions of the manufacturers using the FoxP3 staining buffer set (eBioscience), before being incubated with intracellular antibodies in permeabilization buffer for 30 min at 4 °C. Samples were washed and incubated in Ir intercalator (Fluidigm, now Standard Biotools) and stored at 4 °C until acquisition, generally within 12 h. Immediately before acquisition, samples were washed and resuspended in water containing EQ 4 element beads (Fluidigm, now Standard Biotools). Samples were acquired on a Helios mass cytometer (Fluidigm, now Standard Biotools).

FCS files were preprocessed in R (R Foundation for Statistical Computing (https://www.R-project.org/)) using a CyTOF package (Premessa, Parker Institute for Cancer Immunotherapy (https://github.com/ParkerICI)) and gated manually in FlowJo (BD). Data were then exported as FCS files for downstream analysis and arcsinh transformed using a coefficient of 5 [x_transformed = arcsinh(x/5)]. To visualize the high-dimensional data in two dimensions, the t-Distributed Stochastic Neighbor Embedding dimension reduction algorithm was applied, using all channels besides those used to manually gate the population of interest (for example, CD45 or CD3). Clustering analysis was performed in R using the FlowSOM and ConsensusClusterPlus packages^34^.

### Graphics and statistics

Graphs were created and statistical analyses performed using GraphPad Prizm v.9.2 (GraphPad Software, LLC).

### Reporting summary

Further information on research design is available in the Nature Research Reporting Summary linked to this article.

## Online content

Any methods, additional references, Nature Research reporting summaries, source data, extended data, supplementary information, acknowledgements, peer review information, details of author contributions and competing interests, and statements of data and code availability are available at 10.1038/s41586-022-05368-8.

### Supplementary information

Supplementary Information This file contains the Protocol.

Reporting Summary

Peer Review File

### Source data

Source Data Fig. 4 and Extended Data Figs. 2 and 3.

## Figures and Tables

**Extended Data Table 1:**
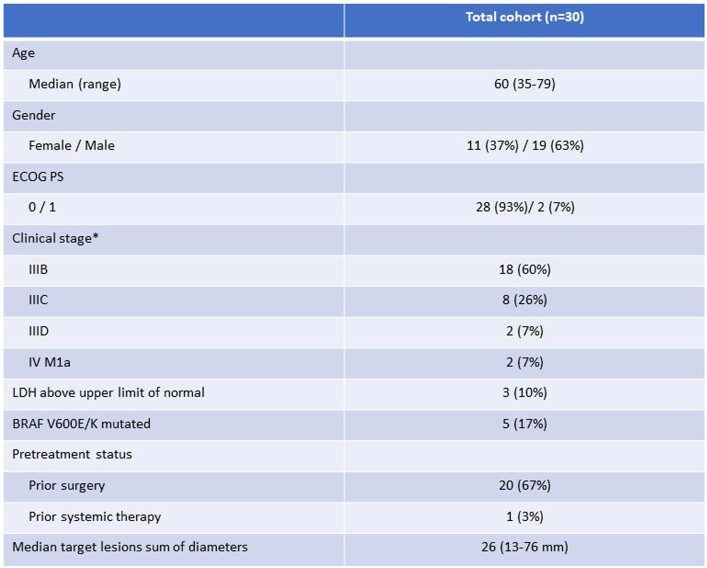
Baseline patient characteristics

**Extended Data Table 2:**
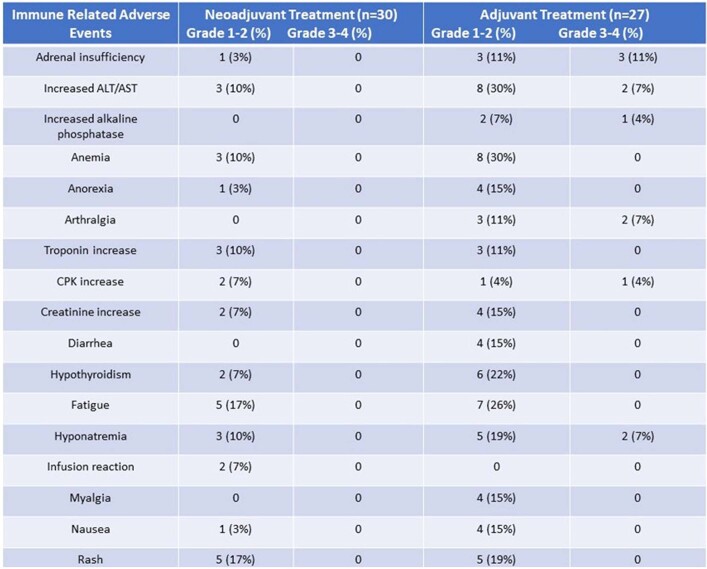
Immune-related adverse events during neoadjuvant and adjuvant therapy

**Extended Data Table 3:**
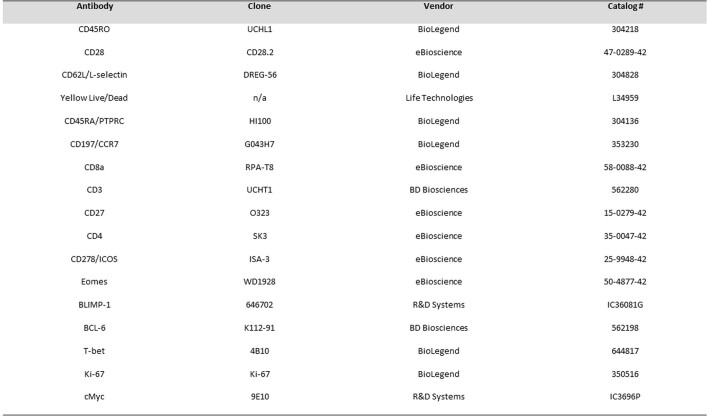
Antibodies for flow cytometry analysis

**Extended Data Table 4:**
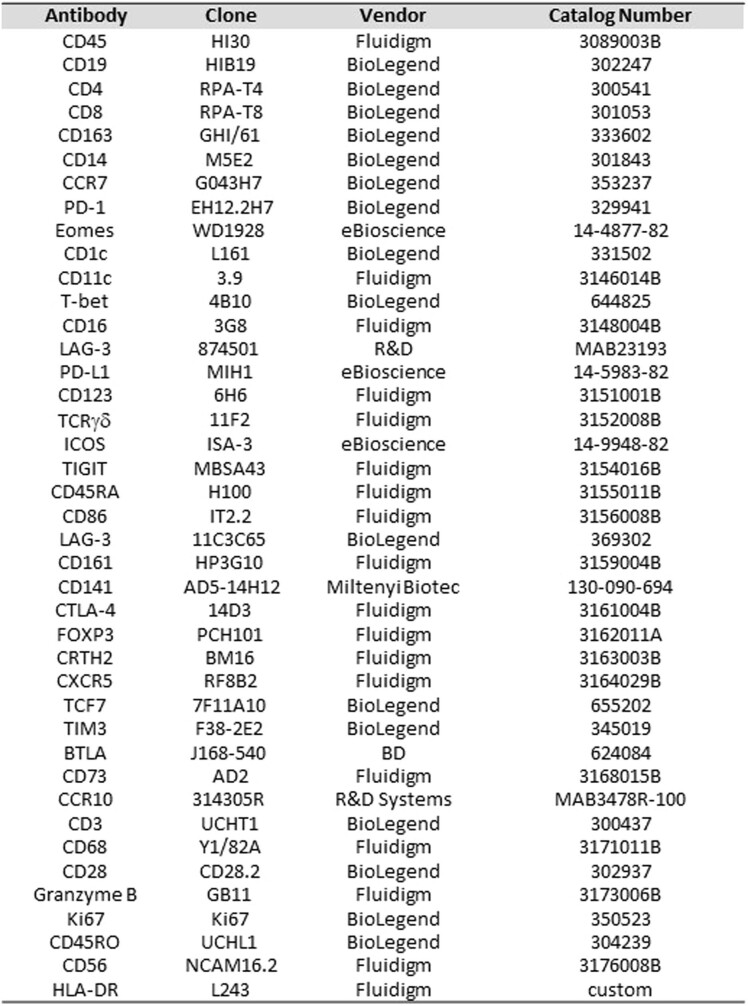
Antibodies for CyTOF analysis

## Data Availability

Data supporting the findings of this study have been provided to *Nature* through direct deposition.

## References

[1] Tawbi HA (2022). Relatlimab and nivolumab versus nivolumab in untreated advanced melanoma. N. Engl. J. Med..

[2] Tetzlaff MT (2018). Pathologic assessment of resection specimens after neoadjuvant therapy for metastatic melanoma. Ann. Oncol..

[3] Gershenwald JE (2017). Melanoma staging: evidence-based changes in the American Joint Committee on Cancer: eighth edition cancer staging manual. CA Cancer J. Clin..

[4] Long GV (2017). Adjuvant dabrafenib plus trametinib in stage III BRAF-mutated melanoma. N. Engl. J. Med..

[5] Eggermont AMMM (2018). Adjuvant pembrolizumab versus placebo in resected stage III melanoma. N. Engl. J. Med..

[6] Weber J (2022). Five-year outcomes with adjuvant nivolumab versus ipilimumab in resected stage IIIB-C or IV melanoma (CheckMate 238). Pig. Cell Mel. Res..

[7] Long, G. V. et al. Adjuvant therapy with nivolumab combined with ipilimumab vs nivolumab alone in patients with resected stage IIIB-D/IV melanoma (CheckMate 915). *Cancer Res.*10.1158/1538-7445.AM2021-CT004 (2021).

[8] Amaria RN (2019). Neoadjuvant systemic therapy in melanoma: recommendations of the International Neoadjuvant Melanoma Consortium. Lancet Oncol..

[9] Menzies AM (2021). Pathological response and survival with neoadjuvant therapy in melanoma: a pooled analysis from the International Neoadjuvant Melanoma Consortium (INMC). Nat. Med..

[10] Van Akkooi ACJ (2022). Neoadjuvant systemic therapy (NAST) in patients with melanoma: surgical considerations by the International Neoadjuvant Melanoma Consortium. Ann. Surg. Oncol..

[11] Cortazar P (2014). Pathological complete response and long-term clinical benefit in breast cancer: the CTNeoBC pooled analysis. Lancet.

[12] Petrelli F (2014). Correlation of pathologic complete response with survival after neoadjuvant chemotherapy in bladder cancer treated with cystectomy: a meta-analysis. Eur. Urol..

[13] Kasi A (2020). Total neoadjuvant chemotherapy vs standard therapy in locally advanced rectal cancer: a systematic review and meta-analysis. JAMA Netw. Open..

[14] Liu J (2016). Improved efficacy of neoadjuvant compared to adjuvant immunotherapy to eradicate metastatic disease. Cancer Disc..

[15] Blank CU (2018). Neoadjuvant versus adjuvant ipilimumab plus nivolumab in macroscopic stage III melanoma. Nat. Med..

[16] Amaria RN (2018). Neoadjuvant immune checkpoint blockade in high-risk resectable melanoma. Nat. Med..

[17] Rozeman EA (2019). Identification of the optimal combination dosing schedule of neoadjuvant ipilimumab plus nivolumab in macroscopic stage III melanoma (OpACIN-neo): a multicenter, phase 2, randomized, controlled trial. Lancet Oncol..

[18] Woo S-R (2012). Immune inhibitory molecules LAG-3 and PD01 synergistically regulate T cell function to promote tumoral immune escape. Cancer Res..

[19] Anderson AC, Joller N, Kuchroo VK (2016). Lag-, Tim-3, and TIGIT co-inhibitory receptors with specialized functions in immune regulation. Immunity..

[20] Ascierto PA (2017). Efficacy of BMS-986016, a monoclonal antibody that targets lymphocyte activation gene-3 (LAG-3), in combination with nivolumab in pts with melanoma who progressed during prior anti-PD-1/PD-L1 therapy (mel prior IO) in all-comer and biomarker-enriched populations. Ann. Oncol..

[21] Huang AC (2019). A single dose of neoadjuvant PD-1 blockade predicts clinical outcomes in resectable melanoma. Nat. Med..

[22] Amaria RN (2018). Neoadjuvant plus adjuvant dabrafenib and trametinib versus standard of care in patients with high-risk, surgically resectable melanoma: a single-centre, open-label, randomised, phase 2 trial. Lancet Oncol..

[23] Long GV (2019). Neoadjuvant dabrafenib combined with trametinib for resectable, stage IIIB-C, BRAF(V600) mutation-positive melanoma (NeoCombi): a single-arm, open-label, single-centre, phase 2 trial. Lancet Oncol..

[24] Forde PM (2018). Neoadjuvant PD-1 blockade in resectable lung cancer. N. Engl. J. Med..

[25] Topalian SL (2020). Neoadjuvant nivolumab for patients with resectable merkel cell carcinoma in the CheckMate 358 trial. J. Clin. Oncol..

[26] Vos JL (2021). Neoadjuvant immunotherapy with nivolumab and ipilimumab induces a major pathologic response in patients with head and neck squamous cell carcinoma. Nat. Commun..

[27] Riaz N (2017). Tumor and microenvironment evolution during immunotherapy with nivolumab. Cell.

[28] Rozeman EA (2021). Survival and biomarker analyses from OpACIN-neo and OpACIN neoadjuvant immunotherapy trials in stage III melanoma. Nat. Med..

[29] Jordan KR (2013). Myeloid-derived suppressor cells are associated with disease progression and decreased overall survival in advanced-stage melanoma patients. Cancer Immunol. Immunother..

[30] Llao-Cid L (2021). EOMES is essential for antitumor activity of CD8+ T cells in chronic lymphocytic leukemia. Leukemia.

[31] Helmink BA (2020). B cells and tertiary lymphoid structures promote immunotherapy response. Nature.

[32] Eisenhauer EA (2009). New response evaluation criteria in solid tumours: revised RECIST guideline (version 1.1). Eur. J. Cancer..

[33] U.S. Department of Health and Human Services,National Institutes of Health & National Cancer Institute. Common Terminology Criteria for Adverse Events (CTCAE) v.4.03. https://evs.nci.nih.gov/ftp1/CTCAE/CTCAE_4.03/CTCAE_4.03_2010-06-14_QuickReference_8.5x11.pdf (2010).

[34] Nowicka M (2017). CyTOF workflow: differential discovery in high-throughput high-dimensional cytometry datasets. F1000Res..

