# Author Correction: Neoadjuvant relatlimab and nivolumab in resectable melanoma

**DOI:** 10.1038/s41586-023-05892-1

**Published:** 2023-03-09

**Authors:** Rodabe N. Amaria, Michael Postow, Elizabeth M. Burton, Michael T. Tetzlaff, Merrick I. Ross, Carlos Torres-Cabala, Isabella C. Glitza, Fei Duan, Denái R. Milton, Klaus Busam, Lauren Simpson, Jennifer L. McQuade, Michael K. Wong, Jeffrey E. Gershenwald, Jeffrey E. Lee, Ryan P. Goepfert, Emily Z. Keung, Sarah B. Fisher, Allison Betof-Warner, Alexander N. Shoushtari, Margaret Callahan, Daniel Coit, Edmund K. Bartlett, Danielle Bello, Parisa Momtaz, Courtney Nicholas, Aidi Gu, Xuejun Zhang, Brinda Rao Korivi, Madhavi Patnana, Sapna P. Patel, Adi Diab, Anthony Lucci, Victor G. Prieto, Michael A. Davies, James P. Allison, Padmanee Sharma, Jennifer A. Wargo, Charlotte Ariyan, Hussein A. Tawbi

**Affiliations:** 1 Department of Melanoma Medical Oncology, The University of Texas MD Anderson Cancer Center, Houston, TX USA; 2 Department of Medicine, Memorial Sloan Kettering Cancer Center and Weill Cornell Medical College, New York, NY USA; 3 Department of Pathology, The University of California San Francisco, San Francisco, CA USA; 4 Department of Surgical Oncology, The University of Texas MD Anderson Cancer Center, Houston, TX US; 5 Department of Pathology, The University of Texas MD Anderson Cancer Center, Houston, TX USA; 6 Department of Immunology, The University of Texas MD Anderson Cancer Center, Houston, TX USA; 7 Department of Biostatistics, The University of Texas MD Anderson Cancer Center, Houston, TX USA; 8 Department of Pathology, Memorial Sloan Kettering Cancer Center, New York, NY USA; 9 Department of Head and Neck Surgery, The University of Texas MD Anderson Cancer Center, Houston, TX USA; 10 Department of Surgical Oncology, Memorial Sloan Kettering Cancer Center, New York, NY USA; 11 Department of Radiology, The University of Texas MD Anderson Cancer Center, Houston, TX USA

**Keywords:** Melanoma, Cancer immunotherapy

Correction to: *Nature* 10.1038/s41586-022-05368-8 Published online 26 October 2022

In the version of this article initially published, the surname of author Michael T. Tetzlaff was misspelled as ‘Tezlaff.’ In addition, the Acknowledgements section omitted mention of grant P50CA221703 from the National Cancer Institute of the National Institutes of Health, which supported the work. The errors have been corrected in the HTML and PDF versions of the article.

