# Supplementary material for: Neoadjuvant relatlimab and nivolumab in resectable melanoma
Source: Nature. 2022 Oct 26;611(7934):155–60. doi: 10.1038/s41586-022-05368-8 (PMC9607737; doi:10.1038/s41586-022-05368-8)
Supplement: Supplementary file 1 — This file contains the Protocol. [file 41586_2022_5368_MOESM1_ESM.pdf]

nature portfolio

https://doi.org/10.1038/s41586-022-05368-8

Supplementary information

Neoadjuvant relatlimab and nivolumab in resectable melanoma

In the format provided by the authors and unedited

MD Anderson IND Sponsor Cover Sheet

**Protocol ID**	2015-0041
**Protocol Title**	A Multicenter Study with Neoadjuvant and Adjuvant Checkpoint Blockade in Patients with Clinical Stage III or Oligometastatic Stage IV Melanoma
**Protocol Phase**	II
**Protocol Version**	12
**Version Date**	11/19/2020
**Protocol PI**	Rodabe N. Amaria, MD
**Department**	Melanoma Medical Oncology
**IND Sponsor**	MD Anderson Cancer Center
**IND #**	127886

CONFIDENTIAL

This is a University of Texas MD Anderson Cancer Center document that contains confidential information. It is intended solely for the recipient clinical investigator(s) and must not be disclosed to any other party. This material may be used only for evaluating or conducting clinical investigations; any other proposed use requires written consent from University of Texas MD Anderson Cancer Center.

PROTOCOL

**TITLE:**	A Multicenter Study with Neoadjuvant and Adjuvant Checkpoint Blockade in Patients with Clinical Stage III or Oligometastatic Stage IV Melanoma
**STUDY NUMBER:**	MDACC: 2015-0041; BMS: CA209-291
**VERSION NUMBER:**	12
**PROTOCOL DATE:**	19 NOVEMBER 2020
**AMENDMENT:**	Amendment 11
**TEST PRODUCTS:**	Nivolumab<br>Ipilimumab<br>Relatlimab
**SPONSOR:**	University of Texas M.D. Anderson Cancer Center
**PRINCIPAL INVESTIGATOR:**	Rodabe N. Amaria, MD<br>University of Texas M.D. Anderson Cancer Center<br>1515 Holcombe Boulevard<br>Houston, Texas 77030<br>(713) 792-2921<br>RNAmaria@mdanderson.org
**CO-INVESTIGATOR:**	Jennifer A. Wargo, MD (MDACC)
**PRINCIPAL INVESTIGATOR (MSKCC):**	Michael A. Postow, MD<br>Memorial Sloan Kettering Cancer Center<br>300 East 66th Street<br>New York, New York 10065<br>Phone: (646) 888-4589<br>Fax: (646) 888-4253<br>Email: PostowM@mskcc.org
**IND NUMBER:**	127,886
**SUPPORT PROVIDED BY:**	Bristol-Myers Squibb (BMS)

Protocol Amendments

Protocol Version and Date	Amendment Number	Approval Date
Version 1 – 1 June 2015	Initial	23 April 2015
Version 2 – 18 August 2015	Amendment 1	30 August 2015
Version 3 – 19 November 2015	Amendment 2	14 December 2015
Version 4 – 9 May 2016	Amendment 3	15 June 2016
Version 5 – 20 June 2016	Amendment 4	18 July 2016
Version 6 – 9 August 2016	Amendment 5	29 September 2016
Version 7 – 19 April 2017	Amendment 6	12 June 2017
Version 8 – 29 May 2018	Amendment 7	27 July 2018
Version 9 – 27 Septmber 2018	Amendment 8	05 October 2018
Version 10 – 28 October 2019	Amendment 9	25 November 2019
Version 11 – 01 September 2020	Amendment 10	11 September 2020

Table of Contents

1 Trial Summary....7
1.1 Trial Summary Table....7
2 Background & Rationale....11
2.1 Introduction....11
2.2 Ipilimumab....11
2.3 Nivolumab....13
2.4 Relatlimab (anti-LAG-3)....15
2.5 Safety of Combination Checkpoint Blockade....16
2.6 Neoadjuvant Approach....18
3 Objectives & Endpoints....19
3.1 Primary Objective....19
3.2 Secondary Objectives....19
3.3 Exploratory Objectives....20
4 Ethical Considerations....20
4.1 Good Clinical Practice....20
4.2 Institutional Review Board/Independent Ethics Committee....21
4.3 Informed Consent....21
5 Investigational Plan....22
5.1 Study Design....22
5.2 Study Assessments....24
5.3 Treatment Assignment....25
5.4 Eligibility Criteria....25
5.4.1 Inclusion Criteria....25
5.4.2 Exclusion Criteria....28
6 Treatment Plan....31
6.1 Investigational Product....31
6.2 Handling and Dispensing....31
6.3 Destruction....31
6.4 Product Descriptions....32
6.4.1 Nivolumab (BMS-936558)....32
6.4.2 Ipilimumab....32
6.4.3 Relatlimab (BMS-986016)....33
6.5 Dose Calculations and Administration....34
6.5.1 Dose Modifications....35
6.5.2 Dose Delay Criteria....35

6.5.3 Study-Drug Discontinuation Criteria ... 37
6.5.4 Treatment of Immunotherapy-Related Infusion Reactions ... 38
6.6 Permanent Discontinuation from Study Treatment and Subject Completion Criteria 39
6.6.1 Subject Completion Criteria ... 39
6.6.2 Permanent Discontinuation from Study Treatment ... 39
6.7 Prohibited Medications and Non-Drug Therapies ... 40
7 Schedule of Assessments ... 41
7.1 Study Procedure Tables ... 41
7.2 Critical Baseline Assessments ... 46
7.3 Safety Evaluations on Therapy ... 46
7.3.1 Physical Exam/ECOG/Vital Signs/Concomitant Medications ... 46
7.3.2 Adverse Event Assessment ... 47
7.3.3 Laboratory Assessments ... 47
7.4 Biospecimen Collections ... 48
7.4.1 Peripheral Blood and Serum Samples ... 48
7.4.2 Tumor Biopsies and Surgical Specimen ... 48
7.5 Disease Assessments ... 49
7.5.1 Pathologic Assessments ... 49
7.5.2 Imaging Assessments ... 50
7.5.3 Translational Research/Biomarker Analysis ... 50
8 Safety and Reporting ... 52
8.1 Sponsor SAE Reporting ... 54
8.2 Non-Serious Adverse Event Collection and Reporting ... 57
8.3 Laboratory Test Abnormalities ... 57
8.4 Pregnancy ... 58
8.5 Overdose ... 58
8.6 Other Safety Considerations ... 58
9 Statistical Considerations ... 58
9.1 Sample Size and Power ... 58
Arm A and Arm B ... 58
Arm C ... 59
9.2 Study Populations ... 59
9.2.1 Safety Population ... 59
9.3 Stopping Rule ... 59
9.4 Patient Characteristics ... 62
9.5 Primary Efficacy Measure ... 62

9.6 Secondary Efficacy Measures ... 63
9.6.1 Immunologic Response ... 63
9.6.2 Objective Response ... 63
9.6.3 Recurrence-Free and Overall Survival ... 63
9.7 Exploratory Efficacy Measures ... 63
9.8 Safety Measures ... 63
10 Study Management ... 64
10.1 Data Management ... 64
10.1.1 Enrollment ... 64
10.1.2 Data Entry and Maintenance ... 64
10.1.3 Records Retention ... 64
11 References ... 65
12 Appendices ... 71

List of Figures

Figures 1a and 1b Study Design Schema ... 23

List of Tables

Table 1 Eligibility Guidelines of Organ and Marrow Function ... 26
Table 2 Product Descriptions ... 34
Table 3 Study Procedure Table for Patients Randomized to Arm A (neoadjuvant and adjuvant nivolumab) ... 41
Table 4 Study Procedure Table for Patients Randomized to Arm B (neoadjuvant ipilimumab and nivolumab, adjuvant nivolumab) ... 42
Table 5 Study Procedure Table for Patients Enrolled to Arm C (neoadjuvant and adjuvant nivolumab and relatlimab) ... 43
Table 6 Adjuvant Therapy Study Procedure Table for Arm A and Arm B ... 44
Table 7 Adjuvant Therapy Study Procedure Table for Arm C ... 45
Table 8 Required Laboratory Testing ... 47
Table 9 Stopping Boundaries for Arm A and Arm B ... 60
Table 10 Operating Characteristics for Arm A and Arm B ... 60
Table 11 Stopping Criteria - Toxicity for Arm C ... 61
Table 12 Stopping Criteria - Futility for Arm C ... 61
Table 13 Operating Characteristics for Arm C ... 62

Appendices

Appendix 1 Melanoma of the Skin Aging 71
Appendix 2 RECIST 1.1 Criteria 72
Appendix 3 Guidance on Contraception 77
Appendix 4: GI Adverse Event Management Algorithm 81
Appendix 5: Renal Adverse Event Management Algorithm 82
Appendix 6: Pulmonary Adverse Event Management Algorithm..................................83
Appendix 7: Hepatic Adverse Event Management Algorithm.......................................84
Appendix 8: Endocrinopathy Adverse Event Management Algorithm 85
Appendix 9: Skin Adverse Event Management Algorithm..86
Appendix 10: Neurological Adverse Event Management Algorithm 87

1 Trial Summary

1.1 Trial Summary Table

**Investigational Products, Dose, Administration, Duration of Treatment:**	• Arm A: Nivolumab (BMS-936558) monotherapy administered IV over 30 minutes at 3 mg/kg every 2 weeks for 4 doses (administered week 1, 3, 5, 7) prior to surgical excision. Postoperative nivolumab administered IV over 30 minutes at 3 mg/kg every 2 weeks for 6 months (Closed to New Patient Enrollment (CNPE) as of 11 July 2017).<br><br>• Arm B: Nivolumab administered IV over 60 minutes at 1 mg/kg combined with ipilimumab administered IV over 90 minutes at 3 mg/kg every 3 weeks for three doses (week 1, 4, 7) prior to surgical excision. Postoperative nivolumab administered IV over 30 minutes at 3 mg/kg every 2 weeks for 6 months (Closed to New Patient Enrollment (CNPE) as of 11 July 2017).<br><br>• Arm C: Relatlimab 160 mg IV and Nivolumab 480mg IV will be administered together over approximately 60 minutes every 28 days for 2 doses in the neoadjuvant setting. Post-operatively, Relatlimab 160 mg IV together with Nivolumab 480 mg IV will be administered every 28 days for an additional 10 doses (Open to Enrollment).
**Research Hypothesis:**	Treatment with neoadjuvant immunotherapy will produce complete responses in high-risk resectable melanoma.
**Objectives:**	<u>Primary:</u><br><br>Arm A and Arm B<br><br>• To assess the pathologic response of nivolumab monotherapy and nivolumab and ipilimumab dual therapy administered in the neoadjuvant setting in patients with high- risk resectable melanoma. Pathologic response will be assessed by percent viable tumor cells, percent tumor necrosis, presence of fibrosis and melanoma proliferation as assessed by phosphohistone H3 from baseline, to on-treatment and surgical specimens.<br><br>Arm C<br><br>• To assess the pathologic response rate of combination relatlimab with nivolumab in the neoadjuvant setting in patients with high- risk resectable Stage IIIB/C or oligometastatic Stage IV melanoma. Pathologic response will be assessed by percent viable tumor cells, percent tumor necrosis, presence of fibrosis and melanoma proliferation as assessed by phosphohistone H3 from baseline, to on-treatment and surgical specimens.<br><br>•<br><br><u>Secondary:</u><br><br>Arm A and Arm B<br><br>• To assess the immunologic response of neoadjuvant nivolumab monotherapy and neoadjuvant nivolumab and ipilimumab dual therapy in patients with high-risk resectable melanoma. Immunologic response will be

	determined by change in T cell infiltrate from baseline to on-treatment and surgical specimens in response to therapy<br><br>• To assess the objective response rate (ORR) of nivolumab monotherapy and nivolumab and ipilimumab dual therapy administered in the neoadjuvant setting as assessed by imaging (RECIST 1.1 criteria) in patients with high-risk resectable melanoma<br><br>• To assess the 12-month recurrence-free survival (RFS) and overall survival (OS) of patients with high-risk resectable melanoma treated with neoadjuvant nivolumab monotherapy or nivolumab and ipilimumab dual therapy followed by adjuvant nivolumab<br><br>• To evaluate the safety of nivolumab monotherapy and dual ipilimumab and nivolumab in the neoadjuvant setting and peri-operatively as well as assess the safety of adjuvant nivolumab.<br><br>Arm C<br><br>• To evaluate the safety and feasibility of relatlimab with nivolumab delivered in the neoadjuvant setting<br><br>• To assess the objective response rate (ORR) of relatlimab with nivolumab administered in the neoadjuvant setting as assessed by imaging (RECIST 1.1 criteria) in patients with high-risk resectable melanoma<br><br>• To assess the 12-month recurrence-free survival (RFS) and overall survival (OS) of patients with high-risk resectable melanoma treated with neoadjuvant and adjuvant relatlimab with nivolumab<br><br>• To evaluate immunologic and molecular mechanisms of response and resistance to relatlimab with nivolumab.<br><br><u>Exploratory:</u><br><br>• Identification of immunologic and genomic markers correlating with clinical and pathological response and resistance.
**Study Design:**	This is a Phase 2, randomized non-comparative study of neoadjuvant nivolumab monotherapy or nivolumab combined with ipilimumab followed by adjuvant nivolumab or nivolumab combined with relatlimab in adult (≥18 years) subjects with resectable, high-risk melanoma. Patients with prior adjuvant therapy including use of prior interferon alpha, pegylated interferon or vaccine) will be eligible after a 28 day washout period. Subjects must have stage IIIB/IIIC or stage IV resectable, oligometastatic (less than or equal to 3 sites of disease, excluding bone and CNS) melanoma, as per the American Joint Committee on Cancer (AJCC) stagirng system. Subjects will be treated with one of the following:<br><br>• Arm A: nivolumab 3 mg/kg (+/- 7 days) IV Q2W (4 doses) prior to surgical excision (CNPE).

- Arm B: nivolumab 1 mg/kg (+/- 7 days) IV combined with ipilimumab 3 mg/kg IV Q3W (3 doses) prior to surgical excision (CNPE).

A new treatment (Arm C) has been incorporated in Amendment 7 of this protocol (BMS: CA209-291 and MDACC: 2015-0041). Patients will receive relatlimab (anti-LAG-3) in combination with Nivolumab for 2 doses in the neoadjuvant setting. An additional 10 doses of relatlimab with nivolumab will be administered to the patients in the adjuvant setting.

- Arm C: nivolumab 480 mg combined with relatlimab 160 mg every 28 days for 2 doses prior to surgical excision, then 10 doses of nivolumab 480 mg with relatlimab 160mg, post-operatively.

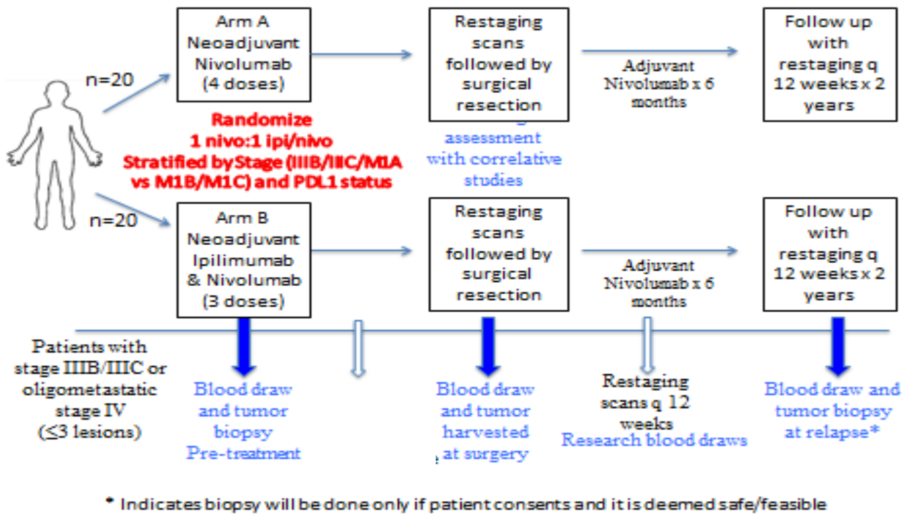

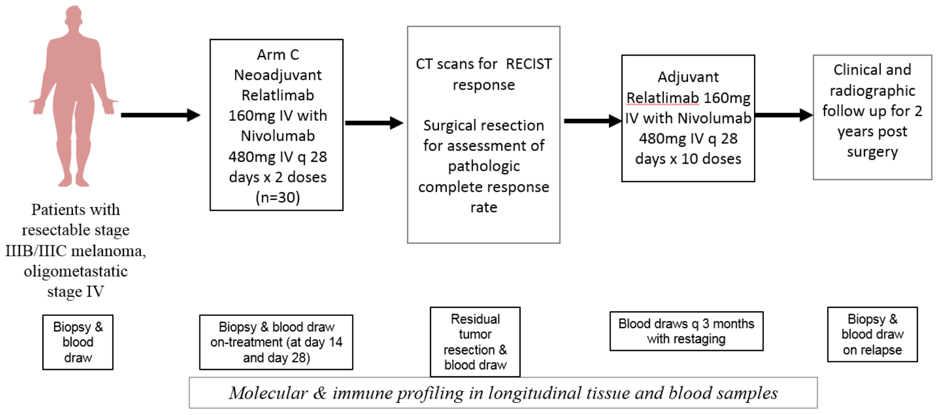

Study Population:

Key Inclusion Criteria:

- ECOG PS 0 or 1.
- Histologically confirmed stage IIIB/IIIC (resectable) or oligometastatic (≤3), resectable stage IV melanoma, as per AJCC staging system.
- Patients must have at least one tumor amenable to serial biopsy in clinic or be willing to undergo serial biopsies through image-guided

	procedures. Baseline, on-treatment and surgical samples will be collected for analysis.<br>• Tumor tissue must be assessed for PD-L1 expression via IHC.<br><br>Key Exclusion Criteria:<br>• Brain metastases, leptomeningeal or bone metastases.<br>• Subjects with active, known or suspected autoimmune disease. Subjects with vitiligo, type I diabetes mellitus, residual hypothyroidism due to autoimmune condition only requiring hormone replacement, psoriasis not requiring systemic treatment, or conditions not expected to recur in the absence of an external trigger are permitted to enroll<br>• Subjects with a condition requiring systemic treatment with either corticosteroids (> 10 mg daily prednisone equivalents) or other immunosuppressive medications within 14 days of study drug administration. Inhaled or topical steroids, and adrenal replacement doses > 10 mg daily prednisone equivalents are permitted in the absence of active autoimmune disease.<br>• Prior treatment with an anti-PD-1, anti-PD-L1, anti-PD-L2, or anti-CTLA-4 antibody
**Study Assessments:**	Pathologic response to neoadjuvant therapy is primary endpoint of the study. This will be assessed by percentage of viable tumor cells, percent tumor necrosis, amount of fibrosis and proliferation by phosphohistone H3. These factors will be assessed in a continuum over serial time-points including baseline, prior to dose two (and potentially dose 3 when feasible) and surgical resection samples.<br><br>Secondary objectives include immunologic response which will be assessed by change in immune infiltrate in tumor from baseline to on-treatment and surgical resection specimens. Additional secondary objectives include objective response rate to neoadjuvant therapy as assessed by RECIST 1.1 criteria. Recurrence free and overall survival will also be determined by long-term follow up of these patients. Safety assessments will also be made by CTCAE version 4.0 criteria.
**Statistical Considerations:**	Sample Size:<br><br>A total of 53 patients will be enrolled on this study. Prior to Amendment 7, patients were stratified by stage of disease (IIIB/IIIC/M1A vs. M1B/M1C) and PD-L1 tumor status and then randomized in a 1:1 ratio for Arms A and B. Twelve patients were randomized to Arm A, and 11 patients were randomized to Arm B. After approval of Amendment 7, thirty patients will be enrolled to Arm C without randomization, and Arms A and B will be closed to Enrollment. Patient enrollment is expected at a rate of 2-4 patients per month.

2 Background & Rationale

2.1 Introduction

Melanoma accounts for less than 2% of skin cancer cases but is responsible for the large majority of skin cancer deaths.[1] Moreover, the incidence of melanoma has been increasing for the past 30 years worldwide. In the United States alone, the incidence has increased by an average of 4% per year since the early 1970s. [2]

Surgery has long been the mainstay of treating resectable melanoma. When there is not obvious distant metastatic disease, the initial treatment consists of widely excising the melanoma lesion and, often, sampling or removal of the draining lymph nodes. In its earliest stage (stage I), surgery alone provides good long-term survival, with over 90% of patients alive ten years after treatment. However, as the stage increases, the chance of recurrent or metastatic disease increases substantially. Less than half of patients with clinically detectable lymph node involvement and fewer than 15% of patients with distant disease will survive ten years. [3]

In the past, systemic options to decrease the risk of recurrence in patients with high-risk, resectable disease and to treat patients with metastatic disease have been limited. Historically, the best available treatment has been interferon alpha-2b therapy.[4] Its use in treating patients with resected stage II/III disease has not consistently improved overall survival in multiple clinical trials and generally has poor tolerance secondary to significant treatment related toxicities. [5-10] Similarly, in the metastatic setting, high dose interleukin-2 produces a low overall response rate (16%), and again, with serious toxicity, so that it is given only to selected patients.[4 11]

More recently, systemic therapy for melanoma has undergone a seismic shift with the advent of two new drug classes. The first class is small molecule kinase inhibitors to BRAF (dabrafenib and vemurafenib) and MEK (trametinib), which have demonstrated a survival benefit and are FDA approved in BRAF V600 mutated metastatic melanoma. [12-14] The second new class of drugs is immune checkpoint inhibitors. Ipilimumab, a monoclonal antibody (mAb) antagonist to CTLA-4, has improved survival and is FDA approved in both treatment naïve and pretreated metastatic melanoma.[15,16] A second immune checkpoint mAb, nivolumab, which targets PD-1, demonstrated promising initial results and the phase III study was stopped early due to clear benefit in overall survival associated with PD-1 blockade and is anticipated to be FDA approved by the end of 2014.[17,18] The role of these new therapeutic options in the neoadjuvant and adjuvant settings remains to be fully elucidated and is an active area of investigation. The aim of this trial is to further clarify the benefit of PD-1 blockade (nivolumab) alone or in combination with CTLA-4 blockade (ipilimumab) given to patients with resectable, high- risk melanoma.

2.2 Ipilimumab

Cytotoxic T-lymphocyte-associated antigen-4 (CTLA-4, CD152) was the first recognized and is the best characterized inhibitory immune checkpoint molecule. [19,20] CTLA-4 is the only checkpoint currently targeted with an FDA-approved therapeutic drug, ipilimumab. CTLA-4 functions as a negative counterpart to CD28, the required costimulatulatory signal for the

activation and expansion of T-cells. CTLA-4 is an inhibitory checkpoint molecule expressed on activated T-cells and constitutively expressed on regulatory T- cells (Treg).[20] After TCR-antigen mediated activation of T-lymphocytes, expression of CTLA-4 on the cell membrane increases dramatically. CLTA-4 appears to suppress immune activation through multiple pathways and the relative importance of each in overall immune homeostasis and in disease-related autoimmunity and immune suppression is not clear.[21]

Preclinical work with anti-CTLA-4 mAbs supported the notion of an inhibitory role for CTLA-4.[22,23] Soon thereafter, work with anti-CTLA-4 blocking mAbs for therapeutic purposes proceeded. Initial animals studies confirmed that the mAbs could indeed augment immune response to peptide antigens.[23] Not long after this, CTLA-4 mAbs were used to experimentally overcome antitumor immune tolerance.[24] Preclinical data suggests and it stands to reason, that less immunogenic tumors are less likely to respond to CTLA-4 mAb blockade alone.[25] Given the known immunogenicity of melanoma, initial clinical development was focused in this malignancy.

Initial phase I and II studies of ipilimumab monotherapy demonstrated encouraging results.[26-27] The first phase III study of ipilimumab, sponsored by Bristol-Meyers Squibb, began enrolling patients in September 2004. The trial enrolled 676 HLA-A*0201^{+} patients with pretreated, unresectable stage III or IV melanoma. The patients were randomized 3:1:1 to receive either ipilimumab with gp100 peptide vaccine, ipilimumab alone, or gp100 alone. Ipilimumab was dosed at 3 mg/kg every three weeks for four doses. Patients were not routinely offered maintenance therapy, however, those who progressed after responding to therapy or who had stable disease after twelve weeks were allowed “reinduction” therapy. The primary endpoint of the trial was overall survival (OS). The trial demonstrated an OS benefit in all patients who received ipilimumab (median OS: 10.0 months for ipilimumab with gp100, 10.0 months for ipilimumab alone, and 6.4 months for gp100 alone; $p<0.003$). There was no difference in survival in patients who received ipilimumab with gp100 and those who received ipilimumab alone. There were four cases of complete responses and multiple cases of long-term disease control in patients who received ipilimumab in the initial report.[28] Importantly, long-term follow-up has demonstrated that approximately 20% of patients achieve a long-term survival benefit from ipilimumab treatment, a finding that has been since replicated in multiple studies with ipilimumab in melanoma.[29] Approximately 60% of patients treated with ipilimumab experienced an immune-related adverse event (irAE), with the rates of serious irAEs (≥ grade 3) of 10% to 15% in the ipilimumab groups.[15] Of the 31 patients who met criteria for and received “reinduction” therapy (progression after complete or partial response or stable disease), 19% achieved a complete or partial response and 68% achieved disease control with similar toxicity to the original induction therapy.[30] Based on this study, ipilimumab achieved FDA approval at a dose of 3.0mg/kg to treat unresectable stage III and stage IV melanoma.

Ipilimumab was further tested in treatment naïve melanoma patients in a randomized phase III trial evaluating ipilimumab with dacarbazine versus dacarbazine alone.[27] A dose of 10 mg/kg of ipilimumab was used in combination with dacarbazine. Five- hundred-two patients were enrolled and randomized 1:1 to receive ipilimumab plus dacarbazine every three weeks for four doses followed by dacarbazine every three weeks until week 22 or placebo plus dacarbazine at the same schedule. Patients with stable disease or Response Evaluation Criteria in Solid Tumor (RECIST) objective responses were able to receive maintenance

ipilimumab or placebo every 12 weeks. Of note, based on emerging consensus from previous work with CTLA-4 blockade and other immunotherapy, the primary endpoint was changed, with FDA approval, from progression-free survival to OS prior to unblinding of the treatment groups or data analysis. [31,32] Ultimately, the trial showed that patients who received the combination of ipilimumab with dacarbazine survived longer (11.2. months) compared to dacarbazine alone (9.2 months, p<0.001). The difference became more pronounced with time, as the combination arm had 20.8% of patients alive at three years compared to 12.2% in the chemotherapy only arm. The toxicities were greater in the combination arm and also greater than in many of the previous ipilimumab studies (56% ≥ grade 3), likely secondary to the higher dose (10 mg/kg) of ipilimumab used as well as the addition of chemotherapy. However, there were no treatment-related deaths reported.[16] Based on the results of this study, the combination of ipilimumab and dacarbazine is approved for first-line therapy for unresectable melanoma.

Ipilimumab is being evaluated as an adjuvant therapy in melanoma in two trials. ECOG 1609 is an on-going randomized phase III trial comparing adjuvant ipilimumab 3mg/kg, ipilimumab 10mg/kg, and interferon alpha-2b therapy in patients with resected high-risk stage III or stage IV melanoma.[33] The EORTC 18071 phase III trial is a double-blind, randomized trial that has enrolled 951 patients with stage III melanoma to receive ipilimumab 10mg/kg or placebo every three weeks for four doses then every three months for up to three years until recurrence or unacceptable toxicity. Fifty-two percent of patients discontinued treatment due to adverse events. The most common grade III/IV immune-related toxicities in the ipilimumab and placebo arms were gastrointestinal (15.9% vs 0.8%), hepatic (10.6% vs 0.2%) and endocrine (8.5% vs 0%). Five (1.1%) died secondary to treatment related toxicity. The 3-year relapse-free survival rate was 46.5% in the ipilimumab arm, compared to 34.8% in the placebo arm (p=0.001). With a median follow up of 2.7 years, ipilimumab improved the relapse-free survival by a hazard ratio of 0.75.[34]

2.3 Nivolumab

Programmed death 1 (PD-1) is a more recently discovered immune checkpoint receptor that has generated considerable excitement based on favorable preclinical profiling and initial clinical results. Like CLTA-4, PD-1 is a transmembrane protein expressed on effector immune cells.[35] Also like CLTA-4, expression of PD-1 is inducibly expressed with lymphocyte activation, although it is expressed more broadly than CTLA-4 as it is also found on activated B lymphocytes and natural killer cells. [36-38] PD-1 is bound principally by programmed death ligand 1 (PD-L1, B7-H1) but also, to a lesser degree, by programmed death ligand 2 (PD-L2, B7-DC).[39]

The PD-1 receptor pathway is an important negative regulator of the immune system. The PD-1 pathway appears to also have a primary role in cancer tolerance and immune escape. Higher expression of PD-L1 on tumor cells is associated with a worse prognosis, more aggressive features, and/or resistance to immunotherapy in the large majority of cancers that it has been characterized in. [40-48] Furthermore, preclinical data demonstrates that increasing tumor expression of PD-L1 makes it less susceptible to immunotherapy, while blocking it increases its vulnerability to immune-mediated destruction. [49-52]

Based on promising preclinical therapeutic results, PD-1 blocking mAbs have proceeded to human clinical trials. [49,50,52-54] Nivolumab (MDX-1106, BMS-936558, Bristol-Myers Squibb,

New York, NY) is a fully humanized IgG4 monocolonal antibody that binds to PD-1, blocking its binding site. It was initially tested in a phase I, dose escalation trial in 296 patients with heavily pretreated advanced melanoma (n=104), colorectal cancer (n=19), CRPC (n=17), NSCLC (n=122), and renal cell carcinoma (n=34). Nivolumab was given at 0.3, 1, 3, or 10 mg/kg in six patient cohorts followed by expansion cohorts at 10mg/kg. Patients were initially given a single dose and allowed additional doses if they demonstrated clinical benefit, however, the trial transitioned into a phase Ib where patients were dosed every two weeks and reassessed every eight weeks. Treatment was continued for up to 96 weeks or until disease progression or complete response. Treatment with nivolumab was better tolerated overall than treatment with CTLA-4 blocking antibodies with no maximum tolerated dose achieved. Only 14% experienced serious (≥ grade 3) drug toxicity and only 5% had to discontinue therapy secondary to this.

Nivolumab treatment demonstrated substantial antitumor effect, with partial or complete responses (by RECIST criteria) observed in patients with melanoma, NSCLC, and renal cell carcinoma but not colorectal cancer or CRPC. Responses were observed across various doses at rates of 19 to 41% in melanoma, 6 to 32% in NSCLC, and 24 to 31% in renal cell carcinoma. In addition, disease stability and mixed response were observed in a substantial portion of patients. In melanoma patients two and three year OS were 48% and 41%, respectively and the median duration of response in patients with an objective response was an impressive 22.9 months.[55] Further analysis of PD-L1 expression from 61 patients who had pretreatment specimens available demonstrated an objective response in 36% of tumors expressing PD-L1 and none in PD-L1-negative tumors.[17]

Nivolumab monotherapy has been extensively studied in a number of tumor types including non-small cell lung cancer (NSCLC), melanoma, renal cell carcinoma (RCC), and colorectal cancer (CRC) with body weight normalized dosing (mg/kg). Nivolumab pharmacokinetics (PK) and exposures of subjects in these studies have been characterized by population pharmacokinetic (PPK) analysis of data collected in these studies, together with PK data from several Phase 1, 2, and 3 clinical studies of nivolumab monotherapy in solid tumors. PPK analyses have shown that the PK of nivolumab are linear, with dose proportional exposures over a dose range of 0.1 mg/kg to 10 mg/kg, and are similar across tumor types. Nivolumab clearance and volume of distribution were found to increase with increasing body weight, but the increase was less than proportional, indicating that a mg/kg dose represents an over-adjustment for the effect of body weight on nivolumab PK. Given the relationship between nivolumab PK and body weight, a flat dose is expected to lead to lower exposures in heavier patients, relative to the exposures in lighter patients.

Using the PPK model, nivolumab steady-state trough, peak and time-averaged concentration (Cminss, Cmaxss, and Cavgss, respectively) were predicted for a flat nivolumab dose of 240 mg Q2W and compared to those following administration of 3 mg/kg Q2W in NSCLC, melanoma, and RCC subjects. A dose of 240 mg nivolumab is identical to a dose of 3 mg/kg for subjects weighing ~ 80 kg, which is the approximate median body weight of subjects in the Phase 2 and 3 BMS clinical studies of nivolumab monotherapy. From the simulations, the geometric mean values of Cminss, Cmaxss, and Cavgss with flat dosing are slightly (< 15%) higher than that produced by a 3 mg/kg dose, and the coefficient of variation (cv%) in these measures of exposure are only slightly (< 10%) greater than that of 3 mg/kg dosing. Given the similarity of nivolumab PK across tumor types and the similar exposures predicted following administration of a 240 mg flat dose compared to 3 mg/kg, it is expected that the

safety and efficacy profile of nivolumab following a flat dose will be similar to that of 3 mg/kg nivolumab dose.

Across the various tumor types in the BMS clinical program, nivolumab has been shown to be safe and well tolerated up to a dose level of 10 mg/kg, and the relationship between Nivolumab exposure produced by 3 mg/kg and efficacy has been found to be relatively flat. Taken together, the PK, safety, and efficacy data indicate that the safety and efficacy profile of flat nivolumab dose every 2 weeks will be similar to that of a 3 mg/kg nivolumab every 2 weeks.

In Arm C of this study after completion of the combination portion of the study, all subjects will receive flat dose 480 mg nivolumab every 4 weeks (Q4W), which provides a more convenient dosing regimen for subjects. Based on PK modeling and simulations, administration of nivolumab 480 mg Q4W will be started after steady state is achieved with the combination regimen. While 480 mg Q4W is predicted to provide greater (approximately 20%) maximum steady state concentrations and lower (approximately 10%) steady state trough concentrations, these exposures are predicted to be within the exposure ranges observed at doses up to 10 mg/kg Q2W used in the Phase 1 nivolumab clinical program, and are not considered to put subjects at increased risk. Similar to the nivolumab Q2W dosing monotherapy regimen, the exposures predicted following administration of nivolumab 480 mg Q4W, are on the flat part of the exposure-response curves for previously investigated tumors, melanoma and NSCLC, and are not predicted to affect efficacy. Based on these data, nivolumab 480 mg Q4W is expected to have similar efficacy and safety profiles to nivolumab 3 mg/kg Q2W.

Hence, doubling the dose of nivolumab from 240 mg to 480 mg would extend the dosing interval from 2 weeks to 4 weeks. Thus a flat dose of 480 mg every 4 weeks is recommended for Arm C of this study. FDA approval of Nivolumab 480mg IV every 4 weeks was obtained on March 6, 2018.

2.4 Relatlimab (anti-LAG-3)

LAG-3 is an inhibitory checkpoint molecule found on the surface of antigen presenting cells which may limit the anti-tumor activity of PD-1 blockade.

Relatlimab as a single agent has had an acceptable safety profile at all tested doses: 20, 80, 240, and 800 mg, flat dose. The MTD was not reached up to 800 mg relatlimab Q2W. The combination therapy of relatlimab and nivolumab when administered sequentially (nivolumab 30-minute IV infusion followed by relatlimab 1-hour IV infusion) has an acceptable safety profile of all dose combinations that have completed safety testing, up to relatlimab 160 mg/nivolumab 240 mg Q2W. In addition, safety evaluation with alternative less frequent regimen (Q4W) dose escalation cohorts are currently under investigation in Study CA224020. The less frequent dosing regimens of relatlimab are designed to afford more convenience to the target patient population and allow combination with less frequent nivolumab dosing regimens. To date, the sequential administration of relatlimab 160 mg/nivolumab 480 mg Q4W has been shown safe (1 DLT out of six evaluable subject) to move to the next cohort of relatlimab 240 mg/nivolumab 480 mg Q4W.

Results from a Phase 1 trial using both LAG-3 (BMS-986016) and PD-1 blockade (nivolumab) have been shown to be safe with clinical activity in advanced solid tumor patients (NCT01968109).

Neoadjvuant therapy is a powerful platform to inform efficacy, toxicity and biomarkers of treatment response and resistance. We believe that biomarker driven neoadjuvant clinical trials provide critical insights into immunologic and molecular mechanisms of response and resistance that are essential for each novel combination therapy regimen. These trials combine the practical advantages of limited patient numbers with the ability to use each patient's biospecimens as a robust learning resource.

Prior to Amendment 7, this neoadjuvant trial randomized to single agent nivolumab vs the combination of ipilimumab and nivolumab (BMS CA209-291), showed suboptimal responses with single agent nivolumab with 22% of treated patients progressing to no longer be surgically resectable during the 8 week duration of neoadjuvant treatment but also grade 3 toxicity rate of 89% during neoadjuvant ipilimumab with nivolumab. It is hypothesized that use of LAG-3 with PD-1 blockade may provide optimal efficacy with limited toxicity in the neoadjuvant treatment setting based on the below Phase 1 trial data.

Study CA224020 is a Phase 1 dose escalation and cohort expansion study of the safety, tolerability, and efficacy of relatlimab administered alone and in combination with nivolumab in advanced solid tumors. As of cut-off date of 07-Apr-17, preliminary proof-of-concept efficacy has been revealed in Part C in the combination treatment expansion cohort of advanced melanoma with prior treatment with anti-PD-1/PD-L1. All subjects were treated with relatlimab 80 mg + nivolumab 240 mg every 2 weeks. The overall ORR was 11.5% (n=61 response evaluable) with a disease control rate of 49%. Biomarker analyses showed that patients whose tumor associated immune cells expressed more LAG-3 had a higher response rate, with a greater than 3-fold increase in ORR observed in patients with evidence of LAG-3 expression in at least 1% of nucleated cells within the tumor margin, compared to less than 1% LAG-3 expression (ORRs of 18.2% [6/33] and 5.0% [1/20], respectively). PD-L1 expression did not appear to enrich for response.

The treatment group had the following characteristics: 1) Most patients had M1C disease (69%), 2) the cohort was heavily pretreated (76% with 2 or more prior therapies), 3) all patients had progressed while receiving anti-PD-1/PD-L1, and 4) progressive disease was the best response to prior anti-PD-1/PD-L1 in 40% of patients. Overall, relatlimab in combination with nivolumab demonstrated encouraging initial efficacy with a safety profile similar to nivolumab monotherapy.

In summary, relatlimab in combination with nivolumab has shown the capacity to induce responses in heavily treated advanced solid tumors, with the added ability to trigger responses in tumors that have demonstrated resistance to nivolumab therapy. It is hypothesized that response rates will be higher in the neoadjuvant setting this therapy will be applied to patients who are not exposed to prior anti PD-1 or PD-L1 blockade.

2.5 Safety of Combination Checkpoint Blockade

There is ample preclinical data supporting dual checkpoint blockade in murine cancer models.[56-61] Based on these principles, investigators have initiated trials of dual checkpoint blockade for multiple malignancies.

Preliminary phase I results of combination of nivolumab and ipilimumab in patients with advanced melanoma demonstrate the potential of this combination.62 The trial treated 86 patients with concurrent (n=53) dose-escalation of the two agents or sequenced treatment (n=33) with nivolimumab in patients previously treated with ipilimumab. In the concurrent arm, treatment was dosed at 0.3 mg/kg of nivolumab and 3 mg/kg of ipilimumab (cohort 1), 1 mg/kg of nivolumab and 3mg/kg of ipilimumab (cohort 2), 3 mg/kg of nivolumab and 1 mg/kg of ipilimumab (cohort 2a), 3mg/kg of nivolumab and 3 mg/kg of ipilimumab (cohort 3). Dose-limited toxicity was observed in cohort 3, so cohort 2 was treated as the maximum tolerated dose. The concurrent treatment demonstrated considerably higher rates of adverse events than previous trials of either drug in monotherapy. Treatment-related adverse events were noted in 93% of patients, serious treatment-related adverse events (≥ grade 3) were seen in 53% of patients, and 21% of patients discontinued therapy secondary to these toxicities. The types of irAEs observed were similar to those seen in both nivolumab and ipilimumab monotherapy trials. The adverse events were well managed with immunosuppressant medication and hormonal replacement therapy (for endocrinopathies) and there were no treatment-related deaths observed. In the concurrent arm, 21 of the 53 patients (40%) were noted to have a response by mWHO criteria (the primary endpoint) with the suggestion of a higher response rate when irRC and unconfirmed responses are included. Remarkably, 16 (76%) of those with an objective response had a tumor reduction of 80% or more with five complete responses noted. [62] Longer-term follow up has demonstrated a complete response rate of 17% and a 1 and 2 year OS of 82% and 75%, respectively. Furthermore, the 2a cohort (3mg/kg nivolumab and 1mg/kg of ipilimumab) has demonstrated a 1 and 2 year OS of 94% and 88%, respectively. This dosing group has been selected for further study with expanded enrollment and on-going follow-up.[63]

Two additional trials of ipilimumab and nivolumab in combination are being conducted in non-small cell lung cancer (NSCLC) and metastatic renal cell carcinoma. In both trials patients were given either nivolumab 3mg/kg with ipilimumab 1mg/kg or nivolumab 1mg/kg and ipilimumab 3mg/kg for every 3 weeks for four cycles followed by nivolumab 3mg/kg every two weeks until progression or unacceptable toxicity. In NSCLC, 46 treatment naïve patients were enrolled at initial reporting. Grade 3-4 toxicity was seen in 48% of patients with 3 treatment related deaths and 16 discontinuing treatment secondary to toxicity. Overall, 22% of patients achieved an objective response and, at the interim reporting of results, three of the dosing cohorts had not achieved median duration of response due to ongoing responses (9+-21+ weeks).[64] In the metastatic renal cell carcinoma trial, 44 patients were enrolled at initial reporting. Grade 3-4 toxicity was seen in 43% of patients. No patients died from treatment related toxicity and seven discontinued treatment due to toxicity. An objective response was seen in 34% of patients with 14/15 responses on-going (4.1+-21+ weeks) at the time of last reporting.[65]

On September 30, 2015 the FDA granted accelerated approval to the use of ipilimumab in conjunction with nivolumab for patients with BRAF wild type, unresectable or metastatic melanoma. This was based on a randomized double blinded clinical trial showing response rates of 60% for combination treated patients vs. 11% for ipilimumab treated patients. PFS for patients on the combination was 8.9 months vs. 4.7 months for ipilimumab treated patients.[66]

Safety of the combination of nivolumab and relatlimab was assessed in study CA224020. Overall, based on preliminary data as of the clinical cutoff date of 15-Jun-2017, the safety profile of relatlimab in combination with nivolumab is manageable, with no maximum tolerated dose (MTD) reached at the tested doses up to 160 mg relatlimab and 240 mg nivolumab (flat

dose, every 2 weeks [Q2W]), with evaluation of the 240 mg relatlimab/240 mg nivolumab combination doselevel ongoing. At the time of the clinical cutoff date (15-Jun-2017) one dose-limiting toxicity (DLT) of Grade 5 myocarditis was observed at the 240 mg relatlimab/240 mg nivolumab combination dose level among five evaluable subjects. There was no dose relationship between the incidence, severity, or causality of adverse events (AEs) to combination therapy. In the nine expansion cohorts, a total of 262 subjects were treated with 80 mg relatlimab and 240 mg nivolumab Q2W. Most AEs were low grade (Grade 1 to Grade 2) with a total of 26 subjects experiencing a drug-related serious adverse event (SAE). All AEs, except for one Grade 5 myocarditis and one Grade 4 drug-induced liver injury (DILI), were reversible and manageable by withholding study drug administration providing standard medical care, and/or following immunerelated AE algorithms. In summary the safety profile of the combination of relatlimab and nivolumab appears similar to nivolumab monotherapy in terms of both frequency and severity of AEs.

A pattern of immune-related adverse events has been defined for treatment with nivolumab monotherapy and nivolumab in combination with other immune-targeting agents such as relatlimab. Management algorithms have been developed for these events and are provided in the Appendix. Most high-grade events are manageable with the use of corticosteroids or hormone replacement therapy (endocrinopathies) as instructed in these algorithms.

Myocarditis has been observed with nivolumab monotherapy treatment (see nivolumab IB). Given the grade 5 myocarditis event in the CA224020 study, and the known nonclinical mouse double LAG-3/PD-1 knockout myocarditis phenotype, increased cardiac surveillance with troponin measurements were instituted. As of the clinical cutoff date of 15-Jun-2017 there have been four grade 1 myocarditis cases (asymptomatic troponin elevations with imaging correlate of myocardial inflammation but without evidence of cardiac dysfunction). Treatment was delayed in all cases, and precautionary steroid treatment was given without any of the participants developing evidence of cardiac dysfunction.

Additional details on the safety profiles of relatlimab and nivolumab, including guidance regarding myocarditis and other potential risks, as well as results from other clinical studies, are available in the relatlimab and nivolumab IBs.

2.6 Neoadjuvant Approach

Increasingly, systemic therapy is being given in the neoadjuvant setting (prior to surgery) in a variety of cancer types including breast, rectal, and esophageal cancers. There are many practical and theoretical advantages to giving systemic therapy up front. First, giving systemic therapy prior to surgery allows patients to receive and complete the entire course of systemic therapy because there are no potential complications and deconditioning that are inherent to major operative interventions. Second, up front systemic treatment can shrink the tumor and increase the likelihood that the entire tumor is removed. Next, obtaining the tumor specimen after it has been exposed to systemic treatment allows for an assessment of response to treatment which can be used as a prognostic marker or, potentially, to direct further systemic therapy. From a scientific standpoint, this tissue can also be used to help elucidate drug mechanism of action and determine biomarkers that can predict responsiveness to therapy.

In immunotherapy, in general, and checkpoint blockade, in particular, there are theoretical advantages to giving therapy prior to tumor removal. Neoadjuvant immunotherapy may be more effective than immunotherapy given in the immediate postoperative period as patients will experience some level immunosuppression as a consequence of surgery.[67] Additionally, more robust antitumor immune responses are expected with checkpoint blockade when the

tumor microenvironment is intact within the host as there is potential exposure to tumor-specific antigens, immuno-adaptability (antigenic epitope spreading) of the immune system to changes in tumor cells, and cytokine signaling that may enhance the immune response. Checkpoint inhibitors augment the body's inherent antitumor immune response by counteracting cancer-related immunosuppression. This allows the immune system to recognize tumor associated antigens and mount an effective response against them. Having the intact tumor in place will allow the immune system greater exposure to the full range of immunogenic tumor epitopes and, thus may lead to a more effective response.

3 Objectives & Endpoints

3.1 Primary Objective

Arm A and Arm B

To assess the pathologic response of nivolumab monotherapy and nivolumab and ipilimumab dual therapy administered in the neoadjuvant setting in patients with clinical stage III or oligometastatic stage IV melanoma. Pathologic response will be assessed by percent viable tumor cells, percent tumor necrosis, presence of fibrosis and melanoma proliferation as assessed by phosphohistone H3 from baseline, to on- treatment and surgical specimen

Arm C

To assess the pathologic response rate of combination relatlimab with nivolumab in the neoadjuvant setting in patients with high-risk resectable Stage IIIB/C or oligometastatic Stage IV melanoma. Pathologic response will be assessed by percent viable tumor cells, percent tumor necrosis, presence of fibrosis and melanoma proliferation as assessed by phosphohistone H3 from baseline, to on- treatment and surgical specimens.

3.2 Secondary Objectives

Arm A and Arm B

- To assess the immunologic response of neoadjuvant nivolumab monotherapy and neoadjuvant nivolumab and ipilimumab dual therapy in patients with high-risk resectable melanoma. Immunologic response will be determined by change in T cell infiltrate from baseline to on-treatment and surgical specimens in response to therapy

- To assess the objective response rate (ORR) of nivolumab monotherapy and nivolumab and ipilimumab dual therapy administered in the neoadjuvant setting as assessed by imaging (RECIST 1.1 criteria) in patients with high-risk resectable melanoma

- To assess the 12-month recurrence-free survival (RFS) and overall survival (OS) of patients with high-risk resectable melanoma treated with neoadjuvant nivolumab

monotherapy or nivolumab and ipilimumab dual therapy followed by adjuvant nivolumab

- To evaluate the safety of nivolumab monotherapy and dual ipilimumab and nivolumab in the neoadjuvant setting and peri-operatively as well as assess the safety of adjuvant nivolumab.

Arm C

- To evaluate safety and feasibility of relatlimab with nivolumab delivered in the neoadjuvant setting
- To assess the objective response rate (ORR) of relatlimab with nivolumab administered in the neoadjuvant setting as assessed by imaging (RECIST 1.1 criteria) in patients with high-risk resectable melanoma.
- To assess the 12-month recurrence-free survival (RFS) and overall survival (OS) of patients with high-risk resectable melanoma treated with neoadjuvant and adjuvant relatlimab with nivolumab
- To evaluate immunologic and molecular mechanisms of response and resistance to relatlimab with nivolumab.

3.3 Exploratory Objectives

Identification of immunologic and genomic markers correlating with clinical and pathological response and resistance

4 Ethical Considerations

4.1 Good Clinical Practice

This study will be conducted in accordance with Good Clinical Practice (GCP), as defined by the International Conference on Harmonisation (ICH) and in accordance with the ethical principles underlying European Union Directive 2001/20/EC and the United States Code of Federal Regulations, Title 21, Part 50 (21CFR50). The study will be conducted in compliance with the protocol. The protocol and any amendments and the subject informed consent will receive Institutional Review Board/Independent Ethics Committee (IRB/IEC) approval/favorable opinion prior to initiation of the study. All potential serious breaches must be reported to BMS immediately. A serious breach is a breach of the conditions and principles of GCP in connection with the study or the protocol, which is likely to affect, to a significant degree, the safety or physical or mental integrity of the subjects of the study or the scientific value of the study. Personnel involved in conducting this study will be qualified by education, training, and experience to perform their respective tasks. This study will not use the services of study personnel where sanctions have been invoked or where there has been scientific misconduct or fraud (e.g., loss of medical licensure, debarment).

4.2 Institutional Review Board/Independent Ethics Committee

Before study initiation, the investigator must have written and dated approval/favorable opinion from the IRB/IEC for the protocol, consent form, subject recruitment materials (e.g., advertisements), and any other written information to be provided to subjects. The investigator or BMS should also provide the IRB/IEC with a copy of the Investigator Brochure or product labeling information to be provided to subjects and any updates. The investigator or BMS should provide the IRB/IEC with reports, updates and other information (e.g., expedited safety reports, amendments, and administrative letters) according to regulatory requirements or institution procedures.

4.3 Informed Consent

Investigators must ensure that subjects are clearly and fully informed about the purpose, potential risks, and other critical issues regarding clinical studies in which they volunteer to participate.

In situations where consent cannot be given to subjects, their legally acceptable representatives are clearly and fully informed about the purpose, potential risks, and other critical issues regarding clinical studies in which the subject volunteers to participate. BMS will provide the investigator with an appropriate (i.e., Global or Local) sample informed consent form which will include all elements required by ICH, GCP and applicable regulatory requirements. The sample informed consent form will adhere to the ethical principles that have their origin in the Declaration of Helsinki.

Investigators must:

1. Provide a copy of the consent form and written information about the study prior to clinical study participation. The language must be non-technical and easily understood.

2. Allow time necessary for subject or subject's legally acceptable representative to inquire about the details of the study.

3. Obtain an informed consent signed and personally dated by the subject or the subject's legally acceptable representative and by the person who conducted the informed consent discussion.

4. Obtain the IRB/IEC's written approval/favorable opinion of the written informed consent form and any other information to be provided to the subjects, prior to the beginning of the study, and after any revisions are completed for new information.

5. If informed consent is initially given by a subject's legally acceptable representative or legal guardian, and the subject subsequently becomes capable of making and communicating his or her informed consent during the study, consent must additionally be obtained from the subject.

6. Inform the patient, that upon completion of the study, Bristol-Myers Squibb will not continue to supply study drug to subjects/investigators. The PI will ensure that the patient will receive appropriate standard of care or other appropriate treatment.

Revise the informed consent whenever important new information becomes available that is relevant to the subject's consent. The investigator, or a person designated by the investigator, should fully inform the subject or the subject's legally acceptable representative or legal guardian, of all pertinent aspects of the study and of any new information relevant to the subject's willingness to continue participation in the study. This communication should be documented.

The confidentiality of records that could identify subjects must be protected, respecting the privacy and confidentiality rules applicable to regulatory requirements, the subjects' signed ICF and, in the US, the subjects' signed HIPAA Authorization.

The consent form must also include a statement that BMS and regulatory authorities have direct access to subject records. Subjects unable to give their written consent (e.g., stroke or subjects with or severe dementia) may only be enrolled in the study with the consent of a legally acceptable representative. The subject must also be informed about the nature of the study to the extent compatible with his or her understanding, and should this subject become capable, he or she should personally sign and date the consent form as soon as possible. The explicit wish of a subject who is unable to give his or her written consent, but who is capable of forming an opinion and assessing information to refuse participation in or to be withdrawn from, the clinical study at any time should be considered by the investigator. The rights, safety, and well-being of the study subjects are the most important considerations and should prevail over interests of science and society.

5 Investigational Plan

5.1 Study Design

This is a randomized Phase II study designed to detect pathologic and immunologic biomarkers of response to checkpoint blockade in melanoma. In Arm A, 12 patients received up to four upfront doses of nivolumab and in Arm B 11 patients received up to three doses of upfront ipilimumab combined with nivolumab. Both groups of patients will have a baseline biopsy and blood for genomic and immunologic analyses. On-treatment biopsies and blood for biomarker analysis will be obtained in both groups around dose 2 and dose 3 (if feasible). Neoadjuvant therapy will be administered over 7 weeks and will then be followed by restaging scans. If at any point during the course of neoadjuvant therapy there is clinical (worsening performance status) and/or objective evidence (new imaging data) to suggest rapid disease progression, the patient will be taken off study and offered immediate surgery or other alternative treatment plan. If disease remains resectable based on the updated scans and the assessment of the treating surgical oncologist, patients in both groups will undergo definitive surgical excision of visible disease. Preliminary assessment of surgical margins will be obtained by intra-operative frozen section when appropriate. If surgical margins are found to be microscopically involved, patients will still be eligible for initiation of adjuvant therapy and no adjuvant radiation will be administered.

All acquired tissue samples (baseline, on-treatment and surgical resection) will be used for biomarker analyses. Patients will be reassessed for initiation of adjuvant nivolumab once adequate wound healing has been achieved within 4-6 weeks of surgical procedure. Both groups of patients will then initiate on six months of adjuvant nivolumab administered every two weeks. Restaging scans will be obtained every 12 weeks.

Patients with clinical stage III (IIIB/IIIC) or oligometastatic stage IV disease will be screened for eligibility. Patients with disease that is determined to be surgically resectable as agreed upon by a multidisciplinary consensus conference will be eligible to be screened for the protocol. Disease will be considered unresectable if there is significant vascular, neural or bony involvement and in cases where a complete surgical resection with negative margins cannot be safely performed. Prior to Amendment 7, forty patients were expected to be randomized in a 1:1 ratio between Arm A and Arm B. Subjects would then be stratified by stage of disease (IIIB, IIIC, M1A vs. M1B/M1C) and by PD-L1 + or PD-L1 – tumor status).

After approval of **Amendment 7**, Arms A and B were closed to new patient entry and Arm C was opened to enrollment. Thirty patients will be enrolled to Arm C without randomization.

Figures 1a and 1b Study Design Schema

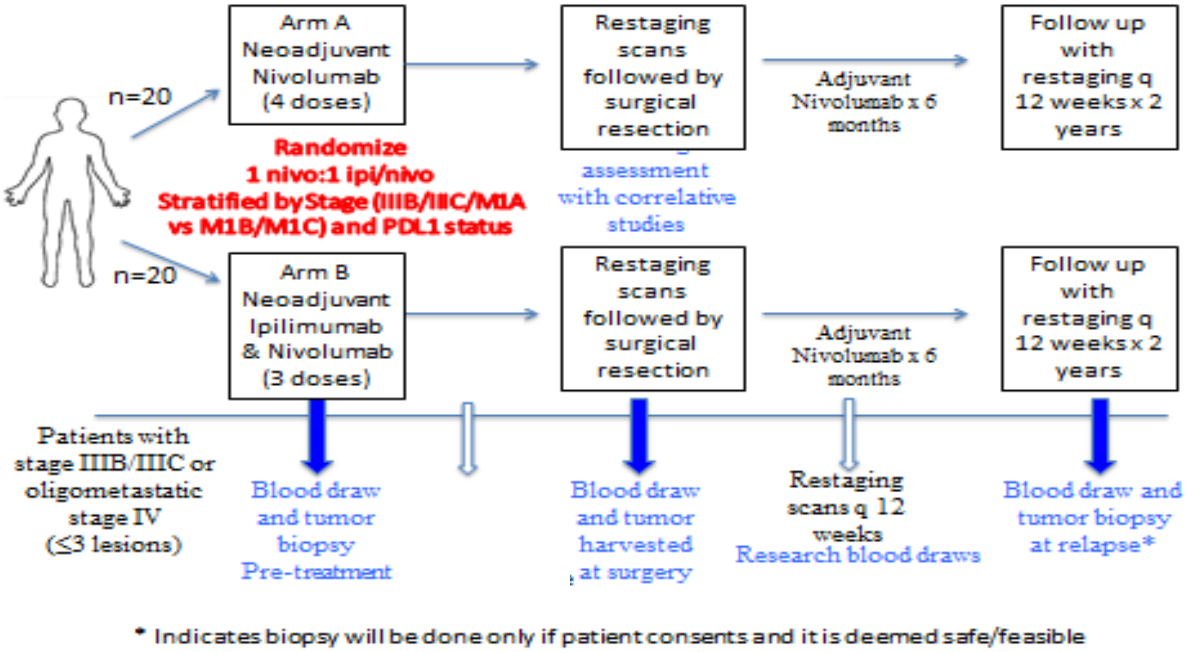

Based on analysis by Data Safety Monitoring Board at a meeting on July 11, 2017, enrollment on Arm A and B was closed based on efficacy (Arm A) and safety (Arm B) concerns. At the time of study closure, 23 total patients were enrolled and treated; 12 in Arm A and 11 in Arm B. In Arm A, 22% of patients experienced disease progression and were ineligible for surgery prompting closure of this Arm. In Arm B, all patients have been able to proceed to surgery, however the grade 3 toxicity rate was 89% and 33% required delay in surgery related to toxicity. This Arm was closed due to concerns of toxicity and patient safety. Based on the above results, Arm C was created to explore the safety and efficacy of alternative dosing strategies to maximize efficacy while minimizing toxicity.

In Arm C, 30 patients will receive relatlimab combined with nivolumab every 28 days for 2 doses prior to surgery. The biospecimen collection plan will include blood and tumor

collections at day 15 and day 29. Neoadjuvant therapy will be administered and will then be followed by restaging scans. If at any point during the course of neoadjuvant therapy there is clinical (worsening performance status) and/or objective evidence (new imaging data) to suggest rapid disease progression, the patient will be taken off study and offered immediate surgery or other alternative treatment plan. If disease remains resectable based on the updated scans and the assessment of the treating surgical oncologist, patients will undergo definitive surgical excision of visible disease. In the adjuvant setting, patients will receive relatlimab and nivolumab every 28 days for an additional 10 doses. Restaging scans will be obtained every 12 weeks in the adjuvant setting.

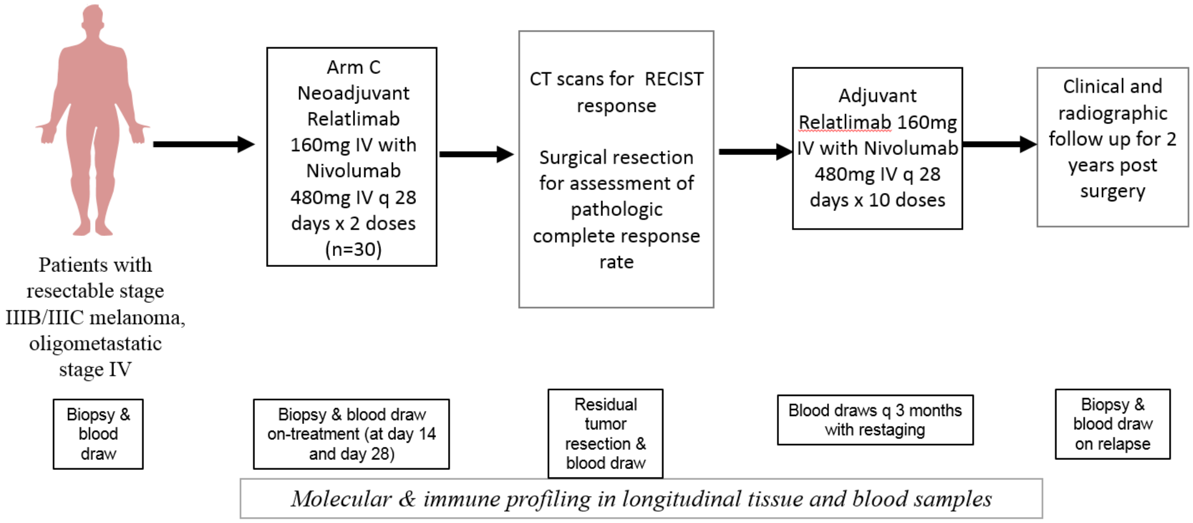

5.2 Study Assessments

The primary objective of this study is assessment of pathologic changes from baseline to on-treatment samples within each treatment arm. Blood and tumor collections are required at baseline (samples must be obtained within 28 days of treatment initiation), around dose 2 and dose 3 of neoadjuvant therapy in Arms A and B, and again at the time of definitive surgical excision. Biopsies around dose 3 for Arms A and B and at relapse are optional and will be done if medically safe and feasible. Patients in arms A and B will undergo adjuvant nivolumab. Biopsies around dose 3 for Arms A and B and at relapse are optional and will be done if medically safe and feasible.

In Arm C, blood and tumor are required at baseline (samples must be obtained within 28 days of treatment initiation), at day 15 and day 29 of neoadjuvant therapy and at the time of definitive surgical excision. Patients in Arm C will then undergo 10 months of adjuvant relatlimab and nivolumab.

For all treatment arms, research blood will be obtained every three months during the duration of adjuvant therapy. Blood and tumor will also be collected at the time of relapse for all treatment arms when possible.

Secondary objectives of this study include assessment of immunologic changes in the tumor microenvironment and blood, treatment response based on imaging in response to neoadjuvant therapy, toxicity, RFS and OS. Subjects will be assessed with computed tomography (CT) or magnetic resonance imaging (MRI) at screening, after completion of

neoadjuvant therapy and during the post-treatment follow-up period (refer to the Study Procedure Tables in Section 7.1 for scanning frequency). Subjects will also be followed for survival. Subjects will be followed for a minimum of two years. Safety will be evaluated by clinical assessments including vital signs and complete physical examinations, chemistry and hematology laboratory values and formal assessments of adverse events (AEs).

5.3 Treatment Assignment

Subjects will be approved for treatment after a consensus panel of medical and surgical oncologists have determined that the disease is amenable to surgical resection and after the subject has passed screening evaluations (see Section 5.4). Subjects will then be assigned a subject number and will be eligible for randomization to Arm A or Arm B. Subject randomization will be implemented by the Clinical Trial Conduct website maintained by the Department of Biostatistics at the University of Texas M.D. Anderson Cancer Center (https://biostatistics.mdanderson.org/ClinicalTrialConduct). Once a subject number has been assigned, it cannot be reassigned to any other patient. If the subject is prematurely discontinued from the study without having received the prescribed treatment, an additional subject may be enrolled as a replacement subject.

There will be no randomization for patients in Arm C.

5.4 Eligibility Criteria

5.4.1 Inclusion Criteria

A subject will be eligible for inclusion in this study only if all of the following criteria apply:

1. Capable of giving written informed consent, which includes compliance with the requirements and restrictions listed in the consent form

2. Patients must have histologically or cytologicaly confirmed Stage IIIB/C or Stage IV oligometastatic melanoma. Oligometastatic melanoma is defined as three or fewer areas of resectable disease excluding central nervous system and bone involvement. Patients with cutaneous, mucosal, acral, ocular or unknown primary melanomas are eligible for enrollment. For patients with stage IV disease with distant lymph nodes (stage M1a), a maximum of three separate lymph node sites fit the definition of oligometastatic disease. Resectable tumors are defined as having no significant vascular, neural or bony involvement. Only cases where a complete surgical resection with tumor-free margins can safely be achieved are defined as resectable. Details on melanoma staging per the American Joint Committee on Cancer Version 7 can be found in Appendix 1.

3. Patients will have at least one melanoma deposit that can undergo serial biopsy (at least 2 time points) during the neoadjuvant phase of the protocol. Patients must be willing to provide tumor samples at the time points specified in the Study Procedure Tables (Section 7.1).

4. All patients must undergo a baseline tumor biopsy. In Arms A and B, tumor biopsy for PD-L1 testing (PD-L1 positivity is determined by greater than or equal to 1% of cells staining in the membrane by immunohistochemistry) is required for stratification. PD-L1 status is not required for enrollment on Arm C. The 28-8 clone for PD-L1 testing

is required for assessment of PD-L1 status. For patients with stage IV disease, site of tumor biopsy will preferably be from non-lymph node disease site. For PD-L1 testing, the biopsy should contain sufficient tumor content (>100 tumor cells/4-micron tissue section). If a sample contains insufficient tumor content, a re-biopsy will be required to obtain a sample with sufficient tumor content prior to treatment.

5. Patients must be medically fit enough to undergo surgery as determined by the treating medical and surgical oncology team

6. Patients who have been previously treated in the adjuvant setting for melanoma will be eligible for treatment after a 28 day wash-out period

7. Patients must have measurable disease, defined by RECIST 1.1 (Appendix 2)

8. Age ≥ 18 years

9. ECOG performance status 0-1

10. Patients must have organ and marrow function as defined below within 28 days of first study treatment:

Table 1 Eligibility Guidelines of Organ and Marrow Function

System	Laboratory Values
**Hematologic**	
Absolute neutrophil count (ANC)†	≥ 1.5 X 10^9/L
Hemoglobin‡	≥ 8.5 g/dL
Platelets‡	≥ 100 X 10^9/L (≥ 60 for HCC)
PT/INR and PTT	≤ 1.5 X ULN
WBC†	≥ 2.0 x 10^9/L
**Hepatic**	
Total bilirubin	≤ 1.5 X ULN (except subjects with Gilbert's Syndrome who must have normal direct bilirubin) [3 mg/dL for HCC]
AST and ALT	≤ 3.0 X ULN (≤ 5 x ULN for HCC)
Albumin	≥2.5 g/dL
**Renal**	

Creatinine<br>OR<br>Calculated creatinine clearance<br>OR<br>24-hour urine creatinine clearance	≤1.5 X ULN<br><br>≥ 40 mL/min<br><br>≥ 50 mL/min
**Other**	
Lipase<br>Amylase<br><br>Normal Thyroid Function (or stable on hormone supplementation)<br><br>LVEF	< 1.5 X ULN<br>< 1.5 X ULN<br><br>0.27 – 10 X 10^9/L<br><br>Assessment with documented LVEF ≥ 50% by either TTE (preferred test) or MUGA within 6 months from first study drug administration
† = stable off any growth factor within 28 days of first study drug administration.<br>‡ = transfusion to achieve this level is not permitted within 2 weeks of first study drug administration	

Women are eligible if:

11. Non-childbearing potential defined as pre-menopausal females with a documented tubal ligation or hysterectomy; or postmenopausal defined as 12 months of spontaneous amenorrhea [in questionable cases a blood sample with simultaneous follicle stimulating hormone (FSH) > 40 MIU/mL and estradiol < 40 pg/mL (<140 pmol/L) is confirmatory]. Females on hormone replacement therapy (HRT) and whose menopausal status is in doubt will be required to use one of the contraception methods in Appendix 3 if they wish to continue their HRT during the study. Otherwise, they must discontinue HRT to allow confirmation of post-menopausal status prior to study enrollment. For most forms of HRT, at least 2-4 weeks will elapse between the cessation of therapy and the blood draw; this interval depends on the type and dosage of HRT. Following confirmation of their post-menopausal status, they can resume use of HRT during the study without use of a contraceptive method.

12. Childbearing potential and agrees to use method(s) of contraception. For a teratogenic study drug and/or when there is insufficient information to assess teratogenicity (preclinical studies have not been done), a highly effective method(s) of contraception (failure rate of less than 1% per year) is required.

    The individual methods of contraception and duration should be determined in consultation with the investigator. Women of childbearing potential (WOCBP) must follow instructions for birth control when the half-life of the investigational drug is greater than 24 hours, contraception should be continued for a period of 30

days plus the time required for the investigational drug to undergo five half-lives. The half-life of nivolumab and ipilimumab is up to 25 days and 18 days, respectively. WOCBP should use an adequate method to avoid pregnancy for 24 weeks (30 days plus the time required for nivolumab and/or relatlimab to undergo five half-lives) after the last dose of investigational drug. WOCBP must have a negative serum or urine pregnancy test (minimum sensitivity 25 IU/L or equivalent units of HCG) within 24 hours prior to the start of investigational product

13. Women must not be breastfeeding

14. Men who are sexually active with WOCBP must use any contraceptive method with a failure rate of less than 1% per year The investigator shall review contraception methods and the time period that contraception must be followed. Men who are sexually active with WOCBP must follow instructions for birth control when the half-life of the investigational drug is greater than 24 hours, contraception should be continued for a period of 90 days plus the time required for the investigational drug to undergo five half-lives. The half-life of nivolumab and ipilimumab is up to 25 days and 18 days, respectively. Therefore, men who are sexually active with WOCBP must continue contraception for 33 weeks (90 days plus the time required for nivolumab and/or relatlimab to undergo five half-lives) after the last dose of investigational drug. In addition, male participants must be willing to refrain from sperm donation during this time.

    Men who are sexually active with women who are not of childbearing potential (i.e., who are postmenopausal or surgically sterile and azoospermic men) do not require contraception.

    Further guidance on contraception practices can be found in Appendix 3.

15. For Arm C: Cardiac assessment at baseline by trans-thoracic echocardiogram (TTE) with LVEF$\geq$ 50%.

5.4.2 <u>Exclusion Criteria</u>

A subject will not be eligible for inclusion in this study if any of the following criteria apply:

1. Currently receiving cancer therapy (chemotherapy, radiation therapy, immunotherapy, or biologic therapy) or investigational anti-cancer drug

2. Any major surgery within the last 3 weeks

3. Brain metastases, leptomeningeal disease or bone metastases

4. Pregnant or lactating female

5. Unwillingness or inability to follow the procedures required in the protocol

6. Current use of anticoagulants (warfarin, heparin, direct thrombin inhibitors) at therapeutic levels

7. Any serious or uncontrolled medical disorder that, in the opinion of the investigator, may increase the risk associated with study participation or study drug administration, impair the ability of the subject to receive protocol therapy, or interfere with the interpretation of study results.

8. Prior malignancy active within the previous 3 years except for patient's prior diagnosis of melanoma and locally curable cancers that have been apparently cured, such as basal or squamous cell skin cancer, superficial bladder cancer, or carcinoma in situ of the prostate, cervix, or breast with local control measures (surgery, radiation).

9. Subjects with active, known or suspected autoimmune disease. Subjects with vitiligo, type I diabetes mellitus, residual hypothyroidism due to autoimmune condition only requiring hormone replacement, psoriasis not requiring systemic treatment, or conditions not expected to recur in the absence of an external trigger are permitted to enroll.

10. Subjects with a condition requiring systemic treatment with either corticosteroids (> 10 mg daily prednisone equivalents) or other immunosuppressive medications within 14 days of study drug administration. Inhaled or topical steroids and adrenal replacement doses > 10 mg daily prednisone equivalents are permitted in the absence of active autoimmune disease.

11. Prior treatment with an anti-PD-1, anti-PD-L1, anti-LAG-3 or anti-CTLA-4 antibody

12. Any positive test result for hepatitis B or C virus indicating acute or chronic infection

13. Known history of testing positive for human immunodeficiency virus or known acquired immunodeficiency syndrome

14. History of severe hypersensitivity reaction to any monoclonal antibody

15. Prisoners or subjects who are involuntarily incarcerated

16. Subjects who are compulsorily detained for treatment of either a psychiatric or physical (infection disease) illness

17. A known or underlying medical condition that, in the opinion of the Investigator, could make the administration of the study drug hazardous to the subject or could adversely affect the ability of the subject to comply with or tolerate the study.

18. A confirmed history of encephalitis, meningitis, or uncontrolled seizures in the year prior to informed consent.

19. Evidence of active infection that requires systemic antibacterial, antiviral, or antifungal therapy ≤ 7 days prior to initiation of study drug therapy.

20. Any other acute or chronic medical illness.

21. Subjects who are unable to undergo venipuncture and/or tolerate venous access.

22. Any other sound medical, psychiatric, and/or social reason as determined by the Investigator

23. Any of the following procedures or medications:

    a. Within 2 weeks prior to time of study treatment:

        i. Systemic or topical corticosteroids at immunosuppressive doses (> 10 mg/day of prednisone or equivalent). Inhaled or topical steroids, and adrenal replacement steroid doses of > 10 mg daily prednisone equivalent, are permitted in the absence of active autoimmune disease.

        ii. Palliative radiation or gamma

    b. Within 4 weeks prior to study drug administration:

        i. Any investigational cytotoxic drug. Exposure to any non-cytotoxic drug within 4 weeks or 5 half-lives (whichever is shorter) is prohibited. If 5 half-lives is shorter than 4 weeks, agreement with Sponsor/Medical Monitor is mandatory.

24. Subjects with history of life-threatening toxicity related to prior immune therapy (e.g., anti-CTLA-4 or anti-PD-1/PD-L1 treatment or any other antibody or drug specifically targeting T-cell co-stimulation or immune checkpoint pathways) except those that are unlikely to re-occur with standard countermeasures (e.g., hormone replacement after endocrinopathy).

25. Troponin T (TnT) or I (TnI) > 2 x institutional upper limit of normal (ULN). Subjects with TnT or TnI levels between > 1 to 2 x ULN will be permitted if repeat levels within 24 hours are ≤ 1 x ULN. If TnT or TnI levels are > 1 to 2 x ULN within 24 hours, the subject may undergo a cardiac evaluation and be considered for treatment, following a discussion with the investigator or designee. When repeat levels within 24 hours are not available, a repeat test should be conducted as soon as possible If TnT or TnI repeat levels beyond 24 hours are < 2 x ULN, the subject may undergo a cardiac evaluation and be considered for treatment, following a discussion with the investigator or designee.

26. For Arm C: Uncontrolled or significant cardiovascular disease including, but not limited to, any of the following: i) Myocardial infarction (MI) or stroke/transient ischemic attack (TIA) within the 6 months prior to consent ii) Uncontrolled angina within the 3 months prior to consent iii) Any history of clinically significant arrhythmias (such as ventricular tachycardia, ventricular fibrillation, or torsades de pointes) iv) QTc prolongation > 480 msec v) History of other clinically significant cardiovascular disease (i.e., cardiomyopathy, congestive heart failure with New York Heart Association [NYHA] functional classification III-IV, pericarditis, significant pericardial effusion, significant coronary stent occlusion, deep venous thrombosis, etc) vi) Cardiovascular disease-related requirement for daily supplemental oxygen vii) History of two or more MIs OR two or more coronary revascularization procedures viii) Subjects with history of myocarditis, regardless of etiology

6 Treatment Plan

6.1 Investigational Product

The investigational products are supplied by BMS. The investigational products should be stored in a secure area according to local regulations. It is the responsibility of the investigator to ensure that investigational product is only dispensed to study subjects. The investigational product must be dispensed only from official study sites by authorized personnel according to local regulations.

In this protocol, investigational products are: nivolumab (BMS-936558), ipilimumab (BMS-734016), and relatlimab (BMS-986016).

6.2 Handling and Dispensing

The product storage manager should ensure that the study drug is stored in accordance with the environmental conditions (temperature, light, and humidity) as determined by BMS. If concerns regarding the quality or appearance of the study drug arise, the study drug should not be dispensed and contact BMS immediately.

Investigational product documentation must be maintained that includes all processes required to ensure drug is accurately administered. This includes documentation of drug storage, administration and, as applicable, storage temperatures, reconstitution, and use of required processes (e.g., required diluents, administration sets).

Infusion-related supplies (e.g., IV bags, in-line filters, 0.9% NaCl solution) will not be supplied by the sponsor and should be purchased locally if permitted by local regulations.

Please refer to the current version of the Investigator Brochure and/or pharmacy reference sheets for complete storage, handling, dispensing, and infusion information for BMS-936558 (nivolumab) and ipilimumab.

6.3 Destruction

Investigator drug destruction is allowed provided the following minimal standards are met:

- On-site disposal practices must not expose humans to risks from the drug.
- On-site disposal practices and procedures are in agreement with applicable laws and regulations, including any special requirements for controlled or hazardous substances
- Written procedures for on-site disposal are available and followed. The procedures must be filed with the Sponsor SOPs and a copy provided to BMS upon request.
- Records are maintained that allow for traceability of each container, including the date disposed of, quantity disposed, and identification of the person disposing the containers. The method of disposal, i.e., incinerator, licensed sanitary landfill, or licensed waste disposal vendor must be documented.

- Accountability and disposal records are complete, up-to-date, and available for BMS to review throughout the clinical trial period as per the study agreement.

- It is the Investigator's responsibility to arrange for disposal of all empty containers, provided that procedures for proper disposal have been established according to applicable federal, state, local, and institutional guidelines and procedures, and provided that appropriate records of disposal are kept.

6.4 Product Descriptions

6.4.1 Nivolumab (BMS-936558)

Nivolumab (BMS-936558) vials must be stored at a temperature of 2°C to 8°C and should be protected from light and freezing, and shaking. If stored in a glass front refrigerator, vials should be stored in the carton. Recommended safety measures for preparation and handling of nivolumab include laboratory coats and gloves.

For details on prepared drug storage and use time of nivolumab under room temperature/light and refrigeration, please refer to the BMS-936558 (nivolumab) Investigator Brochure.

Care must be taken to assure sterility of the prepared solution as the product does not contain any anti-microbial preservative or bacteriostatic agent. No incompatibilities between nivolumab and polyolefin bags have been observed.

Nivolumab is to be administered approximately as a 30-minute IV infusion with a 0.2-1.2 micron in-line filter at the protocol-specified dose when administered as a single agent. It is administered for 60 minutes when given with ipilimumab or relatlimab. The drug can be diluted with 0.9% normal saline or 5% dextrose injection for delivery but the total drug concentration of the solution cannot be below 0.35 mg/ml. It is not to be administered as an IV push or bolus injection. At the end of the infusion, flush the line with a sufficient quantity of normal saline. For further details, please refer to the current BMS-936558 (nivolumab) Investigator Brochure.

6.4.2 Ipilimumab

Ipilimumab injection can be used for IV administration without dilution after transferring to a PVC (polyvinyl chloride), non-PVC/non-DEHP (di-(2-ethylhexyl)phthalate) or glass containers and is stable for 24 hours at 2-8ºC or room temperature/room light (RT/RL). For ipilimumab storage instructions, refer to ipilimumab IB and/or pharmacy reference sheets.

Recommended safety measures for preparation and handling include protective clothing, gloves, and safety cabinets.

Ipilimumab is to be administered approximately as a 90-minute IV infusion, using a volumetric pump with a 0.2 to 1.2 micron in-line filter at the protocol-specified dose. The drug can be diluted with 0.9% normal saline or 5% Dextrose Injection to concentrations between 1 mg/mL and 4 mg/mL. It is not to be administered as an IV push or bolus injection. Care must be taken to assure sterility of the prepared solutions, since the drug product does not contain any antimicrobial preservatives or bacteriostatic agents. At the end of the

infusion, flush the line with a sufficient quantity of normal saline or 5% dextrose solution. Please also refer to the current ipilimumab Investigator brochure for further details.

When both study drugs are to be administered on the same day, separate infusion bags and filters must be used for each infusion. Nivolumab is to be administered first. The nivolumab infusion must be promptly followed by a saline flush to clear the line of nivolumab before starting the ipilimumab infusion.

If concerns regarding the quality or appearance of the investigational product arise, the investigational product will not be administered and BMS will be contacted immediately. If the study drug(s) are to be destroyed on site, it is the investigator's responsibility to ensure that arrangements have been made for disposal, and that procedures for proper disposal have been established according to applicable regulations, guidelines, and institutional procedures.

6.4.3 Relatlimab (BMS-986016)

Relatlimab should be stored at 2°C to 8°C (36°F to 46°F) with protection from light. Do not freeze the drug product.

Relatlimab is to be administered combined with nivolumab in the same bag as a 60 minute IV infusion through a 0.2/1.2-μm pore size, low-protein-binding polyethersulfone membrane in-line filter at the protocol-specified doses. The Relatlimab and nivolumab injection can be diluted with 0.9% sodium chloride injection (normal saline), to protein concentrations no lower than 1.33 mg/mL. Detailed instructions for drug product dilution and administration are provided in the pharmacy manual for the clinical study. See Section 6.5 for protocol specific administration details.

Care must be taken to assure sterility of the prepared solution, as the products do not contain any anti-microbial preservative or bacteriostatic agent. No incompatibilities between the co-administered drug products and polyolefin or PVC bags, or non-DEHP or PVC infusion sets have been observed.

The administration of relatlimab and nivolumab must be completed within 24 hours of preparation. If not used immediately, the infusion solution may be stored in a refrigerator at 2°C to 8°C (36°F to 46°F) for up to 24 hours and a maximum of 8 hours of the total 24 hours can be at room temperature (20°C to 25°C; 68°F to 77°F) and exposed to room light. The maximum 8-hour period under room temperature and room light conditions includes the product administration period.

Table 2 Product Descriptions

Product Description and Dosage Form	Potency	Primary Packaging (Volume)/Label Type	Secondary Packaging (Qty)/Label Type	Appearance	Storage Conditions (per label)
Nivolumab	100mg (10mg/mL)	10mL vial Open-Label	5 or 10 vials per carton Open- label	Clear to opalescent, colorless to pale yellow liquid. May contain particles	2 to 8ºC. Protect from light and freezing
Ipilimumab	200mg (5mg/mL)	40mL vial Open-Label	5 vials per carton Open- label	Clear, colorless to pale yellow liquid. May contain particles	2 to 8ºC. Protect from light and freezing
Relatlimab	80 mg or 100mg (10mg/mL)	8mL or 10mL vial Open-Label	4 vials per carton Open- label	Clear to opalescent, colorless to pale yellow liquid. May contain particles	2 to 8ºC. Protect from light and freezing

6.5 Dose Calculations and Administration

The first dose of study medication(s) is to be administered within 7 days of randomization.

For Arms A and B prior to Amendment 7, a total of 40 patients were expected to be randomized 1:1 on two different treatment arms. After approval of Amendment 7, Arms A and B will be closed and Arm C will be added. A total of 30 patients will be treated with nivolumab and relatlimab without randomization. There will be two different phases of drug administration.

1) Neoadjuvant phase:

- Arm A: nivolumab 3 mg/kg IV every 2 weeks (+/-7 days) on weeks 1, 3, 5 and 7
- Arm B: nivolumab 1 mg/kg IV combined with ipilimumab 3 mg/kg IV every 3 weeks (+/-7 days) on weeks 1, 4 and 7.
- Arm C: nivolumab 480 mg IV combined with relatlimab 160 mg IV every 28 days (±7 days) beginning on week 1 for 2 doses (Week 1 and Week 5)

2) Adjuvant phase:

- Patients in treatment arms A and B will receive nivolumab 3mg/kg IV every 2 weeks (±7 days) for a total of 24 weeks (13 doses)

- Patients in Arm C will receive nivolumab 480 mg IV combined with relatlimab 160 mg IV every 28 (+/-7 days) days for a total of 10 doses.

For Treatment Arms A and B, the dosing calculations should be based on the actual body weight. If the subject's weight on the day of dosing differs by > 10% from the weight used to calculate the original dose, the dose must be recalculated using the patient's new actual body weight. All doses should be rounded to the nearest milligram. There will be no dose modifications allowed. Please refer to the current Investigator Brochure for additional details on administration.

6.5.1 Dose Modifications

Dose reductions or dose escalations are not permitted.

6.5.2 Dose Delay Criteria

Because of the potential for clinically meaningful treatment-related AEs requiring early recognition and prompt intervention, management algorithms have been developed for suspected AEs of selected categories**.** The PI or study doctor will proceed with surgery if still feasible despite side-effect related dose delay.

Study drug administration should be interrupted and increased monitoring of subjects should ensue if any of the following drug related adverse event(s) occurs:

- Grade ≥1 myocarditis
    - All troponin elevations require a dose delay to allow for prompt cardiac evaluations. Following this evaluation, determination of further treatment will be based on the discussion with the sponsor or designee.

- Grade ≥2 non-skin, drug-related adverse events with the following exceptions:
    - Grade 2 drug-related fatigue or laboratory abnormalities do not require a treatment delay

- Any Grade 3 skin, drug-related AE

- Any Grade 3 drug-related laboratory abnormalities with the following exceptions for lymphopenia, leukopenia, AST, ALT, or total bilirubin
    - Grade 3 lymphopenia or leukopenia does not require dose delay
    - If a subject has a baseline AST, ALT, or total bilirubin that is within normal limits, delay dosing for drug-related Grade ≥2 toxicity

- If a subject has baseline AST, ALT, or total bilirubin within the Grade 1 toxicity range, delay dosing for drug-related Grade ≥3 toxicity

- Any adverse event, laboratory abnormalities, or intercurrent illness which in the judgment of the investigator, warrants delaying the dose of study medication.

  If dose delay is necessary, nivolumab (Arm A) or both nivolumab and ipilimumab (Arm B) must be delayed until treatment can resume.

  See current Investigator Brochure and Appendix 4 - Appendix 10 for citation examples.

  Dose delay criteria apply for all drug-related adverse events (regardless of whether or not the event is attributed to study drug). All study drugs must be delayed until treatment can resume.

  Subjects may resume treatment with study drug when the drug-related AE(s) resolve to Grade ≤ 1 or baseline value, with the following exceptions:

  - Subjects may resume treatment in the presence of Grade 2 fatigue
  - Subjects who have not experienced a Grade 3 drug-related skin AE may resume treatment in the presence of Grade 2 skin adverse event
  - Subjects with baseline Grade 1 AST/ALT or total bilirubin who require dose delays for reasons other than a 2-grade shift in AST/ALT or total bilirubin may resume treatment in the presence of Grade 2 AST/ALT OR total bilirubin
  - Subjects with combined Grade 2 AST/ALT AND total bilirubin values meeting discontinuation parameters should have treatment permanently discontinued
  - Drug-related pulmonary AEs, diarrhea, or colitis, must have resolved to baseline before treatment is resumed
  - Drug-related endocrinopathies adequately controlled with physiologic hormone replacement may resume treatment
  - In the neoadjuvant phase of therapy, if a treatment delay of more than three weeks is required, the next dose(s) can be omitted for patient safety and patient can stay on protocol as long as the patient is deemed to be responding clinically and will be able to proceed with surgery as planned.
  - If a subject in Arm B experiences a severe adverse event during neoadjuvant combination therapy that subsequently becomes well managed (grade 1), initiating treatment with nivolumab in the adjuvant setting may be considered if the patient is deemed fit to initiate adjuvant therapy within the appropriate treatment window (within 4-6 weeks of discharge from hospital for surgical excision).

- In the adjuvant phase of therapy: if the criteria to resume treatment are met, the subject should restart treatment at the next scheduled time-point per protocol.

  However, if the treatment is delayed past the next scheduled time-point per protocol, the next scheduled time-point will be delayed until dosing resumes. If treatment is delayed > 6 weeks, the subject must be permanently discontinued from study therapy, except as specified below

6.5.3 Study-Drug Discontinuation Criteria

Treatment should be permanently discontinued for the following:

- Grade 3 troponin not associated with any other sign of cardiac toxicity (as determined by a cardiac evaluation)
- Any Grade 3 non-skin, drug-related adverse event lasting > 7 days in the neoadjuvant and adjuvant settings, including uveitis, pneumonitis, colitis, and neurologic adverse events.
- Grade 3 drug-related laboratory abnormalities do not require treatment discontinuation except those noted below:
    - Grade 3 drug-related thrombocytopenia > 7 days or associated with bleeding requires discontinuation
    - Any drug-related liver function test (LFT) abnormality that meets the following criteria require discontinuation:
        - AST or ALT > 8 x ULN
        - Total bilirubin > 5 x ULN
        - Concurrent AST or ALT > 3 x ULN and total bilirubin > 2 x ULN
- Grade 3 myocarditis
- Any Grade 4 drug-related adverse event or laboratory abnormality, except for the following events which do not require discontinuation:
    - Isolated Grade 4 amylase or lipase abnormalities that are not associated with symptoms or clinical manifestations of pancreatitis and decrease to < Grade 4 within 1 week of onset.
    - Isolated Grade 4 electrolyte imbalances/abnormalities that are not associated with clinical sequelae and are corrected with supplementation/appropriate management within 72 hours of their onset
- Any dosing delay of more than 2 weeks in the neoadjuvant phase of treatment and > 6 weeks in the adjuvant phase of treatment with the following exceptions:

- Dosing interruptions to allow for prolonged steroid tapers to manage drug-related adverse events are allowed. Prior to re-initiating treatment in a subject with a dosing interruption lasting > 6 weeks, the Investigator must be consulted. Tumor assessments should continue as per protocol even if dosing is interrupted

- Dosing interruptions > 6 weeks that occur for non-drug-related reasons may be allowed if approved by the Investigator. Prior to re-initiating treatment in a subject with a dosing interruption lasting > 6 weeks, the Investigator must be consulted. Tumor assessments should continue as per protocol even if dosing is interrupted

- Any adverse event, laboratory abnormality, or intercurrent illness which, in the judgment of the Investigator, presents a substantial clinical risk to the subject with continued nivolumab dosing.

6.5.4 Treatment of Immunotherapy-Related Infusion Reactions

Since nivolumab, ipilimumab, and relatlimab contain only human immunoglobulin protein sequences, they are unlikely to be immunogenic and induce infusion or hypersensitivity reactions. However, if such a reaction were to occur, it might manifest with fever, chills, rigors, headache, rash, pruritus, arthralgias, hypo- or hypertension, bronchospasm, or other symptoms. All Grade 3 or 4 infusion reactions should be reported as an SAE if criteria are met. Infusion reactions should be graded according to NCI CTCAE (version 4.0) guidelines.

Treatment recommendations are provided below and may be modified based on local treatment standards and guidelines as appropriate:

For Grade 1 symptoms: (Mild reaction; infusion interruption not indicated; intervention not indicated) Remain at bedside and monitor subject until recovery from symptoms. The following prophylactic pre-medications are recommended for future infusions: diphenhydramine 50 mg (or equivalent) and/or paracetamol 325 to 1000 mg (acetaminophen) at least 30 minutes before additional nivolumab administrations.

For Grade 2 symptoms: (Moderate reaction requires therapy or infusion interruption but responds promptly to symptomatic treatment [e.g., antihistamines, non-steroidal anti-inflammatory drugs, narcotics, corticosteroids, bronchodilators, IV fluids]; prophylactic medications indicated for less than 24 hours). Stop the nivolumab or ipilimumab or relatlimab infusion, begin an IV infusion of normal saline, and treat the subject with diphenhydramine 50 mg IV (or equivalent) and/or paracetamol 325 to 1000 mg (acetaminophen); remain at bedside and monitor subject until resolution of symptoms. Corticosteroid or bronchodilator therapy may also be administered as appropriate. If the infusion is interrupted, then restart the infusion at 50% of the original infusion rate when symptoms resolve; if no further complications ensue after 30 minutes, the rate may be increased to 100% of the original infusion rate. Monitor subject closely. If symptoms recur then no further nivolumab or ipilimumab or relatlimab will be administered at that visit. Administer diphenhydramine 50 mg IV, and remain at bedside and monitor the subject until resolution of symptoms. The following prophylactic pre-medications are recommended for future infusions: diphenhydramine 50 mg (or equivalent) and/or paracetamol 325 to 1000 mg (acetaminophen) should be administered at least 30 minutes before additional

nivolumab or ipilimumab administrations. If necessary, corticosteroids (recommended dose: up to 25 mg of IV hydrocortisone or equivalent) may be used.

6.6 Permanent Discontinuation from Study Treatment and Subject Completion Criteria

6.6.1 Subject Completion Criteria

After subject has stopped receiving protocol therapy or if there is an intolerable effect, there will be an End of Treatment Visit. During this visit, a physical exam, blood about 6½ tablespoons will be drawn for routine tests and to test the immune system.

Every 12 weeks after the subject's last dose, a physical exam will be done. Six tablespoons of blood will be drawn for routine tests and to test the immune system; a CT scan, PET-CT scan, and/or MRI will be done to check the status of the disease.

A subject will be considered to have completed the study if the subject is no longer receiving clinical benefit (i.e., has progressive disease per RECIST, has died, has shown unacceptable toxicity, or study completion criteria have been met). A subject will be considered to have withdrawn from the study if the subject, in the absence of disease progression or death, is lost to follow-up, has withdrawn consent, or is withdrawn at the investigator's discretion. Patients enrolled will have completed the study after being followed for a minimum of two years after the time of surgical resection. Subjects who are ongoing at the time the study is closed/terminated will be considered to have completed the study.

6.6.2 Permanent Discontinuation from Study Treatment

During the protocol defined treatment period study treatment(s) may be permanently discontinued for the following reasons:

- Death
- Unacceptable adverse event
- Deviation(s) from the protocol
- Request of the subject or proxy
- Investigator's Discretion
- Subject is lost to follow-up
- Study is closed or terminated

The primary reason each study treatment was permanently discontinued must be documented in the subject's medical records.

If disease recurs prior to the completion of the treatment period, study treatment should be discontinued and follow-up assessments should be conducted every three months. Follow up assessments may be performed in person, by mail or via phone conversation.

If the subject voluntarily discontinues from treatment due to toxicity, 'adverse event' will be recorded as the primary reason for permanently discontinuation in the medical record.

All subjects who permanently discontinue from study treatment will have assessments at the time of discontinuation and during post study treatment follow- up as specified in Time and Events Tables. In addition, all subjects who permanently discontinue study treatment without evidence of disease recurrence will also be followed for disease recurrence according to the protocol schedule until:

- Withdrawal of consent
- Death, or
- Study completion (as defined in Section 7.5.1)

Subjects that permanently discontinue from study treatment before the end of the treatment period without evidence of disease recurrence will return for disease assessment visits starting at the next regularly scheduled disease assessment visit (i.e. every 12 weeks for restaging scans). If a subject experiences disease recurrence at any time, subsequent follow up should be conducted every three months in person, by mail or by phone contact.

Follow-up for survival, new anti-cancer therapy (including radiotherapy) and response to new anti-cancer therapy will continue for all subjects including those with disease recurrence for 2 years after the surgery after which all protocol- required assessments and procedures will be discontinued. Follow-up contact to assess survival and new anti- cancer therapy may be made via clinic visit or another form of communication (e.g. phone, email, mail etc.).

6.7 Prohibited Medications and Non-Drug Therapies

The use of illicit drugs within 28 days or 5 half-lives, whichever is shorter, prior to randomization and for the duration of the study will not be allowed.

The following medications or non-drug therapies are also prohibited while on treatment in this study:

- Other anti-cancer therapies;
- Other investigational drugs;
- Antiretroviral drugs (Note: Subjects with known HIV are ineligible for study participation);

Herbal remedies (e.g., St. John's wort)

7 Schedule of Assessments

7.1 Study Procedure Tables

Table 3 Study Procedure Table for Patients Randomized to Arm A (neoadjuvant and adjuvant nivolumab)

Procedures	**Screening** 28 Days	**Week 1** Day 1	**Week 3** Day 15 [h]	**Week 5** Day 29 [i]	**Week 7** Day 43 [j]	**Week 9** Day 57 [k]
Informed Consent	X					
Demographics	X					
Medical History	X					
Concurrent Medications	X	X	X	X	X	X
Adverse Events (AEs)	X	X	X	X	X	X
PE/Vitals/ECOG [a]	X	X	X	X	X	X
Pregnancy Test [b]	X					X
CBC with Diff	X	X	X	X	X	X
Serum Chemistry [c]	X	X	X	X	X	X
TSH, Free T4	X	X	X	X	X	
Coagulation	X					X
Hepatitis B and C	X					
12-Lead EKG	X					X
Biopsy [d]	X		X	X [l]		
Research Blood [e]	X		X	X		X
Disease Assessment [f]	X					X
Surgical Resection [g]						X
Nivolumab 3mg/kg		X	X [m]	X [m]	X [m]	

a. Systolic and diastolic blood pressure, pulse rate, and temperature. Must include full skin examination. Documentation of Eastern Cooperative Oncology Group status is required within 48 hours of each dose of drug administration
b. Serum or urine β-hCG - For women of childbearing potential only within 24 hours of start of study drugs and at week 9 visit
c. Albumin, alkaline phosphatase, total bilirubin, bicarbonate, BUN, calcium, chloride, creatinine, glucose, LDH, magnesium, phosphorus, potassium, total protein, AST, ALT, sodium. Amylase and lipase will be done if clinically indicated.
d. Minimal 5mm punch biopsy or core biopsy of safely accessible lesion. Additional biopsies will be performed around dose 2 (+/- 3 days) for patients in both treatment arms. When medically safe and feasible and subject agrees additional biopsies will be obtained around dose 3 (+/- 3 days) for both groups. Baseline biopsies are required for all enrolled patients. Week 5 biopsy is strongly encouraged but will be optional for patients with oligometastatic disease that is not safely amenable to serial biopsies
e. 40cc of blood will be obtained at each time point for immunologic and genomic correlative studies
f. PET/CT or CT of the chest, abdomen, pelvis (neck if clinically indicated) and brain MRI (or CT of the head with contrast if MRI is contraindicated)
g. Surgical resection of residual tumor if restaging scans show disease stability, favorable response to treatment, or progression of disease that is still deemed surgically resectable. A sample of the tumor will be obtained at the time of surgery to test for any residual melanoma cells and/or progression of disease.
h. Day 15 +/- 7 days
i. Day 29 +/- 7 days
j. Day 43 +/- 7 days
k. Day 57 +/- 7 days
l. Week 5 biopsy will be performed if subject has consented and if biopsy is deemed safe and feasible by study team
m. If a treatment delay of more than two weeks is required for patient safety, the next dose(s) can be omitted and patient can stay on protocol as long as the patient is deemed to be responding clinically and will be able to proceed with surgery as planned.

Table 4 Study Procedure Table for Patients Randomized to Arm B (neoadjuvant ipilimumab and nivolumab, adjuvant nivolumab)

Procedures	Screening 28 Days	Week 1 Day 1	Week 4 Day 22 [h]	Week 7 Day 43 [i]	Week 9 Day 57 [j]
Informed Consent	X				
Demographics	X				
Medical History	X				
Concurrent Medications	X	X	X	X	X
Adverse Events (AEs)	X	X	X	X	X
PE/Vitals/ECOG [a]	X	X	X	X	X
Pregnancy Test [b]	X				X
CBC with Diff	X	X	X	X	X
Serum Chemistry [c]	X	X	X	X	X
TSH, Free T4	X	X	X	X	
Coagulation	X				X
Hepatitis B and C	X				
12-Lead EKG	X				X
Biopsy [d]	X		X	X [k]	
Research Blood [e]	X		X	X	X
Disease Assessment [f]	X				X
Surgical Resection [g]					X
Nivolumab 1mg/kg		X	X [l]	X [l]	
Ipilimumab 3mg/kg		X	X	X	

a. Systolic and diastolic blood pressure, pulse rate, and temperature. Must include full skin examination. Documentation of Eastern Cooperative Oncology Group status is required within 48 hours of each dose of drug administration
b. Serum or urine β-hCG - For women of childbearing potential only within 24 hours of start of study drugs and at week 9 visit
c. Albumin, alkaline phosphatase, total bilirubin, bicarbonate, BUN, calcium, chloride, creatinine, glucose, LDH, magnesium, phosphorus, potassium, total protein, AST, ALT, sodium. Amylase and lipase will be done if clinically indicated.
d. Minimal 5mm punch biopsy or core biopsy of safely accessible lesion. Additional biopsies will be performed around dose 2 (+/- 3 days) for patients in both treatment arms. When medically safe and feasible and subject agrees additional biopsies will be obtained around dose 3 (+/- 3 days) for both groups. Baseline biopsies are required for all enrolled patients. Week 7 biopsy is strongly encouraged but will be optional for patients with oligometastatic disease that is not safely amenable to serial biopsies
e. 40cc of blood will be obtained at each time point for immunologic and genomic correlative studies
f. PET/CT or CT of the chest, abdomen, pelvis (neck if clinically indicated) and brain mri (or CT of the head with contrast if MRI is contraindicated)
g. Surgical resection of residual tumor if restaging scans show disease stability, favorable response to treatment, or progression of disease that is still deemed surgically resectable. A sample of the tumor will be obtained at the time of surgery to test for any residual melanoma cells and/or progression of disease.
h. Day 22 +/- 7 days
i. Day 43 +/- 7 days
j. Day 57 +/- 7 days
k. Week 7 biopsy will be performed if subject has consented and if biopsy is deemed safe and feasible by study team
l. If a treatment delay of more than two weeks is required for patient safety, the next dose(s) can be omitted and patient can stay on protocol as long as the patient is deemed to be responding clinically and will be able to proceed with surgery as planned.

Table 5 Study Procedure Table for Patients Enrolled to Arm C (neoadjuvant and adjuvant nivolumab and relatlimab)

Procedures	Screening 28 Days	Week 1	Week 3	Week 5	Week 9
		Day 1	Day 15 [h]	Day 29 [i]	Day 57 [j]
Informed Consent	X				
Demographics	X				
Medical History	X				
Concurrent Medications	X	X	X	X	X
Adverse Events (AEs)	X	X	X	X	X
PE/Vitals/ECOG [a]	X	X	X	X	X
Pregnancy Test [b]	X				X
CBC with Diff	X	X	X	X	X
Serum Chemistry [c]	X [m]	X	X	X	X
TSH, Free T4	X [m]	X	X	X	
Cardiac Troponin Level	X [m]		X	X	
Coagulation [o]	X				X
Hepatitis B and C	X				
12-Lead EKG	X				X
Echocardiogram [n]	X				
Biopsy [d]	X		X	X [k]	
Research Blood [e]	X		X	X	X
Disease Assessment [f]	X				X
Surgical Resection [g]					X
Nivolumab 480 mg and Relatlimab 160mg		X		X [l]	

a. Systolic and diastolic blood pressure, pulse rate, and temperature. Must include full skin examination. Documentation of Eastern Cooperative Oncology Group status is required within 48 hours of each dose of drug administration
b. Serum or urine β-hCG - For women of childbearing potential only within 24 hours of start of study drugs and at week 9 visit
c. Albumin, alkaline phosphatase, total bilirubin, bicarbonate, BUN, calcium, chloride, creatinine, glucose, LDH, magnesium, phosphorus, potassium, total protein, AST, ALT, sodium. Amylase and lipase will be done if clinically indicated.
d. Minimal 5mm punch biopsy or core biopsy of safely accessible lesion. Additional biopsies will be performed around day 15 (+/- 3 days) and day 29 (+/-3 days)
e. 40cc of blood will be obtained at each time point for immunologic and genomic correlative studies
f. PET/CT or CT of the chest, abdomen, pelvis (neck if clinically indicated) and brain mri (or CT of the head with contrast if MRI is contraindicated)
g. Surgical resection of residual tumor if restaging scans show disease stability, favorable response to treatment, or progression of disease that is still deemed surgically resectable. A sample of the tumor will be obtained at the time of surgery to test for any residual melanoma cells and/or progression of disease.
h. Day 15 +/- 7 days
i. Day 29 +/- 7 days
j. Day 57 +/- 7 days
k. Week 5 biopsy will be performed if subject has consented and if biopsy is deemed safe and feasible by study team
l. If a treatment delay of more than two weeks is required for patient safety, the next dose(s) can be omitted and patient can stay on protocol as long as the patient is deemed to be responding clinically and will be able to proceed with surgery as planned.
m. Subjects with controlled hyperthyroidism must be negative for thyroglobulin and thyroid peroxidase antibodies and thyroid-stimulating immunoglobulin. Subjects with Type 2 Diabetes must have HbA1c to establish baseline. Elevated troponin levels require cardiac evaluation.
n. Trans-thoracic echocardiogram (TTE) or Multigated Acquisition (MUGA) scan (TTE is preferred) performed within 6 months prior to initial study treatment.
o. Prothrombin time/International Normalized Ratio (PT/INR), Activated Partial thromboplastin time (aPTT).

Table 6 Adjuvant Therapy Study Procedure Table for Arm A and Arm B

Procedures	Week 10	Week 12	Week 14 [i]	Week 16	Week 18 [i]	Week 20	Week 22	Week 24 [i]	Week 26	Week 28 [i]	Week 30	Week 32 [i]	Week 34	Week 46, 58, 70, 82, 94, 106 [g]	EOT [h]
Concomitant Medications	X	X	X	X	X	X	X	X	X	X	X	X	X	X	X
Adverse Events (AEs)	X	X	X	X	X	X	X	X	X	X	X	X	X	X	X
PE/Vitals/ECOG [a]	X	X		X		X	X		X		X		X	X	X
CBC with Diff	X	X	X	X	X	X	X	X	X	X	X	X	X	X	X
Serum Chemistry [b]	X	X	X	X	X	X	X	X	X	X	X	X	X	X	X
TSH, Free T4	X		X		X		X		X		X		X	X	X
Research Blood [c]	X						X						X	X	X
Disease Assessment [d]		X					X						X	X	X
Nivolumab 3mg/kg [e]	X	X	X	X	X	X	X	X	X	X	X	X	X		
Biopsy [f]	If evidence of disease recurrence or metastasis														

a. Systolic and diastolic blood pressure, pulse rate, and temperature. Must include full skin examination. Documentation of Eastern Cooperative Oncology Group status
b. Albumin, alkaline phosphatase, total bilirubin, bicarbonate, BUN, calcium, chloride, creatinine, glucose, LDH, magnesium, phosphorus, potassium, total protein, AST, ALT, sodium. Amylase and lipase will be done if clinically indicated.
c. 40cc of blood will be obtained at each time point for immunologic and genomic correlative studies.
d. PET/CT or CT of the chest, abdomen, and pelvis are required but additional imaging of affected areas (neck or extremities) may be required for documentation of target lesions. Brain MRI (or CT of the head with contrast if MRI is contraindicated). MRI/CT imaging of the brain will be ordered but may be deferred in the setting of insurance company denial
e. Nivolumab treatment should resume within 4-6 weeks of discharge from the hospital for surgical excision. Patients will have a physical exam; blood will be drawn for routine tests and to test the immune system. Patients with involved margins after surgical excision will be allowed to initiate adjuvant therapy. Week 10 visit = start of adjuvant nivolumab treatment after recovery and wound healing post-surgical intervention.
f. Minimal 5mm punch biopsy or core biopsy of accessible lesion if there is disease recurrence or new metastasis. Biopsies at relapse are optional.
g. All time points are +/-7 days. Subjects will be followed every 12 weeks for 2 years after surgery For subjects who complete study treatment or subjects who discontinue study treatment early without disease recurrence, f/u will be done by clinic visit 12 months from last dosage (for PE, labs, research blood, restaging assessments etc). For subjects who discontinue study treatment early due to disease recurrence or subjects who cannot return to MDACC, f/u will be done by phone call, e -mail or letter etc. Follow-up for survival, new anti-cancer therapy and response to new anti-cancer therapy will continue for all subjects for 2 years after surgery.
h. End of treatment is defined as completion of 24 weeks (week 34 in the trial) of adjuvant nivolumab, experiencing SAE or intolerable AE mandating discontinuation of treatment or the development of recurrent disease
i. Patient to receive phone call to assess concomitant medications and AEs prior to infusion

Table 7 Adjuvant Therapy Study Procedure Table for Arm C

Procedures	Week 10 [g]	Week 14 [g]	Week 18 [g]	Week 22 [g]	Week 26 [g]	Week 30 [g]	Week 34 [g]	Week 38 [g]	Week 42 [g]	Week 46 [g, h]	Week 50 [g]	Week [g] 62, 74, 86, 98, 110	EOT
Concomitant Medications	X	X	X	X	X	X	X	X	X	X	X	X	X
Adverse Events (AEs)[i]	X	X	X	X	X	X	X	X	X	X	X	X	X
PE/Vitals/ECOG [a]	X	X	X	X	X	X	X	X	X	X	X	X	X
CBC with Diff	X	X	X	X	X	X	X	X	X	X	X	X	X
Serum Chemistry [b]	X	X	X	X	X	X	X	X	X	X	X	X	X
Pregnancy Test [k]	X			X			X			X		X	X
TSH, Free T4 [j]	X	X	X	X	X	X	X	X	X	X	X	X	X
Cardiac Troponin Level	X	X	X	X	X	X	X	X		X	X	X	X
Research Blood [c]	X	X			X			X		X	X	X	X
Disease Assessment [d]		X			X			X			X	X	X
Nivolumab 480 mg and Relatlimab 160 mg[e]	X	X	X	X	X	X	X	X	X	X			
Biopsy [f]	If evidence of disease recurrence or metastasis												

a. Systolic and diastolic blood pressure, pulse rate, and temperature. Must include full skin examination. Documentation of Eastern Cooperative Oncology Group status
b. Albumin, alkaline phosphatase, total bilirubin, bicarbonate, BUN, calcium, chloride, creatinine, glucose, LDH, magnesium, phosphorus, potassium, total protein, AST, ALT, sodium. Amylase and lipase will be done if clinically indicated.
c. 40cc of blood will be obtained at each time point for immunologic and genomic correlative studies. Performed every 12 weeks beginning with Week 14.
d. PET/CT or CT of the chest, abdomen, and pelvis are required but additional imaging of affected areas (neck or extremities) may be required for documentation of target lesions. Brain MRI (or CT of the head with contrast if MRI is contraindicated). MRI/CT imaging of the brain will be ordered but may be deferred in the setting of insurance company denial. Performed every 12 weeks beginning with Week 14. End of Treatment scans for disease assessment do not need to be repeated if scans were performed within 12 weeks.
e. Treatment should resume within 4-6 weeks of discharge from the hospital for surgical excision. Patients will have a physical exam; blood will be drawn for routine tests and to test the immune system. Patients with involved margins after surgical excision will be allowed to initiate adjuvant therapy. Week 10 visit = start of adjuvant treatment after recovery and wound healing post-surgical intervention. Administered every 4 weeks for 10 doses I the adjuvant setting.
f. Minimal 5mm punch biopsy or core biopsy of accessible lesion if there is disease recurrence or new metastasis. Biopsies at relapse are optional.
g. All time points are +/-7 days. Subjects will be followed every 12 weeks for 2 years after surgery For subjects who complete study treatment or subjects who discontinue study treatment early without disease recurrence, f/u will be done by clinic visit 12 weeks or 3 months from last dosage (for PE, labs, research blood, restaging assessments etc). For subjects who discontinue study treatment early due to disease recurrence or subjects who cannot return to MDACC, f/u will be done by phone call, e -mail or letter etc. Follow-up for survival, new anti-cancer therapy and response to new anti-cancer therapy will continue for all subjects for 2 years after surgery.
h. End of treatment is defined as completion of 10 doses of relatlimab (Week 46 in the trial) of adjuvant treatment, experiencing SAE or intolerable AE mandating discontinuation of treatment or the development of recurrent disease
i. Concomitant medications and AEs will be assessed. On days of infusion, assessment will occur prior to the infusion
j. Thyroid function testing will be performed every 4 weeks through Week 46 or EOT, whichever comes first. Thyroid function testing will be tested every 12 weeks in the Follow-Up phase through Week 110.
k. Pregnancy testing will be performed every 12 weeks through Week 110.

Every 12 weeks after the last dose of study drug, a complete physical exam will be done. The subject will also have blood drawn for routine tests and to test the immune system. A CT scan, PET-CT scan, or MRI will be done to check the status of the disease.

7.2 Critical Baseline Assessments

Baseline (Screening) assessments will be obtained within 28 days of treatment initiation and will include:

- Complete physical examination, including height (in cm) and weight (in kg)
- Vital signs: blood pressure, temperature, respiratory rate, pulse rate
- Eastern Cooperative Oncology Group (ECOG) performance status
- Clinical laboratory tests: hematology, clinical chemistry, and thyroid function, coagulation, and Hepatitis B and C. Cardiac troponin level will be assessed for patients in Arm C.
- Serum or urine beta-human chorionic gonadotropin (β-HCG) pregnancy test for female subjects of childbearing potential only
- 12-lead electrocardiogram (ECG)
- Trans-thoracic echocardiogram (TTE) for patients enrolled to Arm C
- Dermatologic exam by attending physician or mid-level provider
- Brain magnetic resonance imaging (MRI) with contrast or a computed tomography (CT) scan (with contrast) if MRI is contraindicated
- PET/CT or CT of the chest, abdomen and pelvis with contrast. CT of neck or extremity if clinically indicated
- Review of concomitant medications
- Assessment of baseline adverse events
- Biopsy of accessible tumor and acquisition of research blood for immune correlates

7.3 Safety Evaluations on Therapy

7.3.1 Physical Exam/ECOG/Vital Signs/Concomitant Medications

A complete physical examination will be performed by a qualified physician or a midlevel provider (physician's assistant, nurse practitioner). Documentation of Eastern Cooperative Oncology Group status is required within 48 hours of each dose of drug administration. Vital sign measurements will include systolic and diastolic blood pressure, pulse rate, and temperature. Assessment of concomitant medications will be obtained at each clinic visit.

7.3.2 Adverse Event Assessment

Assessment of adverse events will be performed by the research nurse, treating physician or mid-level provider.

7.3.3 Laboratory Assessments

Hematology, clinical chemistry, and additional parameters as per the Study Procedure Tables in Section 7.1 to be tested are listed below in Table 8 Required Laboratory Testing. Laboratory studies must be obtained within 48 hours of each time administration.

Table 8 Required Laboratory Testing

Hematology

Platelet Count	RBC Indices:	Automated WBC Differential:
WBC Count (absolute)	MCV	Neutrophils
Hemoglobin		Lymphocytes
Hematocrit		Monocytes
		Eosinophils
		Basophils

Clinical Chemistry

BUN	Potassium	AST (SGOT)	Total Bilirubin
Creatinine	Chloride	ALT (SGPT)	Total Protein
Glucose	Total CO_2	Lactate Dehydrogenase (LDH(	Albumin
Sodium	Calcium	Alkaline Phosphatase	Magnesium
Phosphorus			

Other Tests

β-hCG (urine or serum at screening for females of child bearing potential)
coagulation tests (PT/INR, PTT at screening and week 9)
TSH, free T4
Cardiac Troponin Levels

7.4 Biospecimen Collections

For blood and tissue correlative studies, specimens will be collected as part of the protocol as specified in the Study Procedure Tables (Section 7.1) and stored for later analysis.

Samples will be stored with the coded identification number or by tissue accession number. All samples and clinical data will be tracked by means of a unique research tracking number that will not be related to any patient identifying information. The link between research tracking numbers and patient identifiers will be kept in a secure application. More than one sample (each sample with a unique research tracking number) may be associated with a single medical record number in the database. Assays will be performed in research laboratories at MD Anderson, M.D. Anderson Immune Monitoring Core Facility and sub-investigating sites. Tumor tissue obtained for this protocol may be sent to the Parker Institute for Cancer Immunotherapy, Stanford University, Instituti Fisioterapici Ospitalieri – IRCCS – Regina Elena National Cancer Institute (IFO-IRE), National Cancer Institute, the University of Pittsburgh Medical Center, and the University of Washington for research analysis. Results of all assays and other collected data will be maintained in the secure research application.

Tissue will be procured from blood and tumor biopsies.

7.4.1 Peripheral Blood and Serum Samples

Blood processing for PBMC, plasma and storage: 30ml of venous peripheral blood will be collected in sodium heparin vacutainers, at the time points as described in Section 7 . 1 . The blood will be processed and stored, according to standard operating procedures, for PBMC, cell pellet and plasma for subsequent immune and molecular evaluation.

7.4.2 Tumor Biopsies and Surgical Specimen

All patients will have biopsies performed safely on accessible tumors before starting treatment (unless biopsy is not safe and tissue blocks are available for analysis). These biopsy samples should be excisional, incisional or core needle. Fine needle aspirates or other cytology specimens are insufficient for downstream biomarker analyses. The pre-treatment tumor biopsy must contain sufficient tumor content (≥100 tumor cells/4-micron tissue section) for PD-L1 immunohistochemistry testing prior to randomization in Arm A and B. Additional biopsies will be performed around dose 2 (+/- 7 days) for patients in Arms A and B. When medically safe and feasible, additional biopsies will be obtained around dose 3 (+/- 7 days) for Arms A and B. In addition, local surgery of isolated symptomatic lesions and biopsy prior to re-treatment in the setting of interrupted active treatment is strongly encouraged, if deemed clinically safe. For Arm C, tumor biopsy will be performed at baseline, day 15 (+/- 7 days) and day 29 (+/- 7 days. PD-L1 and LAG-3 testing will be performed on pre-treatment tumor biopsy but results are not needed prior to treatment initiation. Surgical resection samples for all groups will also be included for research analyses. In addition, any patient with accessible tumor at the time of relapse may have a tumor biopsy performed. Available paraffin embedded tumor tissue or excision may also be collected as pre-treatment comparator samples, so long as the patient provides consent for use in this study.

Preferred archival material includes tissue obtained in the same manner as specified in this trial and with no systemic anti-tumor treatment received by the patient between the biopsy and their enrollment on this protocol. However, other archival tissue may also be collected.

In order to accommodate all downstream biomarker analyses, incisional/excisional biopsies are strongly encouraged for this study, however, if incisional/excisional biopsies are not possible, core needle biopsies are acceptable as an alternative. Collection of a minimum of 4 core needle biopsies at least 10 mm in length using a needle gauge of 18 or larger will be used when feasible for all on treatment biopsies to accommodate PD-L1 staining and correlative research as indicated in the protocol.

For tissue obtained from MD Anderson participants, each set of core biopsies (up to four) or surgical specimen may be divided into portions and processed as below for later analysis. Specific instructions for processing may be provided in the event that four cores cannot be obtained.

1. FFPE for histology and IHC (25% of the specimen.)
2. 50% of the specimen in RPMI (Roswell Park Memorial Institute) media
3. 25% Snap frozen tumor in a cryovial

For tissue obtained from MSKCC participants, each set of core biopsies (up to four) or surgical specimen may be divided into portions and processed as below for later analysis. Specific instructions for processing may be provided in the event that four cores cannot be obtained.

1. 50% FFPE for histology and IHC
2. 50% snap frozen tumor in a cryovial

7.5 Disease Assessments

7.5.1 <u>Pathologic Assessments</u>

All patients will be evaluated with imaging after 7 weeks of neoadjuvant therapy prior to the planned surgical resection. Tumor samples will be collected within 28 days of treatment initiation, around dose 2 and dose 3 if feasible and medically safe and at the time of the surgical resection and all of these samples will be utilized for the primary endpoint analyses. All patients who experience a PR or SD will be eligible for surgical resection. Those patients who develop PD who no longer have disease amenable to gross total surgical resection will no longer be eligible for protocol therapy. Patients who initially had lymph node involvement but appear to have achieved a CR on imaging will proceed on to completion lymph node dissection. Patients with visceral metastases who appear to have achieved a CR on imaging may forgo any surgical procedure and will immediately initiate the adjuvant portion of the protocol.

Tumor specimens of patients undergoing evaluation for pathologic complete response (pCR), and pathologic partial response (pPR). pCR is defined as the absence of any residual

invasive malignant cells on hematoxylin and eosin evaluation of the resected melanoma specimen. pPR is defined as less than 50% viable tumor cells or more than 50% fibrosis on pathological evaluation.

7.5.2 Imaging Assessments

Disease assessment will include imaging (computed tomography, magnetic resonance imaging) and physical examination (as indicated for palpable/superficial lesions). At screening, an MRI or CT of the head with contrast is required. At minimum, PET/CT or CT of the chest, abdomen and pelvis are required but additional imaging of affected areas (neck or extremities) may be required for documentation of target lesions.

Disease assessment will be completed within 28 days prior to the first dose of study drug, then as indicated in the Study Procedure Tables (Section 7.1). More frequent disease assessments may be performed at the discretion of the investigator. To ensure comparability between baseline and subsequent assessments, the same method of assessment and the same technique will be used when assessing response.

Disease response will be recorded as complete response (CR), partial response (PR), stable disease (SD), or progressive disease (PD) according to RECIST 1.1 criteria (Appendix 2).

7.5.3 Translational Research/Biomarker Analysis

Serum samples may be assessed by such techniques as ELISA, seromics or other multiplex-based assay methods to measure soluble factors. Such factors likely will include, IFN-ɣ and interferon inducible factors, including CXCL9 and CXCL10, however, numerous serum-based biomarkers are currently under investigation for their potential to associate with efficacy to ipilimumab, nivolumab or other immunotherapy agents. These biomarkers include levels of soluble PD-L1, PD-1, anti-tumor antibodies, microRNAs (such as, but not limited to, miR-513, and miR19b), IL-12, TNFα, IL-10, TGF-β, VEGF, IL-6, IL-8, IL-17, IL-18, C-reactive protein and, as well as many other cytokines, chemokines, inflammatory factors and immune mediators. Such factors may be assessed in the context of the current study. Blood may also be collected at clinically relevant time points such as the occurrence of a ≥Grade 3 drug-related AEs.

PBMC samples may be used for immunophenotyping or characterization of the immune cell subsets in the periphery, including, but not limited to, T cells, B cells, NK cells, or subpopulations of the aforementioned immune cell types. These samples may also be used to assess immune cell function or antigen specific T cell proliferation or activation pending emerging information from other nivolumab or ipilimumab studies or sub-studies.

The MDACC Immunotherapy Platform (IMT) will perform immune monitoring of correlative samples collected at MD Anderson, including but not limited to evaluation of CD4 and CD8 T cells in peripheral blood (PBMCs) and available tumor samples as previously published. All samples that are provided to the IMT platform will be collected and analyzed as per a separate IRB-approved lab protocol.

Pre-treatment tumor tissue specimens in the form of a paraffin embedded block or a minimum of 3unstained slides, with a single section on positively charged slides will be submitted for PD-L1 immunohistochemistry (IHC) assessment.

Tumor samples may be assessed for the expression of other immune or melanoma related genes, RNAs and/or proteins, as well as, the presence of immune cell populations using a variety of methodologies inclusive of, but not limited to immunohistochemistry (IHC), qRT-PCR, genetic mutation detection and fluorescent in-situ hybridization (FISH). Various molecular markers with potential predictive value for the treatment of melanoma with ipilimumab and nivolumab, and other immunotherapies are currently under investigation and may be assessed in this study. These tumor tissue biomarkers include, but are not limited to PD-1, PD-L2, tumor infiltrating lymphocytes (TILs) or subpopulations of TILs and a Th1 immune mRNA expression signature.

Pending tissue availability other detailed analyses may include:

a) *Assess changes in the extent of T cell infiltrate over time.* FFPE tumor specimens will be stained for CD3, CD4, CD8, CD20, FoxP3 using commercial antibodies. Staining frequency for each antibody will be quantified and ratio of various T cell components (CD4/CD8, Treg/CD8 etc) will be calculated over time.

b) *Assess activation status of infiltrating T cells: Studies will include IHC and multiparameter flow cytometry:*

c) IHC: In the TIL infiltrate, cells staining for Ki67, granzyme B, IFN-γ, TGF-beta, GATA-3, RORgt, BCL2 will be enumerated to estimate T cell activation, and Th1/Th2/Th17 bias.

d) Flow cytometry: TIL isolated from serial tumor specimens will be assessed for expression of immunoregulatory and co-stimulatory markers PD1, CTLA4, LAG3, 41BB using available antibodies, and TCR zeta chain expression using flow. Baseline status and PBMC will serve as controls

e) *Assess via Nanostring for conversion to immune/inflammatory expression pattern*

f) RNA will be extracted from tumor tissue and analyzed for the presence or absence of an immune gene expression signature via our Nanostring codeset. Specimens will be run in duplicate. Previous positive and negative expressing tumors will be used as controls.

g) *Obtain pilot data on association between extent of TIL or other parameters with extent of tumor regression on week 8 tumor measurements and PFS.*

h) Patients will undergo tumor measurements pre-treatment and 9 weeks into therapy. Response will be calculated by RECIST 1.1. Percent tumor shrinkage relative to baseline will be analyzed. Extent of tumor shrinkage and individual patient PFS will be examined for association with measures of immune infiltration at various time points.

8 Safety and Reporting

The investigator and site staff will be responsible for detecting, documenting and reporting events that meet the definition of an adverse event (AE) or serious adverse event (SAE) as documented in Sections 8.1 and 8.2 respectively. Effective with approval of Amendment 7, this study will be a single arm trial, therefore the MD Anderson Data Safety Monitoring Board (MDACC DSMB) can be removed as the monitor.

Serious Adverse Event Reporting (SAE) for M. D. Anderson-sponsored Multicenter IND Protocols

An adverse event or suspected adverse reaction is considered "serious" if, in the view of either the investigator or the sponsor, it results in any of the following outcomes:

- Death
- A life-threatening adverse drug experience – any adverse experience that places the patient, in the view of the initial reporter, at immediate risk of death from the adverse experience as it occurred. It does not include an adverse experience that, had it occurred in a more severe form, might have caused death.
- Inpatient hospitalization or prolongation of existing hospitalization
- A persistent or significant incapacity or substantial disruption of the ability to conduct normal life functions.
- A congenital anomaly/birth defect.

Important medical events that may not result in death, be life-threatening, or require hospitalization may be considered a serious adverse drug experience when, based upon appropriate medical judgment, they may jeopardize the patient or subject and may require medical or surgical intervention to prevent one of the outcomes listed in this definition. Examples of such medical events include allergic bronchospasm requiring intensive treatment in an emergency room or at home, blood dyscrasias or convulsions that do not result in inpatient hospitalization, or the development of drug dependency or drug abuse (21 CFR 312.32).

- Important medical events as defined above, may also be considered serious adverse events. Any important medical event can and should be reported as an SAE if deemed appropriate by the Principal Investigator or the IND Sponsor, MDACC IND Office.
- All events occurring during the conduct of a protocol and meeting the definition of a SAE must be reported to the IRB in accordance with the timeframes and procedures outlined in "University of Texas M. D. Anderson Cancer Center Institutional Review Board Policy for Investigators on Reporting Unanticipated Adverse Events for Drugs and Devices".

  Unless stated otherwise in the protocol, all SAEs, expected or unexpected, must be reported to the IND Office, regardless of attribution (within 5 working days of knowledge of the event).

Reporting for all sites:

- A written report should be submitted to the Institutional Review Board (IRB) according to the requirements of the assigned IRB for patients enrolled at each site. (The MD Anderson site will utilize the electronic SAE application (eSAE) for reporting SAEs.)

- SAEs will be reported to the sponsor on a template form that will be provided to each site. If needed, a copy of all relevant examinations that have been carried out and the dates on which these examinations were performed should be attached. For laboratory results, normal ranges should be included. Patient name should be marked out and initials and study number included on all attachments.

- In case of a serious adverse event, the following actions must be undertaken by the investigator: (Please note that these are in addition to reporting that is required by the local IRB and supporting company.) Complete the SAE form immediately and then fax and overnight mail the signed and dated SAE form to the sponsor representative within two working days to the following address:

  The University of Texas M.D. Anderson Cancer Center
  IND Office
  Georgina Melendez. Proj. Mgr., Medical Affairs and Safety
  IND Office
  7007 Bertner, 1MC12.2227
  Houston, Texas 77030
  Tel no.: 713-563-8772
  Fax no.: 713-563-5468
  e-mail: mdaccsafetyreports@mdanderson.org

- A copy of the tracking receipt should be kept and filed in the study regulatory binder at the site. The research team may e-mail or call the sponsor to confirm receipt of the SAE fax or mailed form.

- ***Death or life-threatening events that are possibly, probably or definitely related to drug must be reported within 24 hours***. The sponsor IND safety coordinator must be notified by phone immediately, in addition to fax or overnight mail as listed above.

- **All life-threatening or fatal events,** expected or unexpected, and regardless of attribution to the study drug, must have a written report submitted within **24 hours** (next working day) of knowledge of the event to the Safety Project Manager in the IND Office.

- **Serious adverse events will be captured from the time of the first protocol-specific intervention, unless the protocol states otherwise, and be reported until 30 days after the last dose of drug. Serious adverse events must be followed until clinical recovery is complete and laboratory tests have returned to baseline, progression of the event has stabilized, or there has been acceptable resolution of the event.**

- **Additionally, any serious adverse events that occur after the 30 day time period that are related to the study treatment must be reported to the IND Office. This may include the development of a secondary malignancy.**

- **All events reported the supporting company must also be report to the IND Office.**

Reporting to FDA:

- Serious adverse events will be forwarded to FDA by the IND Sponsor (Safety Project Manager IND Office) according to 21 CFR 312.32.

It is the responsibility of the PI and the research team to ensure serious adverse events are reported according to the Code of Federal Regulations, Good Clinical Practices, the protocol guidelines, the sponsor's guidelines, and Institutional Review Board policy.

Investigator Communication with Supporting Companies:

8.1 Supporter SAE Reporting

- All Serious Adverse Events (SAEs) that occur following the subject's written consent to participate in the study through 100 days of discontinuation of dosing must be reported to BMS Worldwide Safety.

- If the BMS safety address is not included in the protocol document (e.g. multicenter studies where events are reported centrally), the procedure for safety reporting must be reviewed/approved by the BMS Protocol Manager. Procedures for such reporting must be reviewed and approved by BMS prior to study activation.

- The BMS SAE form should be used to report SAEs. If the BMS form cannot be used, another acceptable form (i.e CIOMS or Medwatch) must be reviewed and approved by BMS. The BMS protocol ID number must be included on whatever form is submitted by the Sponsor/Investigator.

- Following the subject's written consent to participate in the study, all SAEs, whether related or not related to study drug, are collected, including those thought to be associated with protocol-specified procedures. The investigator should report any SAE occurring after these time periods that is believed to be related to study drug or protocol-specified procedure.

- The investigator will request from BMS GPV&E, aepbusinessprocess@bms.com the SAE reconciliation report and include the BMS protocol number every 3 months

- GPV&E will send the investigator the report to verify and confirm all SAEs have been transmitted to BMS GPV&E.

- The data elements listed on the GPV&E reconciliation report will be used for case identification purposes. If the Investigator determines a case was not transmitted to BMS GPV&E, the case should be sent immediately to BMS (Worldwide.Safety@bms.com)

- In accordance with local regulations, BMS will notify investigators of all reported SAEs that are suspected (related to the investigational product) and unexpected (i.e., not previously described in the IB). In the European Union (EU), an event meeting these criteria is termed a Suspected, Unexpected Serious Adverse Reaction (SUSAR). Investigator notification of these events will be in the form of an expedited safety report (ESR).
    - Other important findings which may be reported by the as an ESR include: increased frequency of a clinically significant expected SAE, an SAE considered associated with study procedures that could modify the conduct of the study, lack of efficacy that poses significant hazard to study subjects, clinically significant safety finding from a nonclinical (e.g., animal) study, important safety recommendations from a study data monitoring committee, or sponsor decision to end or temporarily halt a clinical study for safety reasons.
    - Upon receiving an ESR from BMS, the investigator must review and retain the ESR with the IB. Where required by local regulations or when there is a central IRB/IEC for the study, the sponsor will submit the ESR to the appropriate IRB/IEC. The investigator and IRB/IEC will determine if the informed consent requires revision. The investigator should also comply with the IRB/IEC procedures for reporting any other safety information.
    - In addition, suspected serious adverse reactions (whether expected or unexpected) shall be reported by BMS to the relevant competent health authorities in all concerned countries according to local regulations (either as expedited and/or in aggregate reports).

Serious Adverse Event Collection and Reporting

Following the subject's written consent to participate in the study, all SAEs, whether related or not related to study drug, must be collected, including those thought to be associated with protocol-specified procedures. All SAEs must be collected that occur within 100 days of discontinuation of dosing.

All SAEs must be collected that occur during the screening period. If applicable, SAEs must be collected that relate to any protocol-specified procedure (e.g., a follow-up skin biopsy). The investigator should report any SAE that occurs after these time periods that is believed to be related to study drug or protocol-specified procedure.

SAEs, whether related or not related to study drug, and pregnancies must be reported to BMS within 24 hours. SAEs must be recorded on BMS or an approved form; pregnancies on a Pregnancy Surveillance Form.

SAE Email Address: Worldwide.Safety@BMS.com

SAE Facsimile Number: 609-818-3804

If only limited information is initially available, follow-up reports are required. (Note: Follow-up SAE reports should include the same investigator term(s) initially reported.)

If an ongoing SAE changes in its intensity or relationship to study drug or if new information becomes available, a follow-up SAE report should be sent within 24 hours to the BMS (or designee) using the same procedure used for transmitting the initial SAE report.

All SAEs should be followed to resolution or stabilization.

For studies conducted under an Investigator IND in the US include the following:

For studies conducted under an Investigator IND in the US, any event that is both serious and unexpected must be reported to the Food and Drug Administration (FDA) as soon as possible and no later than 7 days (for a death or life-threatening event) or 15 days (for all other SAEs) after the investigator's or institution's initial receipt of the information. BMS will be provided with a simultaneous copy of all adverse events filed with the FDA.

SAEs should be reported on MedWatch Form 3500A, which can be accessed at: http://www.accessdata.fda.gov/scripts/medwatch/

MedWatch SAE forms should be sent to the FDA at:

MEDWATCH
5600 Fishers Lane
Rockville, MD 20852-9787
Fax: 1-800-FDA-0178 (1-800-332-0178)
http://www.accessdata.fda.gov/scripts/medwatch/

All SAEs should simultaneously be faxed or e-mailed to BMS at:

Global Pharmacovigilance & Epidemiology
Bristol-Myers Squibb Company
Fax Number: 609-818-3804
Email: Worldwide.safety@bms.com

- An SAE report should be completed for any event where doubt exists regarding its seriousness.
- For studies with long-term follow-up periods in which safety data are being reported, include the timing of SAE collection in the protocol.
- If the investigator believes that an SAE is not related to study drug, but is potentially related to the conditions of the study (such as withdrawal of previous therapy or a complication of a study procedure), the relationship should be specified in the narrative section of the SAE Report Form.
- If only limited information is initially available, follow-up reports are required. (Note: Follow-up SAE reports should include the same investigator term(s) initially reported.)
- If an ongoing SAE changes in its intensity or relationship to study drug or if new information becomes available, a follow-up SAE report should be sent within 24 hours to BMS using the same procedure used for transmitting the initial SAE report. All SAEs should be followed to resolution or stabilization. All SAEs should be followed to resolution or stabilization.

8.2 Non-Serious Adverse Event Collection and Reporting

The collection of non-serious AE information should begin at initiation of study drug. All non-serious adverse events (not only those deemed to be treatment-related) should be collected continuously during the treatment period and for a minimum of 30 days following the last dose of study treatment.

Non-serious AEs should be followed to resolution or stabilization, or reported as SAEs if they become serious. Follow-up is also required for non-serious AEs that cause interruption or discontinuation of study drug and for those present at the end of study treatment as appropriate.

8.3 Laboratory Test Abnormalities

All laboratory test results captured as part of the study should be recorded following institutional procedures. Test results that constitute SAEs should be documented and reported as such.

The following laboratory abnormalities should be documented and reported appropriately:

- any laboratory test result that is clinically significant or meets the definition of an SAE
- any laboratory abnormality that required the subject to have study drug discontinued or interrupted
- any laboratory abnormality that required the subject to receive specific corrective therapy.
- Wherever possible, timely confirmation of initial liver-related laboratory abnormalities should occur prior to the reporting of a potential DILI event. All occurrences of potential DILIs, meeting the defined criteria, must be reported as SAEs.
    - Potential drug induced liver injury is defined as:
        1. AT (ALT or AST) elevation > 3 times upper limit of normal (ULN)

           AND

        2. Total bilirubin > 2 times ULN, without initial findings of cholestasis (elevated serum alkaline phosphatase)

           AND

        3. No other immediately apparent possible causes of AT eleation and hyperbilirubinemia, including, but not limited to, viral hepatitis, pre-existing chronic or acute live disease, or the administration of other drug(s) known to be hepatoxic.

8.4 Pregnancy

If, following initiation of the investigational product, it is subsequently discovered that a study subject is pregnant or may have been pregnant at the time of investigational product exposure, including during at least 6 half-lives after product administration, the investigational product will be permanently discontinued in an appropriate manner (e.g., dose tapering if necessary for subject safety).

The investigator must immediately notify Worldwide Safety @BMS of this event via the Pregnancy Surveillance Form in accordance with SAE reporting procedures.

Follow-up information regarding the course of the pregnancy, including perinatal and neonatal outcome and, where applicable, offspring information must be reported on the Pregnancy Surveillance Form [provided upon request from BMS]

Any pregnancy that occurs in a female partner of a male study participant should be reported to BMS. Information on this pregnancy will be collected on the Pregnancy Surveillance Form.

8.5 Overdose

An overdose is defined as the accidental or intentional administration of any dose of a product that is considered both excessive and medically important. All occurrences of overdose must be reported as an SAE.

8.6 Other Safety Considerations

Any significant worsening noted during interim or final physical examinations, electrocardiograms, x-rays, and any other potential safety assessments, whether or not these procedures are required by the protocol, should also be recorded as a nonserious or serious AE, as appropriate, and reported accordingly.

9 Statistical Considerations

9.1 Sample Size and Power

Arm A and Arm B

Patients with, stage IIIB/IIIC or stage IV resectable, oligometastatic melanoma will be enrolled in this phase 2 study. Patients with prior adjuvant therapy including use of prior interferon alpha, pegylated interferon or vaccine will be eligible after a 28 day washout period. This is an exploratory biomarker study and no statistical comparisons will be made between the two treatment arms. The primary objective is to assess pathologic response of neoadjuvant nivolumab monotherapy (Arm A) and nivolumab and ipilimumab combination therapy (Arm B). pCR is defined as the absence of any residual invasive malignant cells on hematoxylin and eosin evaluation of the resected melanoma specimen. pPR is defined as less than 50% viable tumor cells or more than 50% fibrosis on pathological evaluation.

We assume the pathologic complete response rate will be 5% for patients in Arm A and 15% for Arm B. These data are extrapolated from the imaging CR rates from previously published data. [17, 18, 62, 63, 66] Thus, assuming the true pathologic complete response rate in Arm A is 5%, the probability of at least 1 out of 20 patients experiencing a response is 0.64. For treatment Arm B, the probability of at least 1 out of 20 patients experiencing a pathologic response is 0.96 assuming a true response rate of 15%.

Arm C

Thirty patients will be enrolled to Arm C.. The primary objective is to assess the pathologic response rate of neoadjuvant relatlimab with nivolumab. Pathologic complete response is defined as the absence of any residual invasive malignant cells on hematoxylin and eosin evaluation of the resected melanoma surgical specimen. Pathologic partial response is defined as less than 50% viable tumor cells or more than 50% fibrosis on pathologic evaluation of the surgical sample. All tumor samples are evaluated by a single dermatopathology certified collaborator.

We assume the pathologic response rate will be 30% for patients in Arm C. Assuming this true pathologic response rate, the probability of at least 5 out of 30 patients experiencing a response is 0.97.

9.2 Study Populations

Intent-to-treat (ITT) population: All patients randomized to Arm A or B, and treated in Arm C in the study will be included in the analysis regardless of their adherence to entry criteria, treatment they actually received, compliance, and subsequent withdrawal from treatment or protocol deviation.

9.2.1 Safety Population

All patients who receive at least one dose of study treatment will be evaluable from the time of their first treatment.

9.3 Stopping Rule

Continuous monitoring of disease progression will be performed for all patients in treatment Arm A and Arm B, beginning with the first four patients. Assuming a prior beta distribution of (0.4, 1.6), the treatment arm will terminate if the Pr ($\delta_{dp} > 0.20$ | data) > 0.85, where δ_{dp} is the proportion of patients experiencing disease progression within the treatment arm. Disease progression is defined as progression such that disease is no longer resectable based on week 9 imaging. The decision rule for discontinuing enrollment in a specific treatment arm is presented in Table 9 and the operating characteristics for this rule are presented in Table 10.

Continuous monitoring of toxicity and futility will be performed for all patients in Arm C, beginning with the first six patients. Toxicity/efficacy summaries will be provided to the IND Office every 6 subjects. Assuming a prior beta distribution of (1, 1), corresponding to a toxicity

rate of 50%, the treatment arm will terminate if the Pr ($\delta_T > 0.50$ | data) > 0.975, where δ_T is the proportion of patients experiencing ≥ grade 3 toxicities. For futility, a prior beta distribution of (0.6, 1.4) will be assumed, corresponding to a pRR of 30%. Patient enrollment will terminate if the Pr ($\delta_{pRR} < 0.30$ | data) > 0.975, where δ_{pRR} is the pRR attributable to the treatment combination. The decision rule for terminating for toxicity is presented in Table 11 while the decision rule for terminating for futility is presented in Table 12 and the operating characteristics for these rules are presented in Table 13. The method used to produce the decision rules and operating characteristics was designed by Thall, Simon, and Estey[72,73] as extended by Thall and Sung[74].

Table 9 Stopping Boundaries for Arm A and Arm B

Total number of patients	Stop enrollment in treatment arm if there are this many patients with disease progression
1-3	Never stop with this many patients
4-7	3-7
8-11	4-11
12-15	5-15
16-19	6-19
20	Always stop with this many patients

Table 10 Operating Characteristics for Arm A and Arm B

True % of DLTs	Pr (stopping early)	Median Sample Size (interquartile)
0.05	0.0	20 (20, 20)
0.10	0.0	20 (20, 20)
0.20	0.2	20 (17, 20)
0.30	0.6	13 (6, 20)
0.40	0.8	7 (5, 13)
0.60	1.0	5 (4, 6)

Table 11 Stopping Criteria - Toxicity for Arm C

Total number of patients	Stop enrollment in treatment Arm C if there are this many patients with ≥ grade 3 toxicities
1-5	Never stop with this many patients
6	6
7-8	7-8
9	8-9
10-11	9-11
12	10-12
13-14	11-14
15-16	12-16
17	13-17
18-19	14-19
20	15-20
21-22	16-22
23-24	17-24
25	18-25
26-27	19-27
28-29	20-29
30	End of Enrollment

Table 12 Stopping Criteria - Futility for Arm C

Total number of patients	Stop enrollment if there are this many patients responding
1-6	Never stop with this many patients
7-12	0
13-18	0-1
19-23	0-2
24-27	0-3
28-29	0-4
30	End of Enrollment

Table 13 Operating Characteristics for Arm C

True Toxicity Rate	True pRR	Pr (stopping early)	Average # of Patients Treated
0.20	0.10	0.90	14
	0.20	0.46	23
	0.30	0.14	27
	0.40	0.04	29
	0.60	0.002	30
0.30	0.10	0.90	14
	0.20	0.46	23
	0.30	0.15	27
	0.40	0.04	29
	0.60	0.004	30
0.50	0.10	0.91	14
	0.20	0.51	21
	0.30	0.23	26
	0.40	0.13	28
	0.60	0.10	28
0.60	0.10	0.94	13
	0.20	0.64	19
	0.30	0.44	23
	0.40	0.37	24
	0.60	0.34	25
0.80	0.10	1.00	9
	0.20	0.98	10
	0.30	0.97	11
	0.40	0.97	12
	0.60	0.97	12

9.4 Patient Characteristics

Demographic and baseline characteristics of the ITT population will be summarized for all patients and by treatment arm. Categorical measures will be summarized using frequencies and percentages while continuous variables will be summarized using mean, standard deviation, median, minimum, and maximum.

9.5 Primary Efficacy Measure

The primary efficacy measure in this study is pathologic response. The proportion of patients experiencing pathologic response will be computed with associated 95% CI for each treatment arm.

9.6 Secondary Efficacy Measures

9.6.1 Immunologic Response

Immunologic response will be assessed by change in T cell infiltrate from baseline to each study procedure visit (approximately 9 weeks). The change in T cell infiltrate will be assessed over time for each treatment arm using a generalized linear mixed model with terms for visit, stage of disease, and PD-L1 tumor status.

9.6.2 Objective Response

Objective response (i.e., CR, PR, SD, and PD) will be categorized based on criteria specified in Appendix 2. The proportion of patients experiencing objective response will be computed with associated 95% CI for each treatment arm.

9.6.3 Recurrence-Free and Overall Survival

Recurrence-free survival (RFS) will be defined from time of surgical resection to the date of documented disease recurrence. Patients who are still alive without disease progression after 12 months of treatment start date will be censored. Overall survival (OS) will be defined from treatment start date to date of death. Patients who are still alive after 12 months of treatment start date will be censored. RFS and OS will be estimated using the Kaplan-Meier method for each treatment arm.

9.7 Exploratory Efficacy Measures

Various molecular markers with potential predictive value for the treatment of melanoma may be assessed. The association between change in tumor size and immune infiltration within each treatment arm will be assessed using a generalized linear mixed model where change in tumor size will be the dependent variable and gene expression, visit, and gene expression-by-visit interaction will be the independent variables. The association between RFS and immune infiltration within each treatment arm will be determined by a Cox proportional hazards regression model.

9.8 Safety Measures

Safety and tolerability within each treatment arm will be assessed by vital signs, laboratory assessments, adverse events, and serious adverse events for the safety population. Adverse events will be graded by the CTCAE version 4.0. Categorical measures will be summarized using frequencies and percentages while continuous variables will be summarized using mean, standard deviation, median, minimum, and maximum.

10 Study Management

10.1 Data Management

10.1.1 Enrollment

For the purposes of this study at M. D. Anderson Cancer Center, the Protocol Data Management System (PDMS) will be employed. All patients will be registered in the Clinical Oncology Research system (CORe) before any study specific tests are performed. Registration in CORe will be performed by MDACC study staff after eligibility is confirmed for each potential subject at either site.

10.1.2 Data Entry and Maintenance

Data for this protocol will be entered into a secure electronic data capture (EDC), such as Prometheus. All participating sites will use this EDC. The Parker Institute, Dana Farber Cancer Institute, Instiut Gustave Roussy, Stanford, Instituti Fisioterapici Ospitalieri – IRCCS – Regin Elena National Cancer Institute (IFO-IRE), National Cancer Institute, the University of Pittsburgh Medical Center, and the University of Washington will also have access to the study data for data analysis purposes.

The investigator is required to retain, in a confidential manner, the data pertinent to the study for the duration of the study or the maximum period required by applicable regulations and guidelines or institutional procedures. If the investigator withdraws from the study (e.g., relocation, retirement), the records shall be transferred to a mutually agreed upon designee (e.g., another investigator or IRB).

The concomitant medications will not be captured in the case report form (PDMS). They will be captured in the subject's medical records.

Posting of Information on Clinicaltrials.gov

Study information from this protocol will be posted on clinicaltrials.gov before enrollment of subjects begins.

10.1.3 Records Retention

Following closure of the study, the investigator must maintain all site study records (except for those required by local regulations to be maintained elsewhere) in a safe and secure location. The records must be easily accessible when needed and must be available for review in conjunction with assessment of the facility, supporting systems, and relevant site staff.

10.2 Study Monitoring

The University of Texas MD Anderson Cancer Center IND office will monitor the study investigators to assure data recording, and protocol adherence. The site principal investigator and staff are expected to cooperate and provide all relevant study documentation in detail at each visit on request for review. MD Anderson Cancer Center will monitor and/or audit the other participating sites to assure satisfactory protocol adherence and enrollment. The site will be visited on a regular basis by the Clinical Study Monitor, who will check completed

source documentation, discuss the progress of the study and monitor drug according to good clinical practice (GCP). The monitoring will also include source data verification (SDV).

11 References

1. American Cancer Society. What are the key statistics about melanoma skin cancer? *cancer.org* (2014). at <http://www.cancer.org/cancer/skincancer-melanoma/detailedguide/melanoma-skin-cancer-key-statistics>

2. World Health Organization. Health effects of UV radiation. *WHO* (2014).

3. Balch, C. M. *et al.* Final Version of 2009 AJCC Melanoma Staging and Classification. *Journal of Clinical Oncology* **27,** 6199–6206 (2009).

4. Tsao, H., Atkins, M. B. & Sober, A. J. Management of cutaneous melanoma. *N Engl J Med* **351,** 998–1012 (2004).

5. Hancock, B. W. *et al.* Adjuvant interferon in high-risk melanoma: the AIM HIGH Study-United Kingdom Coordinating Committee on Cancer Research randomized study of adjuvant low-dose extended-duration interferon Alfa-2a in high-risk resected malignant melanoma. *J Clin Oncol* **22,** 53–61 (2004).

6. Eggermont, A. M. *et al.* Adjuvant therapy with pegylated interferon alfa-2b versus observation alone in resected stage III melanoma: final results of EORTC 18991, a randomised phase III trial. *The Lancet* **372,** 117–126 (2008).

7. Grob, J. J. *et al.* Randomised trial of interferon alpha-2a as adjuvant therapy in resected primary melanoma thicker than 1.5 mm without clinically detectable node metastases. French Cooperative Group on Melanoma. *Lancet* **351,** 1905–1910 (1998).

8. Kirkwood, J. M. *et al.* Interferon alfa-2b adjuvant therapy of high-risk resected cutaneous melanoma: the Eastern Cooperative Oncology Group Trial EST 1684. *J Clin Oncol* **14,** 7–17 (1996).

9. Kirkwood, J. M. *et al.* High- and low-dose interferon alfa-2b in high-risk melanoma: first analysis of intergroup trial E1690/S9111/C9190. *J Clin Oncol* **18,** 2444–2458 (2000).

10. Kirkwood, J. M. *et al.* A pooled analysis of eastern cooperative oncology group and intergroup trials of adjuvant high-dose interferon for melanoma. *Clin. Cancer Res.* **10,** 1670–1677 (2004).

11. Atkins, M. B. *et al.* High-dose recombinant interleukin 2 therapy for patients with metastatic melanoma: analysis of 270 patients treated between 1985 and 1993. *J Clin Oncol* **17,** 2105–2116 (1999).

12. Chapman, P. B. *et al.* Improved survival with vemurafenib in melanoma with BRAF V600E mutation. *N Engl J Med* **364,** 2507–2516 (2011).

13. Hauschild, A. *et al.* Dabrafenib in BRAF-mutated metastatic melanoma: a multicentre, open-label, phase 3 randomised controlled trial. *Lancet* **380,** 358–365

(2012).

14. Flaherty, K. T. *et al.* Improved Survival with MEK Inhibition in BRAF-Mutated Melanoma. *N Engl J Med* **367,** 107–114 (2012).

15. Hodi, F. S. *et al.* Improved Survival with Ipilimumab in Patients with Metastatic Melanoma. *N Engl J Med* **363,** 711–723 (2010).

16. Robert, C. *et al.* Ipilimumab plus dacarbazine for previously untreated metastatic melanoma. *N Engl J Med* **364,** 2517–2526 (2011).

17. Topalian, S. L. *et al.* Safety, Activity, and Immune Correlates of Anti–PD-1 Antibody in Cancer. *N Engl J Med* **366,** 2443–2454 (2012).

18. Nivolumab Shows Survival Benefit in Melanoma. *medscape.com* at <http://www.medscape.com/viewarticle/827405>

19. Brunet, J. F. *et al.* A new member of the immunoglobulin superfamily--CTLA-4. *Nature* **328,** 267–270 (1987)

20. Mocellin, S., Benna, C. & Pilati, P. Coinhibitory molecules in cancer biology and therapy. *Cytokine and Growth Factor Reviews* 1–15 (2013). doi:10.1016/j.cytogfr.2013.01.003

21. Sansom, D. M. & Walker, L. S. K. The role of CD28 and cytotoxic T-lymphocyte antigen-4 (CTLA-4) in regulatory T-cell biology. *Immunol Rev* **212,** 131–148 (2006).

22. Walunas, T. L. *et al.* CTLA-4 can function as a negative regulator of T cell activation. *Immunity* **1,** 405–413 (1994).

23. Kearney, E. R. *et al.* Antigen-dependent clonal expansion of a trace population of antigen-specific CD4+ T cells in vivo is dependent on CD28 costimulation and inhibited by CTLA-4. *J Immunol* **155,** 1032–1036 (1995).

24. Yang, Y. F. *et al.* Enhanced induction of antitumor T-cell responses by cytotoxic T lymphocyte-associated molecule-4 blockade: the effect is manifested only at the restricted tumor-bearing stages. *Cancer Research* **57,** 4036–4041 (1997).

25. Grosso, J. F. & Jure-Kunkel, M. N. CTLA-4 blockade in tumor models: an overview of preclinical and translational research. *Cancer Immun* **13,** 5 (2013).

26. Hodi, F. S. *et al.* Biologic activity of cytotoxic T lymphocyte-associated antigen 4 antibody blockade in previously vaccinated metastatic melanoma and ovarian carcinoma patients. *Proc Natl Acad Sci USA* **100,** 4712–4717 (2003).

27. Downey, S. G. *et al.* Prognostic factors related to clinical response in patients with metastatic melanoma treated by CTL-associated antigen-4 blockade. *Clin. Cancer Res.* **13,** 6681–6688 (2007).

28. Hoos, A. *et al.* Development of Ipilimumab: Contribution to a New Paradigm for Cancer Immunotherapy. *YSONC* **37,** 533–546 (2010).

29. O'Day, S. J. *et al.* Efficacy and safety of ipilimumab monotherapy in patients with pretreated advanced melanoma: a multicenter single-arm phase II study. *Annals of Oncology* **21,** 1712–1717 (2010).

30. Wolchok, J. D. *et al.* Ipilimumab monotherapy in patients with pretreated advanced melanoma: a randomised, double-blind, multicentre, phase 2, dose-ranging study. *The Lancet Oncology* **11,** 155–164 (2010).

31. Robert, C. *et al.* Efficacy and safety of retreatment with ipilimumab in patients with pretreated advanced melanoma who progressed after initially achieving disease control. *Clin. Cancer Res.* **19,** 2232–2239 (2013).

32. Hoos, A. *et al.* Improved endpoints for cancer immunotherapy trials. *JNCI Journal of the National Cancer Institute* **102,** 1388–1397 (2010).

33. Wolchok, J. D. *et al.* Guidelines for the evaluation of immune therapy activity in solid tumors: immune-related response criteria. *Clin. Cancer Res.* **15,** 7412–7420 (2009).

34. Eggermont, A. M. M. *et al.* Ipilimumab versus placebo after complete resection of stage III melanoma: Initial efficacy and safety results from the EORTC 18071 phase III trial. *J Clin Oncol* **32,** abst LBA9008 (2014).

35. Weber, J. Immune Checkpoint Proteins: A New Therapeutic Paradigm for Cancer—Preclinical Background: CTLA-4 and PD-1 Blockade. *YSONC* **37,** 430–439 (2010).

36. Terme, M. *et al.* IL-18 induces PD-1-dependent immunosuppression in cancer. *Cancer Research* **71,** 5393–5399 (2011).

37. Fanoni, D. *et al.* New monoclonal antibodies against B-cell antigens: possible new strategies for diagnosis of primary cutaneous B-cell lymphomas. *Immunol. Lett.* **134,** 157–160 (2011).

38. Nishimura, H. *et al.* Developmentally regulated expression of the PD-1 protein on the surface of double-negative (CD4-CD8-) thymocytes. *Int. Immunol.* **8,** 773–780 (1996).

39. Pentcheva-Hoang, T., Chen, L., Pardoll, D. M. & Allison, J. P. Programmed death-1 concentration at the immunological synapse is determined by ligand affinity and availability. *Proc Natl Acad Sci USA* **104,** 17765–17770 (2007).

40. Hino, R. *et al.* Tumor cell expression of programmed cell death-1 ligand 1 is a prognostic factor for malignant melanoma. *Cancer* **116,** 1757–1766 (2010).

41. Geng, L. *et al.* B7-H1 up-regulated expression in human pancreatic carcinoma tissue associates with tumor progression. *Journal of Cancer Research and Clinical Oncology* **134,** 1021–1027 (2008).

42. Parsa, A. T. *et al.* Loss of tumor suppressor PTEN function increases B7-H1 expression and immunoresistance in glioma. *Nat Med* **13,** 84–88 (2007).

43. Ghebeh, H. *et al.* The B7-H1 (PD-L1) T lymphocyte-inhibitory molecule is expressed in breast cancer patients with infiltrating ductal carcinoma: correlation with important high-

risk prognostic factors. *Neoplasia* **8,** 190–198 (2006).

44. Wu, C. *et al.* Immunohistochemical localization of programmed death-1 ligand-1 (PD-L1) in gastric carcinoma and its clinical significance. *Acta Histochem.* **108,** 19–24 (2006).

45. Ohigashi, Y. *et al.* Clinical significance of programmed death-1 ligand-1 and programmed death-1 ligand-2 expression in human esophageal cancer. *Clin. Cancer Res.* **11,** 2947–2953 (2005).

46. Strome, S. E. *et al.* B7-H1 blockade augments adoptive T-cell immunotherapy for squamous cell carcinoma. *Cancer Research* **63,** 6501–6505 (2003).

47. Hamanishi, J. *et al.* Programmed cell death 1 ligand 1 and tumor-infiltrating CD8+ T lymphocytes are prognostic factors of human ovarian cancer. *Proc Natl Acad Sci USA* **104,** 3360–3365 (2007).

48. Thompson, R. H. *et al.* Costimulatory molecule B7-H1 in primary and metastatic clear cell renal cell carcinoma. *Cancer* **104,** 2084–2091 (2005).

49. Dong, H. *et al.* Tumor-associated B7-H1 promotes T-cell apoptosis: a potential mechanism of immune evasion. *Nat Med* **8,** 793–800 (2002).

50. Iwai, Y. *et al.* Involvement of PD-L1 on tumor cells in the escape from host immune system and tumor immunotherapy by PD-L1 blockade. *Proc Natl Acad Sci USA* **99,** 12293–12297 (2002).

51. Azuma, T. *et al.* B7-H1 is a ubiquitous antiapoptotic receptor on cancer cells. *Blood* **111,** 3635–3643 (2008).

52. Hirano, F. *et al.* Blockade of B7-H1 and PD-1 by monoclonal antibodies potentiates cancer therapeutic immunity. *Cancer Research* **65,** 1089–1096 (2005).

53. Okudaira, K. *et al.* Blockade of B7-H1 or B7-DC induces an anti-tumor effect in a mouse pancreatic cancer model. *Int. J. Oncol.* **35,** 741–749 (2009).

54. Blank, C. *et al.* PD-L1/B7H-1 inhibits the effector phase of tumor rejection by T cell receptor (TCR) transgenic CD8+ T cells. *Cancer Research* **64,** 1140–1145 (2004).

55. Hodi, F. S. *et al.* Long-term survival of ipilimumab-naive patients (pts) with advanced melanoma (MEL) treated with nivolumab (anti-PD-1, BMS-936558, ONO-4538) in a phase I trial. *J Clin Oncol* **32,** abstr 9002 (2014).

56. Woo, S.-R. *et al.* Immune inhibitory molecules LAG-3 and PD-1 synergistically regulate T-cell function to promote tumoral immune escape. *Cancer Research* **72,** 917–927 (2012).

57. Duraiswamy, J., Kaluza, K. M., Freeman, G. J. & Coukos, G. Dual Blockade of PD-1 and CTLA-4 Combined with Tumor Vaccine Effectively Restores T Cell Rejection Function in Tumors. *Cancer Research* (2013). doi:10.1158/0008-5472.CAN-12-4100

58. Okazaki, T. *et al.* PD-1 and LAG-3 inhibitory co-receptors act synergistically to prevent autoimmunity in mice. *J Exp Med* **208,** 395–407 (2011).

59. Takeda, K. *et al.* Combination therapy of established tumors by antibodies targeting immune activating and suppressing molecules. *J Immunol* **184,** 5493–5501 (2010).

60. Curran, M. A., Montalvo, W., Yagita, H. & Allison, J. P. PD-1 and CTLA-4 combination blockade expands infiltrating T cells and reduces regulatory T and myeloid cells within B16 melanoma tumors. *Proceedings of the National Academy of Sciences* **107,** 4275–4280 (2010).

61. Kocak, E. *et al.* Combination therapy with anti-CTL antigen-4 and anti-4-1BB antibodies enhances cancer immunity and reduces autoimmunity. *Cancer Research* **66,** 7276–7284 (2006).

62. Wolchok, J. D. *et al.* Nivolumab plus Ipilimumab in Advanced Melanoma. *N Engl J Med* (2013). doi:10.1056/NEJMoa1302369

63. Sznol, M. *et al.* Survival, response duration, and activity by BRAF mutation (MT) status of nivolumab (NIVO, anti-PD-1, BMS-936558, ONO-4538) and ipilimumab (IPI) concurrent therapy in advanced melanoma (MEL). *J Clin Oncol* **32,** abstr LBA9003 (2014).

64. Antonia, S. J. *et al.* Nivolumab (anti-PD-1; BMS-936558, ONO-4538) and ipilimumab in first-line NSCLC: Interim phase I results. *J Clin Oncol* **32,** abstr 8023 (2014).

65. Hammers, H.-J. *et al.* Phase I study of nivolumab in combination with ipilimumab in metastatic renal cell carcinoma (mRCC). *J Clin Oncol* **32,** abstr 4504 (2014).

66. Nivolumab in combination with ipilimumab. From fda.gov at http://www.fda.gov/Drugs/InformationOnDrugs/ApprovedDrugs/ucm465274.htm

67. Ben-Eliyahu, S., Page, G. G., Yirmiya, R. & Shakhar, G. Evidence that stress and surgical interventions promote tumor development by suppressing natural killer cell activity. *Int. J. Cancer* **80,** 880–888 (1999).

68. Liakou, C. I. *et al.* CTLA-4 blockade increases IFNg-producing CD4+ICOShi cells to shift the ratio of effector to regulatory T cells in cancer patients. *Proc Natl Acad Sci USA* **105**, 14987-14992 (2008).

69. Carthon, B. C. *et al.* Preoperative CTLA-4 blockade: tolerability and immune monitoring in the setting of a presurgical clinical trial. *Clinical cancer research : an official journal of the American Association for Cancer Research* **16**, 2861-2871, doi:10.1158/1078-0432.CCR-10-0569 (2010).

70. Tang, D. N. *et al.* Increased Frequency of ICOS+ CD4 T Cells as a Pharmacodynamic Biomarker for Anti-CTLA-4 Therapy *Cancer Immunol Res* **1**, 229–234 (2013).

71. Chen, H. *et al.* CD4 T Cells Require ICOS-Mediated PI3K Signaling to Increase T-Bet Expression in the Setting of Anti-CTLA-4 Therapy. *Cancer Immunol Res* **2**, 1-10 (2014).

72. Thall, PF, Simon RM, Estey EH: Bayesian sequential monitoring designs for single-arm clinical trials with multiple outcomes. *Stat Med* **14**, 357-79, (1995).

73. Thall, PF, Simon, R, Estey, EH: New statistical strategy for monitoring safety and

efficacy in single-arm clinical trials. *J. Clinical Oncology* **14**, 296-303 (1996).

74. Thall, PF and Sung, H-G: Some extensions and applications of a Bayesian strategy for monitoring multiple outcomes in clinical trials. *Statistics in Medicine* **17**, 1563-1580 (1998).

12 Appendices

Appendix 1 Melanoma of the Skin Aging

American Joint Committee on Cancer

Melanoma of the Skin Staging

7th EDITION

Definitions

Primary Tumor (T)

- **TX** Primary tumor cannot be assessed (for example, curettaged or severely regressed melanoma)
- **T0** No evidence of primary tumor
- **Tis** Melanoma in situ
- **T1** Melanomas 1.0 mm or less in thickness
- **T2** Melanomas 1.01–2.0 mm
- **T3** Melanomas 2.01–4.0 mm
- **T4** Melanomas more than 4.0 mm

NOTE: a and b subcategories of T are assigned based on ulceration and number of mitoses per mm², as shown below:

T CLASSIFICATION	THICKNESS (mm)	ULCERATION STATUS/MITOSES
T1	≤1.0	a: w/o ulceration and mitosis <1/mm²<br>b: with ulceration or mitoses ≥1/mm²
T2	1.01–2.0	a: w/o ulceration<br>b: with ulceration
T3	2.01–4.0	a: w/o ulceration<br>b: with ulceration
T4	>4.0	a: w/o ulceration<br>b: with ulceration

Regional Lymph Nodes (N)

- **NX** Patients in whom the regional nodes cannot be assessed (for example, previously removed for another reason)
- **N0** No regional metastases detected
- **N1-3** Regional metastases based upon the number of metastatic nodes and presence or absence of intralymphatic metastases (in transit or satellite metastases)

NOTE: N1–3 and a–c subcategories assigned as shown below:

N CLASSIFICATION	NO. OF METASTATIC NODES	NODAL METASTATIC MASS
N1	1 node	a: micrometastasis[1]<br>b: macrometastasis[2]
N2	2–3 nodes	a: micrometastasis[1]<br>b: macrometastasis[2]<br>c: in transit met(s)/satellite(s) *without* metastatic nodes
N3	4 or more metastatic nodes, or matted nodes, or in transit met(s)/satellite(s) with metastatic node(s)	

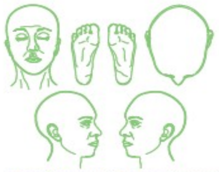

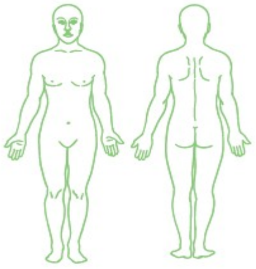

Distant Metastatis (M)

- **M0** No detectable evidence of distant metastases
- **M1a** Metastases to skin, subcutaneous, or distant lymph nodes
- **M1b** Metastases to lung
- **M1c** Metastases to all other visceral sites or distant metastases to any site combined with an elevated serum LDH

NOTE: Serum LDH is incorporated into the M category as shown below:

M CLASSIFICATION	SITE	SERUM LDH
M1a	Distant skin, subcutaneous, or nodal mets	Normal
M1b	Lung metastases	Normal
M1c	All other visceral metastases	Normal
	Any distant metastasis	Elevated

ANATOMIC STAGE/PROGNOSTIC GROUPS							
Clinical Staging[3]				Pathologic Staging[4]			
Stage 0	Tis	N0	M0	0	Tis	N0	M0
Stage IA	T1a	N0	M0	IA	T1a	N0	M0
Stage IB	T1b	N0	M0	IB	T1b	N0	M0
	T2a	N0	M0		T2a	N0	M0
Stage IIA	T2b	N0	M0	IIA	T2b	N0	M0
	T3a	N0	M0		T3a	N0	M0
Stage IIB	T3b	N0	M0	IIB	T3b	N0	M0
	T4a	N0	M0		T4a	N0	M0
Stage IIC	T4b	N0	M0	IIC	T4b	N0	M0
Stage III	Any T	≥ N1	M0	IIIA	T1-4a	N1a	M0
					T1-4a	N2a	M0
				IIIB	T1-4b	N1a	M0
					T1-4b	N2a	M0
					T1-4a	N1b	M0
					T1-4a	N2b	M0
					T1-4a	N2c	M0
				IIIC	T1-4b	N1b	M0
					T1-4b	N2b	M0
					T1-4b	N2c	M0
					Any T	N3	M0
Stage IV	Any T	Any N	M1	IV	Any T	Any N	M1

Notes

1 Micrometastases are diagnosed after sentinel lymph node biopsy and completion lymphadenectomy (if performed).

2 Macrometastases are defined as clinically detectable nodal metastases confirmed by therapeutic lymphadenectomy or when nodal metastasis exhibits gross extracapsular extension.

3 Clinical staging includes microstaging of the primary melanoma and clinical/radiologic evaluation for metastases. By convention, it should be used after complete excision of the primary melanoma with clinical assessment for regional and distant metastases.

4 Pathologic staging includes microstaging of the primary melanoma and pathologic information about the regional lymph nodes after partial or complete lymphadenectomy. Pathologic Stage 0 or Stage IA patients are the exception; they do not require pathologic evaluation of their lymph nodes.

ajcc

American Cancer Society

Financial support for AJCC 7th Edition Staging Posters provided by the American Cancer Society

Copyright 2009 American Joint Committee on Cancer • Printed with permission from the AJCC

Reference: American Joint Committee on Cancer. (2009). 7th Edition of AJCC Melanoma Staging System. Retrieved 19 April 2012 from http://www.cancerstaging.org/staging/posters/melanoma8.5x11.pdf.

Appendix 2 RECIST 1.1 Criteria

Measurability of Tumor Lesions at Baseline

Measurable lesion:

- A non nodal lesion that can be accurately measured in at least one dimension (longest dimension) of
  - ≥10 mm with MRI or CT when the scan slice thickness is no greater than 5mm. If the slice thickness is greater than 5mm, the minimum size of a measurable lesion must be at least double the slice thickness (e.g., if the slice thickness is 10 mm, a measurable lesion must be ≥20 mm).
  - ≥10 mm caliper/ruler measurement by clinical exam or medical photography.
  - ≥20 mm by chest x-ray.
- Additionally lymph nodes can be considered pathologically enlarged and measurable if
  - ≥15mm in the short axis when assessed by CT or MRI (slice thickness recommended to be no more than 5mm). At baseline and follow-up, only the short axis will be measured [Eisenhauer, 2009].

Non-measurable lesion:

- All other lesions including lesions too small to be considered measurable (longest diameter <10 mm or pathological lymph nodes with ≥ 10 mm and <15 mm short axis) as well as truly non-measurable lesions, which include: leptomeningeal disease, ascites, pleural or pericardial effusions, inflammatory breast disease, lymphangitic involvement of the skin or lung, abdominal masses/abdominal organomegaly identified by physical exam that is not measurable by reproducible imaging techniques [Eisenhauer, 2009].

Measurable disease:

- The presence of at least one measurable lesion. Palpable lesions that are not measurable by radiologic or photographic evaluations may not be utilized as the only measurable lesion.

Non-Measurable only disease:

- The presence of only non-measurable lesions.

Specifications by Methods of Measurements:

- The same diagnostic method, including use of contrast when applicable, must be used throughout the study to evaluate a lesion.

- All measurements should be taken and recorded in millimeters (mm), using a ruler or calipers.

- Ultrasound is not a suitable modality of disease assessment. If new lesions are identified by ultrasound, confirmation by CT or MRI is required.

- Fluorodeoxyglucose (FDG)-PET is generally not suitable for ongoing assessments of disease. However FDG-PET can be useful in confirming new sites of disease where a positive FDG-PET scans correlates with the new site of disease present on CT/MRI or when a baseline FDG-PET was previously negative for the site of the new lesion. FDG-PET may also be used in lieu of a standard bone scan providing coverage allows interrogation of all likely sites of bone disease and FDG-PET is performed at all assessments.

- If PET/CT is performed then the CT component can only be used for standard response assessments if performed to diagnostic quality, which includes the required anatomical coverage and prescribed use of contrast. The method of assessment should be noted as CT on the CRF.

Clinical Examination:

- Clinically detected lesions will only be considered measurable when they are superficial (e.g., skin nodules). In the case of skin lesions, documentation by color photography*,* including a ruler/calipers to measure the size of the lesion, is required.

  CT and MRI: Contrast enhanced CT with 5mm contiguous slices is recommended.

  Minimum size of a measurable baseline lesion should be twice the slice thickness, with a minimum lesion size of 10 mm when the slice thickness is 5 mm. MRI is acceptable, but when used, the technical specification of the scanning sequences should be optimized for the evaluation of the type and site of disease and lesions must be measured in the same anatomic plane by use of the same imaging examinations. Whenever possible the same scanner should be used. [Eisenhauer, 2009].

  X-ray: Should not be used for target lesion measurements owing to poor lesion definition. Lesions on chest X-ray may be considered measurable if they are clearly defined and surrounded by aerated lung; however chest CT is preferred over chest X-ray [Eisenhauer, 2009].

Evaluation of target lesions:

- Definitions for assessment of response for target lesion(s) are as follows:
  - Complete Response (CR): Disappearance of all target lesions. Any pathological lymph nodes must be <10mm in the short axis.
  - Partial Response (PR): At least a 30% decrease in the sum of the diameters of target lesions, taking as a reference, the baseline sum of the diameters (e.g. percent change from baseline).
  - Stable Disease (SD): Neither sufficient shrinkage to qualify for PR nor sufficient increase to qualify for progressive disease.

- Progressive Disease (PD): At least a 20% increase in the sum of the diameters of target lesions, taking as a reference, the smallest sum of diameters recorded since the treatment started (e.g. percent change from nadir, where nadir is defined as the smallest sum of diameters recorded since treatment start). In addition, the sum must have an absolute increase from nadir of 5mm.
- Not Applicable (NA): No target lesions at baseline.
- Not Evaluable (NE): Cannot be classified by one of the five preceding definitions.

- Note:

  - If lymph nodes are documented as target lesions the short axis is added into the sum of the diameters (e.g. sum of diameters is the sum of the longest diameters for non-nodal lesions and the short axis for nodal lesions). When lymph nodes decrease to non-pathological size (short axis <10mm) they should still have a measurement reported in order not to overstate progression.

  - If at a given assessment time point all target lesions identified at baseline are not assessed, sum of the diameters cannot be calculated for purposes of assessing CR, PR, or SD, or for use as the nadir for future assessments. However, the sum of the diameters of the assessed lesions and the percent change from nadir should be calculated to ensure that progression has not been documented. If an assessment of PD cannot be made, the response assessment should be NE.

  - All lesions (nodal and non-nodal) should have their measurements recorded even when very small (e.g 2 mm). If lesions are present but too small to measure, 5 mm should be recorded and should contribute to the sum of the diameters, unless it is likely that the lesion has disappeared in which case 0 mm should be reported.

  - If a lesion disappears and reappears at a subsequent time point it should continue to be measured. The response at the time when the lesion reappears will depend upon the status of the other lesions. For example, if the disease had reached a CR status then PD would be documented at the time of reappearance. However, if the response status was PR or SD, the diameter of the reappearing lesion should be added to the remaining diameters and response determined based on percent change from baseline and percent change from nadir

<u>Evaluation of non-target lesions:</u>

- Definitions for assessment of response for non-target lesions are as follows:

  - Complete Response (CR): The disappearance of all non-target lesions. All lymph nodes identified as a site of disease at baseline must be non-pathological (e.g. <10 mm short axis).

- Non-CR/Non-PD: The persistence of 1 or more non-target lesion(s) or lymph nodes identified as a site of disease at baseline ≥ 10 mm short axis.
- Progressive Disease (PD): Unequivocal progression of existing non-target lesions.
- Not Applicable (NA): No non-target lesions at baseline.
- Not Evaluable (NE): Cannot be classified by one of the four preceding definitions.

- Note:
  - In the presence of measurable disease, progression on the basis of solely non-target disease requires substantial worsening such that even in the presence of SD or PR in target disease, the overall tumor burden has increased sufficiently to merit discontinuation of therapy.
  - In the presence of non-measurable only disease consideration should be given to whether or not the increase in overall disease burden is comparable in magnitude to the increase that would be required to declare PD for measurable disease.
  - Sites of non-target lesions, which are not assessed at a particular time point based on the assessment schedule, should be excluded from the response determination (e.g. non-target response does not have to be "Not Evaluable").

Confirmation of Response:

- To be assigned a status of PR or CR, a confirmatory disease assessment should be performed no less than 4 weeks (28 days) after the criteria for response are first met.

Overall Response Criteria:

- Table 7 presents the overall response at an individual time point for all possible combinations of tumor responses in target and non-target lesions with or without the appearance of new lesions for subjects with measurable disease at baseline.

Table 7 Evaluation of Overall Response for Subjects with Measurable Disease at Baseline

Target Lesions	Non-Target Lesions	New Lesions	Overall Response
CR	CR or NA	No	CR
CR	Non-CR/Non-PD or NE	No	PR
PR	Non-PD or NA or NE	No	PR
SD	Non-PD or NA or NE	No	SD
NE	Non-PD or NA or NE	No	NE
PD	Any	Yes or No	PD
Any	PD	Yes or No	PD
Any	Any	Yes	PD

CR=complete response, PR = partial response, SD=stable disease, PD=progressive disease, NA= Not applicable, and NE=Not Evaluable

- Note:
    - Subjects with a global deterioration of health status requiring discontinuation of treatment without objective evidence of disease progression at that time should be classified as having "symptomatic deterioration." Objective response status is determined by evaluations of disease burden. Every effort should be made to document the objective progression even after discontinuation of treatment.
    - In some circumstances, it may be difficult to distinguish residual disease from normal tissue.

  When the evaluation of CR depends on this determination, it is recommended that the residual lesion be investigated (fine needle aspirate/biopsy) to confirm the CR.

Appendix 3 Guidance on Contraception

Guidance on Contraception

ACCEPTABLE METHODS FOR PROTOCOLS WITH A TERATOGENIC DRUG OR WHEN THERE IS INSUFFICIENT INFORMATION TO DETERMINE TERATOGENICITY

(CHOOSE ONE OF THE FOLLOWING 3 OPTIONS)

OPTION 1: Any TWO of the following methods

- ☐ Hormonal Methods of Contraception
- ☐ IUD
- ☐ Vasectomy
- ☐ Tubal Ligation
- ☐ A barrier method (Female of Male condom with spermicide, cervical cap with spermicide, or diaphragm with spermicide)

OPTION 2: Male condom (with spermicide) and diaphragm

OPTION 3: Male condom (with spermicide) and cervical cap

UNACCEPTABLE METHODS OF CONTRACEPTION:

- Abstinence (including periodic abstinence)
- No method
- Withdrawal
- Rhythm
- Vaginal Sponge
- Any barrier method without spermicide
- Spermicide
- Progestin-Only Pills

Concomitant Use of Female and Male Condom in countries where spermicide is not available, use of a male condom without spermicide in conjunction with a hormonal method, IUD, or tubal ligation will be acceptable to fulfill this recommendation. Any barrier method when used alone (without spermicide) or the concomitant use of a female and male condom, are not considered sufficient methods of contraception, as they carry a failure rate of > 1%.

Women of childbearing potential (WOCBP) receiving nivolumab will be instructed to adhere to contraception for a period of 24 weeks after the last dose of investigational product. Men receiving nivolumab and/or relatlimab and who are sexually active with WOCBP will be instructed to adhere to contraception for a period of 33 weeks after the last dose of investigational product. These durations have been calculated using the upper limit of the half-life for nivolumab (25 days) and are based on the protocol requirement that WOCBP use contraception for 5 half-lives plus 30 days and men who are sexually active with WOCBP use contraception for 5 half-lives plus 90 days.

Additional Pregnancy Test and Contraception Guidance:

CONTRACEPTION GUIDANCE FOR FEMALE PARTICIPANTS OF CHILD BEARING POTENTIAL

One of the highly effective methods of contraception listed below is required during study duration and until the end of relevant systemic exposure, defined as 24 weeks after the end of study treatment.*

**Highly Effective Contraceptive Methods That Are User Dependent**<br><br>*Failure rate of <1% per year when used consistently and correctly.*[a]
• Combined (estrogen- and progestogen-containing) hormonal contraception associated with inhibition of ovulation[b]<br>– oral<br>– intravaginal<br>– transdermal
• Progestogen-only hormonal contraception associated with inhibition of ovulation[b]<br>– oral<br>– injectable
**Highly Effective Methods That Are User Independent**
• Implantable progestogen-only hormonal contraception associated with inhibition of ovulation [b]<br>• Hormonal methods of contraception including oral contraceptive pills containing a combination of estrogen and progesterone, vaginal ring, injectables, implants and intrauterine hormone-releasing system (IUS)[c]<br>• Intrauterine device (IUD)[c]<br>• Bilateral tubal occlusion
• Vasectomized partner<br><br>*A vasectomized partner is a highly effective contraception method provided that the partner is the sole male sexual partner of the WOCBP and the absence of sperm has been confirmed. If not, an additional highly effective method of contraception should be used.*
• Sexual abstinence<br><br>*Sexual abstinence is considered a highly effective method only if defined as refraining from heterosexual intercourse during the entire period of risk associated with the study drug. The reliability of sexual abstinence needs to be evaluated in relation to the duration of the study and the preferred and usual lifestyle of the participant.*<br><br>• It is not necessary to use any other method of contraception when complete abstinence is elected.<br>• WOCBP participants who choose complete abstinence must continue to have pregnancy tests, as specified in Section 2.<br>• Acceptable alternate methods of highly effective contraception must be discussed in the event that the WOCBP participants chooses to forego complete abstinence

NOTES:
[a] Typical use failure rates may differ from those when used consistently and correctly. Use should be consistent with local regulations regarding the use of contraceptive methods for participants participating in clinical studies.<br>[b] Hormonal contraception may be susceptible to interaction with the study drug, which may reduce the efficacy of the contraceptive method. Hormonal contraception is permissible only when there is sufficient evidence that the IMP and other study medications will not alter hormonal exposures such that contraception would be ineffective or result in increased exposures that could be potentially hazardous. In this case, alternative methods of contraception should be utilized.<br>[c] Intrauterine devices and intrauterine hormone releasing systems are acceptable methods of contraception in the absence of definitive drug interaction studies when hormone exposures from intrauterine devices do not alter contraception effectiveness

**Unacceptable Methods of Contraception***
• Male or female condom with or without spermicide. Male and female condoms cannot be used simultaneously<br>• Diaphragm with spermicide<br>• Cervical cap with spermicide<br>• Vaginal Sponge with spermicide<br>• Progestogen-only oral hormonal contraception, where inhibition of ovulation is not the primary mechanism of action<br>• Periodic abstinence (calendar, symptothermal, post-ovulation methods)<br>• Withdrawal (coitus interruptus).<br>• Spermicide only<br>• Lactation amenorrhea method (LAM)

*** Local laws and regulations may require use of alternative and/or additional contraception methods.**

CONTRACEPTION GUIDANCE FOR MALE PARTICIPANTS WITH PARTNER(S) OF CHILD BEARING POTENTIAL.

Male participants with female partners of childbearing potential are eligible to participate if they agree to the following during the treatment and until the end of relevant systemic exposure.

- Inform any and all partner(s) of their participation in a clinical drug study and the need to comply with contraception instructions as directed by the investigator.
- Male participants are required to use a condom for study duration and until end of relevant systemic exposure defined as 33 weeks after the end of study treatment.
- Female partners of males participating in the study to consider use of effective methods of contraception until the end of relevant systemic exposure, defined as 7 months after the end of treatment in the male participant.
- Male participants with a pregnant or breastfeeding partner must agree to remain abstinent from penile vaginal intercourse or use a male condom during each episode of penile penetration during the treatment and until 33 weeks after the end of study treatment.

Refrain from donating sperm for the duration of the study treatment and until 7 months after the end of study treatment.

Appendix 4: GI Adverse Event Management Algorithm

GI Adverse Event Management Algorithm

Rule out non-inflammatory causes. If non-inflammatory cause is identified, treat accordingly and continue I-O therapy. Opiates/narcotics may mask symptoms of perforation. Infliximab should not be used in cases of perforation or sepsis.

Grade of Diarrhea/ Colitis (NCI CTCAE v4)	Management	Follow-up
**Grade 1**<br>Diarrhea:< 4 stools/day over baseline;<br>Colitis: asymptomatic	• Continue I-O therapy per protocol<br>• Symptomatic treatment	• Close monitoring for worsening symptoms.<br>• Educate patient to report worsening immediately<br>If worsens:<br>• Treat as Grade (G) 2 or 3/4
**Grade 2**<br>Diarrhea: 4-6 stools per day over baseline; IV fluids indicated <24 hours (hrs); not interfering with ADL<br>Colitis: abdominal pain; blood in stool	• Delay I-O therapy per protocol<br>• Symptomatic treatment	If improves to grade 1:<br>• Resume I-O therapy per protocol<br>If persists > 5-7 days or recur:<br>• 0.5-1.0 mg/kg/day methylprednisolone or oral equivalent<br>• When symptoms improve to grade 1, taper steroids over at least 1 month, consider prophylactic antibiotics for opportunistic infections, and resume I-O therapy per protocol.<br>If worsens or persists > 3-5 days with oral steroids:<br>• Treat as grade 3/4
**Grade 3-4**<br>Diarrhea (G3): ≥7 stools per day over baseline; incontinence; IV fluids ≥24 hrs; interfering with activities of daily living (ADL)<br>Colitis (G3): severe abdominal pain, medical intervention indicated, peritoneal signs<br>G4: life-threatening, perforation	• Discontinue I-O therapy per protocol<br>• 1.0 to 2.0 mg/kg/day methylprednisolone IV or IV equivalent<br>• Add prophylactic antibiotics for opportunistic infections<br>• Consider lower endoscopy	If improves:<br>• Continue steroids until grade 1, then taper over at least 1 month<br>If persists > 3-5 days, or recurs after improvement:<br>• Add infliximab 5 mg/kg (if no contraindication). Note: Infliximab should not be used in cases of perforation or sepsis

Patients on IV steroids may be switched to an equivalent dose of oral corticosteroids (e.g. prednisone) at start of tapering or earlier, once sustained clinical improvement is observed. Lower bioavailability of oral corticosteroids should be taken into account when switching to the equivalent dose of oral corticosteroids.

Appendix 5: Renal Adverse Event Management Algorithm

Renal Adverse Event Management Algorithm

Rule out non-inflammatory causes. If non-inflammatory cause, treat accordingly and continue I-O therapy

Grade of Creatinine Elevation (NCI CTCAE v4)	Management	Follow-up
**Grade 1**<br>Creatinine > upper limit of normal (ULN) and > than baseline but ≤ 1.5x baseline	• Continue I-O therapy per protocol<br>• Monitor creatinine weekly	<u>If returns to baseline</u>:<br>•Resume routine creatinine monitoring per protocol<br><u>If worsens:</u><br>•Treat as Grade 2 or 3/4
**Grade 2-3**<br>Creatinine > 1.5x baseline to ≤ 6x ULN	• Delay I-O therapy per protocol<br>• Monitor creatinine every 2-3 days<br>• 0.5 to 1.0 mg/kg/day methylprednisolone IV or oral equivalent<br>• Consider renal biopsy	<u>If returns to Grade 1:</u><br>•Taper steroids over at least 1 month, consider prophylactic antibiotics for opportunistic infections, and resume I-O therapy and routine creatinine monitoring per protocol<br><br><u>If elevations persist > 7 days or worsen:</u><br>•Treat as Grade 4
**Grade 4**<br>Creatinine > 6x ULN	• Discontinue I-O therapy per protocol<br>• Monitor creatinine daily<br>• 1.0-2.0 mg/kg/day methylprednisolone IV or IV equivalent<br>• Consult nephrologist<br>• Consider renal biopsy	<u>If returns to Grade 1</u> : Taper steroids over at least 1 month and add prophylactic antibiotics for opportunistic infections

Patients on IV steroids may be switched to an equivalent dose of oral corticosteroids (e.g. prednisone) at start of tapering or earlier, once sustained clinical improvement is observed. Lower bioavailability of oral corticosteroids should be taken into account when switching to the equivalent dose of oral corticosteroids.

Appendix 6: Pulmonary Adverse Event Management Algorithm

Pulmonary Adverse Event Management Algorithm

Rule out non-inflammatory causes. If non-inflammatory cause, treat accordingly and continue I-O therapy. Evaluate with imaging and pulmonary consultation.

Grade of Pneumonitis (NCI CTCAE v4)	Management	Follow-up
**Grade 1**<br>Radiographic changes only	• Consider delay of I-O therapy<br>• Monitor for symptoms every 2-3 days<br>• Consider Pulmonary and Infectious Disease (ID) consults	• Re-image at least every 3 weeks<br><u>If worsens:</u><br>• Treat as Grade 2 or 3-4
**Grade 2**<br>Mild to moderate new symptoms	• Delay I-O therapy per protocol<br>• Pulmonary and ID consults<br>• Monitor symptoms daily, consider hospitalization<br>• 1.0 mg/kg/day methyl-prednisolone IV or oral equivalent<br>• Consider bronchoscopy, lung biopsy	• Re-image every 1-3 days<br><u>If improves</u>:<br>• When symptoms return to near baseline, taper steroids over at least 1 month and then resume I-O therapy per protocol and consider prophylactic antibiotics<br><u>If not improving after 2 weeks or worsening:</u><br>• Treat as Grade 3-4
**Grade 3-4**<br>Severe new symptoms; New/worsening hypoxia; Life-threatening	• Discontinue I-O therapy per protocol<br>• Hospitalize<br>• Pulmonary and ID consults<br>• 2-4 mg/kg/day methylprednisolone IV or IV equivalent<br>• Add prophylactic antibiotics for opportunistic infections<br>• Consider bronchoscopy, lung biopsy	<u>If improves to baseline:</u><br>• Taper steroids over at least 6 weeks<br><u>If not improving after 48 hours or worsening:</u><br>• Add additional immunosuppression (e.g. infliximab, cyclophosphamide, intravenous immunoglobulin (IVIG), or mycophenolate mofetil)

Patients on IV steroids may be switched to an equivalent dose of oral corticosteroids (e.g. prednisone) at start of tapering or earlier, once sustained clinical improvement is observed. Lower bioavailability of oral corticosteroids should be taken into account when switching to the equivalent dose of oral corticosteroids.

Appendix 7: Hepatic Adverse Event Management Algorithm

Hepatic Adverse Event Management Algorithm

Rule out non-inflammatory causes. If non-inflammatory cause, treat accordingly and continue I-O therapy. Consider imaging for obstruction.

Grade of Liver Test Elevation (NCI CTCAE v4)	Management	Follow-up
**Grade 1**<br>AST or ALT > ULN to 3.0 x ULN <u>and/or</u> Total bilirubin (T. bili) > ULN - 1.5 x ULN	• Continue I-O therapy per protocol	• Continue liver function tests (LFT) monitoring per protocol<br><u>If worsens:</u><br>• Treat as Grade 2 or 3-4
**Grade 2**<br>AST or ALT > 3.0 to ≤ 5 x ULN <u>and/or</u> T. bili > 1.5 to ≤ 3 x ULN	• Delay I-O therapy per protocol<br>• Increase frequency of monitoring to every 3 days	<u>If returns to baseline</u>:<br>•Resume routine monitoring, resume I-O therapy per protocol<br><br><u>If elevations persist > 5-7 days or worsen :</u><br>• 0.5-1 mg/kg/day methylprednisolone or oral equivalent and when LFT returns to grade 1 or baseline, taper steroids over at least 1 month, consider prophylactic antibiotics for opportunistic infections, and resume I-O therapy per protocol
**Grade 3-4**<br>AST or ALT > 5 x ULN <u>and /or</u> T.bili >3 x ULN	• Discontinue I-O therapy*<br>• Increase frequency of monitoring to every 1-2 days<br>• 1.0 to 2.0 mg/kg/day methylprednisolone IV or IV equivalent**<br>• Add prophylactic antibiotics for opportunistic infections<br>• Consult gastroenterologist	<u>If returns to grade 2</u>:<br>• Taper steroids over at least 1 month<br><br><u>If does not improve in >3-5 days, worsens or rebounds:</u><br>• Add mycophenolate mofetil 1 gram (g) twice daily (BID)<br>• If no response within an additional 3-5 days, consider other immunosuppressants per local guidelines

Patients on IV steroids may be switched to an equivalent dose of oral corticosteroids (e.g. prednisone) at start of tapering or earlier, once sustained clinical improvement is observed. Lower bioavailability of oral corticosteroids should be taken into account when switching to the equivalent dose of oral corticosteroids.

***I-O therapy may be delayed rather than discontinued if AST/ALT ≤ 8 x ULN and T.bili ≤ 5 x ULN.**

**The recommended starting dose for grade 4 hepatitis is 2 mg/kg/day methylprednisolone IV.

Appendix 8: Endocrinopathy Adverse Event Management Algorithm

Endocrinopathy Management Algorithm

Rule out non-inflammatory causes. If non-inflammatory cause, treat accordingly and continue I-O therapy. Consider visual field testing, endocrinology consultation, and imaging.

Asymptomatic thyroid stimulating hormone (TSH) elevation →

- Continue I-O therapy per protocol
- If TSH < 0.5 x lower limit of normal (LLN), or TSH > 2 x ULN, or consistently out of range in 2 subsequent measurements: include free thyroxine (fT4) at subsequent cycles as clinically indicated; consider endocrinology consult

Symptomatic endocrinopathy →

- Evaluate endocrine function
- Consider pituitary scan

Symptomatic with abnormal lab/pituitary scan:
- Delay I-O therapy per protocol
- 1-2 mg/kg/day methylprednisolone IV or by mouth (PO) equivalent
- Initiate appropriate hormone therapy

No abnormal lab/pituitary MRI scan but symptoms persist:
- Repeat labs in 1-3 weeks /MRI in 1 month

→ If improves (with or without hormone replacement):
- Taper steroids over at least 1 month and consider prophylactic antibiotics for opportunistic infections
- Resume I-O therapy per protocol
- Patients with adrenal insufficiency may need to continue steroids with mineralocorticoid component

Suspicion of adrenal crisis (e.g. severe dehydration, hypotension, shock out of proportion to current illness →

- Delay or discontinue I-O therapy per protocol
- Rule out sepsis
- Stress dose of IV steroids with mineralocorticoid activity
- IV fluids
- Consult endocrinologist
- If adrenal crisis ruled out, then treat as above for symptomatic endocrinopathy

Patients on IV steroids may be switched to an equivalent dose of oral corticosteroids (e.g. prednisone) at start of tapering or earlier, once sustained clinical improvement is observed. Lower bioavailability of oral corticosteroids should be taken into account when switching to the equivalent dose of oral corticosteroids.

Appendix 9: Skin Adverse Event Management Algorithm

Skin Adverse Event Management Algorithm

Rule out non-inflammatory causes. If non-inflammatory cause, treat accordingly and continue I-O therapy.

Grade of Rash (NCI CTCAE v4)	Management	Follow-up
**Grade 1-2** Covering ≤ 30% body surface area (BSA)*	• Symptomatic therapy (e.g. antihistamines, topical steroids)<br>• Continue I-O therapy per protocol	<u>If persists > 1-2 weeks or recurs:</u><br>• Consider skin biopsy<br>• Delay I-O therapy per protocol<br>• Consider 0.5-1.0 mg/kg/day methylprednisolone IV or oral equivalent. Once improving , taper steroids over at least 1 month, consider prophylactic antibiotics for opportunistic infections, and resume I-O therapy per protocol<br><u>If worsens:</u><br>• Treat as Grade 3-4
**Grade 3-4** Covering >30% BSA; Life threatening consequences*	• Delay or discontinue I-O therapy per protocol<br>• Consider skin biopsy<br>• Dermatology consult<br>• 1.0-2.0 mg/kg/day IV methylprednisolone IV or IV equivalent	<u>If improves to Grade 1:</u><br>• Taper steroids over at least 1 month and add prophylactic antibiotics for opportunistic infections<br>• Resume I-O therapy per protocol

Patients on IV steroids may be switched to an equivalent dose of oral corticosteroids (e.g. prednisone) at start of tapering or earlier, once sustained clinical improvement is observed. Lower bioavailability of oral corticosteroids should be taken into account when switching to the equivalent dose of oral corticosteroids.
*Refer to NCI CTCAE v4 for term-specific grading criteria.

Appendix 10: Neurological Adverse Event Management Algorithm

Neurological Adverse Event Management Algorithm

Rule out non-inflammatory causes. If non-inflammatory cause, treat accordingly and continue I-O therapy.

Grade of Neurological Toxicity (NCI CTCAE v4)	Management	Follow-up
**Grade 1**<br>Asymptomatic or mild symptoms; Intervention not indicated	• Continue I-O therapy per protocol	Continue to monitor the patient.<br>If worsens:<br>• Treat as Grade 2 or 3-4
**Grade 2**<br>Moderate symptoms; Limiting instrumental ADL	• Delay I-O therapy per protocol<br>• Treat symptoms per local guidelines<br>• Consider 0.5 to 1.0 mg/kg/day methylprednisolone IV or PO equivalent	If improves to baseline:<br>•Resume I-O therapy per protocol when improved to baseline<br>If worsens:<br>•Treat as Grade 3-4
**Grade 3-4**<br>Severe symptoms; Limiting self-care ADL; Life-threatening	• Discontinue I-O therapy per protocol<br>• Obtain neurology consult<br>• Treat symptoms per local guidelines<br>• 1.0-2.0 mg/kg/day IV methylprednisolone IV or IV equivalent<br>• Add prophylactic antibiotics for opportunistic infections	If improves to Grade 2:<br>• Taper steroids over at least 1 month<br><br>If worsens or atypical presentation:<br>• Consider IVIG or other immunosuppressive therapies per local guidelines

Patients on IV steroids may be switched to an equivalent dose of oral corticosteroids (e.g. prednisone) at start of tapering or earlier, once sustained clinical improvement is observed. Lower bioavailability of oral corticosteroids should be taken into account when switching to the equivalent dose of oral corticosteroids.
