# Supplementary material for: Neoadjuvant relatlimab and nivolumab in resectable melanoma
Source: Nature. 2022 Oct 26;611(7934):155–60. doi: 10.1038/s41586-022-05368-8 (PMC9607737; doi:10.1038/s41586-022-05368-8)
Supplement: Supplementary file 2 — Reporting Summary [file 41586_2022_5368_MOESM2_ESM.pdf]

nature portfolio

Corresponding author(s): Rodabe Amaria

Last updated by author(s): August 1, 2022

Reporting Summary

Nature Portfolio wishes to improve the reproducibility of the work that we publish. This form provides structure for consistency and transparency in reporting. For further information on Nature Portfolio policies, see our Editorial Policies and the Editorial Policy Checklist.

Statistics

For all statistical analyses, confirm that the following items are present in the figure legend, table legend, main text, or Methods section.

n/a	Confirmed	
☐	☒	The exact sample size (n) for each experimental group/condition, given as a discrete number and unit of measurement
☐	☒	A statement on whether measurements were taken from distinct samples or whether the same sample was measured repeatedly
☐	☒	The statistical test(s) used AND whether they are one- or two-sided<br>*Only common tests should be described solely by name; describe more complex techniques in the Methods section.*
☐	☒	A description of all covariates tested
☐	☒	A description of any assumptions or corrections, such as tests of normality and adjustment for multiple comparisons
☐	☒	A full description of the statistical parameters including central tendency (e.g. means) or other basic estimates (e.g. regression coefficient) AND variation (e.g. standard deviation) or associated estimates of uncertainty (e.g. confidence intervals)
☐	☒	For null hypothesis testing, the test statistic (e.g. F, t, r) with confidence intervals, effect sizes, degrees of freedom and P value noted<br>*Give P values as exact values whenever suitable.*
☒	☐	For Bayesian analysis, information on the choice of priors and Markov chain Monte Carlo settings
☒	☐	For hierarchical and complex designs, identification of the appropriate level for tests and full reporting of outcomes
☒	☐	Estimates of effect sizes (e.g. Cohen's d, Pearson's r), indicating how they were calculated

Our web collection on statistics for biologists contains articles on many of the points above.

Software and code

Policy information about availability of computer code

Data collection	Clinical data collection via MDACC Prometheus Data Collection System.<br>Flow cytometry data collection software: BD FACSDiva software, version 8.0.1 for flow cytometry (Becton Dickinson & Company)<br>CyTOF data collection software: CyTOF Software v 7.0.8493 for mass cytometry (Fluidigm, now Standard Biotools)
Data analysis	Clinical data analysis by SAS 9.4 by Windows (Copyright © 2002-2012 by SAS Institute Inc., Cary, NC)<br>FlowJo v.10.5.3 (Becton Dickinson & Company); WorkFlow script for unsupervised clustering (Nowicka et al F1000 Research 2017), running on R version 3.5.2 (R Foundation for Statistical Computing)<br>R Foundation for Statistical Computing

For manuscripts utilizing custom algorithms or software that are central to the research but not yet described in published literature, software must be made available to editors and reviewers. We strongly encourage code deposition in a community repository (e.g. GitHub). See the Nature Portfolio guidelines for submitting code & software for further information.

Data

Policy information about availability of data

All manuscripts must include a data availability statement. This statement should provide the following information, where applicable:

- Accession codes, unique identifiers, or web links for publicly available datasets
- A description of any restrictions on data availability
- For clinical datasets or third party data, please ensure that the statement adheres to our policy

Full data to be provided upon request, there are no restrictions

Field-specific reporting

Please select the one below that is the best fit for your research. If you are not sure, read the appropriate sections before making your selection.

- [x] Life sciences
- [ ] Behavioural & social sciences
- [ ] Ecological, evolutionary & environmental sciences

For a reference copy of the document with all sections, see nature.com/documents/nr-reporting-summary-flat.pdf

Life sciences study design

All studies must disclose on these points even when the disclosure is negative.

Sample size	Sample size justification provided in the methods of the manuscript.
Data exclusions	There were no data exclusions
Replication	Data was not able to be replicated as this is a study using human subjects and data generated is unique to the study subject
Randomization	This was a single arm study with no randomization
Blinding	Blinding was not utilized as this is a single arm, non-randomized trial

Reporting for specific materials, systems and methods

We require information from authors about some types of materials, experimental systems and methods used in many studies. Here, indicate whether each material, system or method listed is relevant to your study. If you are not sure if a list item applies to your research, read the appropriate section before selecting a response.

Materials & experimental systems

n/a	Involved in the study	
[ ]	[x]	Antibodies
[x]	[ ]	Eukaryotic cell lines
[x]	[ ]	Palaeontology and archaeology
[x]	[ ]	Animals and other organisms
[ ]	[x]	Human research participants
[ ]	[x]	Clinical data
[x]	[ ]	Dual use research of concern

Methods

n/a	Involved in the study	
[x]	[ ]	ChIP-seq
[ ]	[x]	Flow cytometry
[x]	[ ]	MRI-based neuroimaging

Antibodies

Antibodies used	The anti PD-1 antibody nivolumab and anti LAG-3 antibody relatlimab were provided by the study sponsor Bristol-Myers Squibb as part of their investigational supply of agents. Relatlimab should be stored at 2°C to 8°C (36oF to 46oF) with protection from light. Do not freeze the drug product.<br>Relatlimab is to be administered combined with nivolumab in the same bag as a 60 minute IV infusion through a 0.2/1.2-[illegible]m pore size, low-protein-binding polyethersulfone membrane in-line filter at the protocol-specified doses. The Relatlimab and nivolumab injection can be diluted with 0.9% sodium chloride injection (normal saline), to protein concentrations no lower than 1.33 mg/mL. Detailed instructions for drug product dilution and administration are provided in the pharmacy manual for the clinical study
Validation	These antibodies were provided as part of BMS's investigational study supply

Human research participants

Policy information about studies involving human research participants

Population characteristics	Clinical stage III melanoma with resectable disease. Patients aged 18 and over, ECOG PS 0-1, normal organ function with no contra-indications to surgery.
Recruitment	Patients were enrolled at MD Anderson and Memorial Sloann Kettering in the Melanoma Clinics. Patients were offered either clinical trial enrollment or standard of care therapies. Patients were provided copies of the study informed consent document and were fully aware of risks prior to trial enrollment. As patients needed to fulfill inclusion criteria of trial, this could have caused selection bias.
Ethics oversight	IRB of MDACC and MSKCC provided ethics oversight. An informed consent statement was included in the Methods/Study Oversight section

Note that full information on the approval of the study protocol must also be provided in the manuscript.

Clinical data

Policy information about clinical studies
All manuscripts should comply with the ICMJE guidelines for publication of clinical research and a completed CONSORT checklist must be included with all submissions.

Clinical trial registration	NCT02519322
Study protocol	Available upon request
Data collection	9/19/2018 - 9/23/2020 for enrollment, patients followed for at least 1 year after date of last enrollment. Data was stored in a secure database sponsored by MD Anderson Cancer Center and was able to be accessed by staff at MSKCC for direct data input.
Outcomes	The primary outcome was assessment of pathologic response following neoadjuvant therapy as per the criteria of the INMC which is agreed upon pathologic response criteria utilized in melanoma neoadjuvant studies. Secondary outcomes including RECIST response, safety, RFS, EFS, and OS are standard outcome criteria utilized in neoadjuvant studies to describe characteristics of response. Correlation of immune profiling with response was exploratory in nature and dependent upon results of correlative studies.

Flow Cytometry

Plots

Confirm that:

☒ The axis labels state the marker and fluorochrome used (e.g. CD4-FITC).

☒ The axis scales are clearly visible. Include numbers along axes only for bottom left plot of group (a 'group' is an analysis of identical markers).

☒ All plots are contour plots with outliers or pseudocolor plots.

☒ A numerical value for number of cells or percentage (with statistics) is provided.

Methodology

Sample preparation	Sample preparation details can be found in the Methods section of the manuscript. Isolation and preparation of cells from peripheral blood and tissues<br>Whole blood was collected in tubes containing sodium heparin (BD Vacutainer), resuspended in PBS, layered atop Ficoll (StemCell Technologies) and centrifuged at 800 × g for 25 minutes. The interface peripheral blood mononuclear cells (PBMC) were harvested and washed twice with PBS and centrifuged at 500 × g for 10 minutes. Fresh tumor tissue was dissociated with GentleMACS system (Miltenyi Biotec). PBMC and tumor specimens destined for CyTOF analysis were stained for viability with 5 μmol/L cisplatin (Fluidigm) in PBS containing 1% BSA and then washed 3x. All specimens were resuspended in AB serum with 10% (vol/vol) DMSO for storage in liquid nitrogen until downstream assays were performed.
Instrument	BD LSRFortessa x20 flow cytometer (Becton Dickinson & Company);<br>Helios mass cytometer (Fluidigm, now Standard Biotools)
Software	Acquisition Software: BD FACSDiva software, version 8.0.1 for flow cytometry (Becton Dickinson & Company); CyTOF Software v 7.0.8493 for mass cytometry (Fluidigm, now Standard Biotools)<br>Analysis Software: FlowJo v.10.5.3 (Becton Dickinson & Company); WorkFlow script for unsupervised clustering (Nowicka et al F1000 Research 2017), running on R version 3.5.2 (R Foundation for Statistical Computing)
Cell population abundance	Sorting was not performed. Bulk tumor cells were procured, the immune fraction enriched via buffy layer, and immune cell-specific detection antibodies were used for cytometry analysis.
Gating strategy	Please see Extended Data Figure 5 to review the Flow Cytometry and CyTOF gating strategies.

☒ Tick this box to confirm that a figure exemplifying the gating strategy is provided in the Supplementary Information.
